# Versatile synthesis and biological evaluation of novel 3’-fluorinated purine nucleosides

**DOI:** 10.3762/bjoc.11.272

**Published:** 2015-12-09

**Authors:** Hang Ren, Haoyun An, Paul J Hatala, William C Stevens, Jingchao Tao, Baicheng He

**Affiliations:** 1 College of Chemistry and Molecular Engineering, Zhengzhou University, 100 Science Avenue, Zhengzhou 450001, China; 2 Granlen, Inc., 7864 Paseo Tulipero, Carlsbad, CA 92009, USA; 3 Molecular Oncology Laboratory, The University of Chicago Medical Center, Chicago, Illinois 60637, USA

**Keywords:** anticancer, 3’-fluororibonucleoside, purine nucleoside, 6-substituted purine, synthesis

## Abstract

A unified synthetic strategy accessing novel 3'-fluorinated purine nucleoside derivatives and their biological evaluation were achieved. Novel 3’-fluorinated analogues were constructed from a common 3’-deoxy-3’-fluororibofuranose intermediate. Employing Suzuki and Stille cross-coupling reactions, fifteen 3’-fluororibose purine nucleosides **1**–**15** and eight 3’-fluororibose 2-chloro/2-aminopurine nucleosides **16**–**23** with various substituents at position 6 of the purine ring were efficiently synthesized. Furthermore, 3’-fluorine analogs of natural products nebularine and 6-methylpurine riboside were constructed via our convergent synthetic strategy. Synthesized nucleosides were tested against HT116 (colon cancer) and 143B (osteosarcoma cancer) tumor cell lines. We have demonstrated 3’-fluorine purine nucleoside analogues display potent tumor cell growth inhibition activity at sub- or low micromolar concentration.

## Introduction

Antimetabolites are extremely useful for the treatment of cancers and viral infections and are one of the largest classes of drugs. Most antimetabolite drugs are nucleoside derivatives that substitute for endogenous nucleosides and prevent DNA and protein replication [1]. Many of the drugs described by the World Health Organization as "*essential medicines*" are nucleoside derivatives [2] and nearly 20% of all drugs for the treatment of cancers are nucleoside derivatives [3]. The design of new antimetabolites is an active field of research and several nucleoside derivatives have recently come to market such as gemcitabine, capecitabine, and decitabine [4–6]. Purine nucleoside analogues such as fludarabine, nelarabine, cladribine, and clofarabine are an important subset of nucleoside drugs [7–10]. Purine ribonucleosides substituted at position 6 have exhibited potent antimetabolite activity [11–13] and aryl or heterocyclic substituents have imparted cytostatic activities against various tumor cell-lines [14–16]. Moreover, some 6-heterocyclic substituted purine ribonucleosides also demonstrate strong antiviral activities [17]. Purine derivatives such as, 2’-β-*C*-methyl-6-substituted purine nucleosides exhibit promising anti-HCV activity by blocking RNA dependent RNA polymerase [18–20]. Design and synthesis of purine-based nucleosides are still needed to enable new therapies for the treatment of drug-resistant tumors and viruses.

One of the first antimetabolite drugs rationally designed from biochemical data was 5-fluorouracil. A hydrogen atom on uracil was replaced with a fluorine atom for specific reasons. The fluorine atom possesses unique characteristics; it enhances the lipophilicity of organic compounds and C–F bonds have low chemical reactivity imparting high enzymatic stability and resistance to metabolic processes. The high electronegativity of fluorine and the lipophilicity it imparts improve the bioavailability of fluorine-containing drugs. Relative to the unfluorinated derivative, fluorinated drugs have demonstrated favorable pharmacological, physicochemical, pharmacokinetic, pharmacodynamic and safety profiles for a number of compounds [21–23]. Several blockbuster drugs such as Lipitor^®^, Seretide^®^, Crestor^®^, Takepron^®^, Sustiva^®^, Celebrex^®^, and the recently described fluorapacin and azvudine [24–29], all contain fluorine atom(s). Not surprisingly, 20% of marketed drugs contain fluorine atom(s). Gemcitabine, the 2’-deoxy-2’,2’-difluorocytidine has been routinely utilized to treat solid tumors [30]. However, 3’-fluorine-modified nucleosides have not been well-studied because of the challenges associated with the synthesis of modified carbohydrate moieties [31–35]. As a result, 3’-fluoro-6-heterocyclic-substituted purine nucleosides are not well represented in the literature. We chose to explore the biological potential of synthetic purine analogues combining substitutions at position 6 and a 3’-fluorine. Novel 6-heterocyclic substituted purine 3’-deoxy-3’-fluororibonucleosides were designed to discover more selective and potent novel antiviral and anticancer therapeutics. Various fluorine-modified ribonucleoside derivatives were designed, synthesized, and tested. The preliminary results are presented herein.

**Figure 1:**
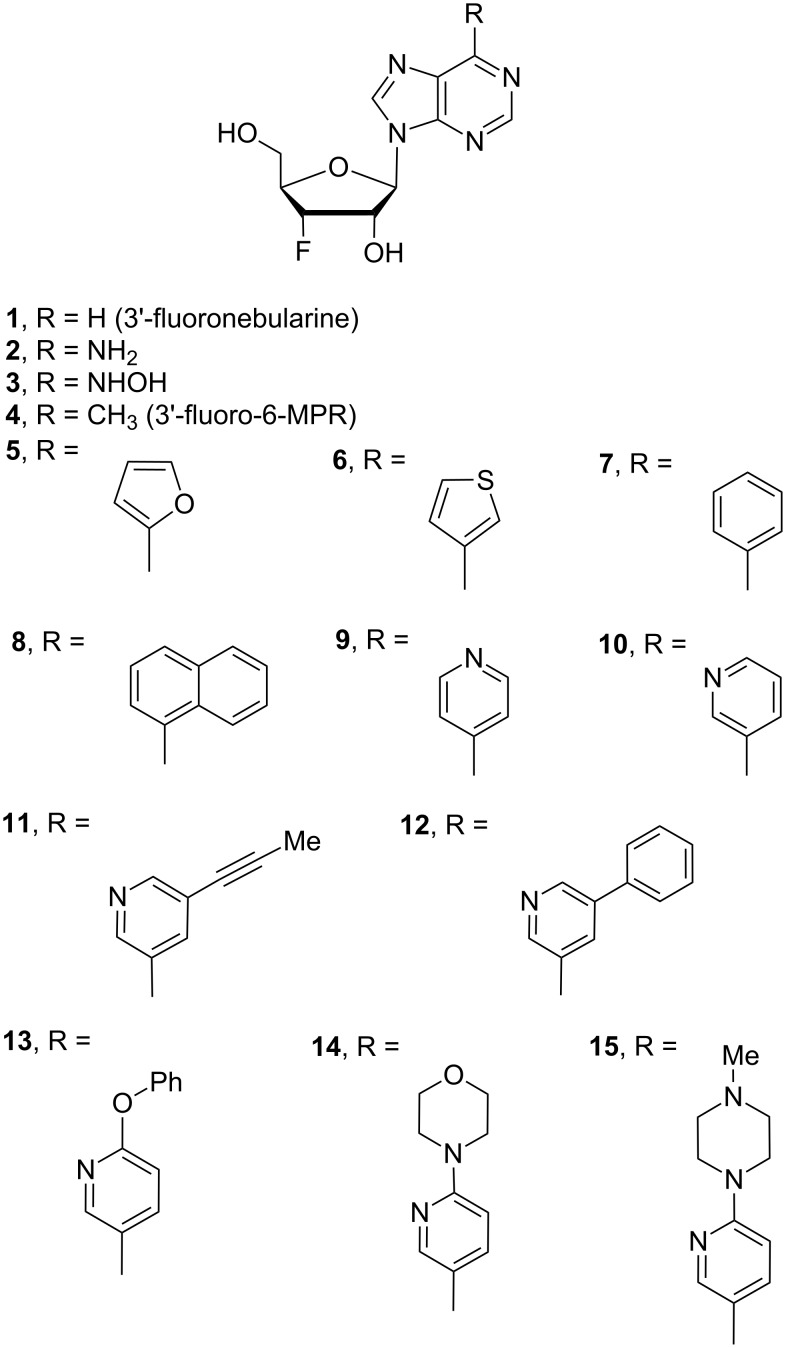
6-Subsituted purine 3’-deoxy-3’-fluororibosides **1**–**15**.

**Figure 2:**
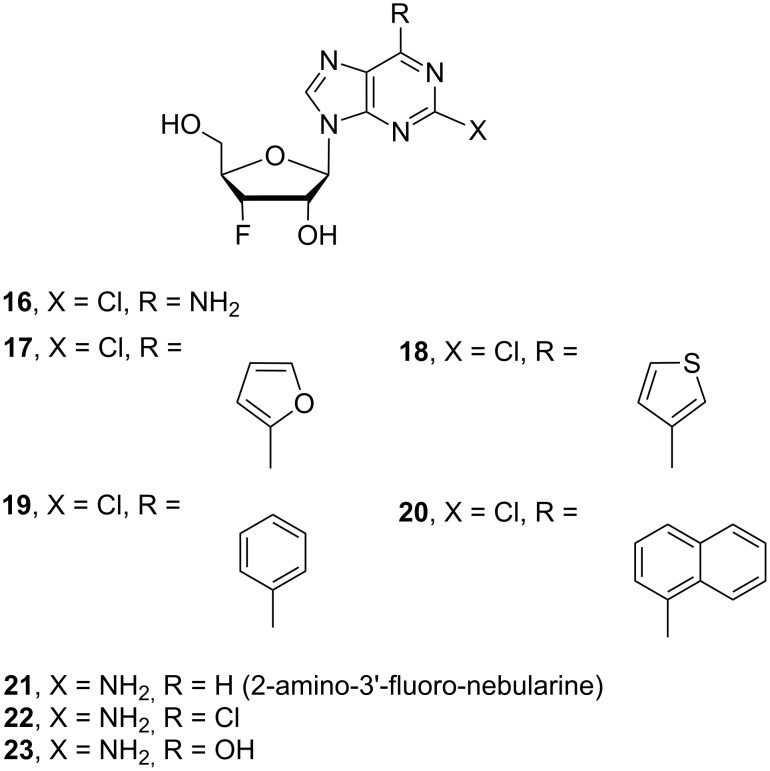
2-Chloro- and 2-aminopurine 3’-deoxy-3’-fluororibosides **16**–**23**.

We utilized the structural characteristics of 3’-fluorine and 6-substituted purine nucleosides to expand their biological application. Herein, we report the synthesis of twenty three 3’-deoxy-3’-fluoro-6-modified purine nucleoside derivatives, of which twenty one are novel compounds, including 6-substituted purine 3’-deoxy-3’-fluororibosides **1**–**15** (Figure 1), 2-chloro-6-substituted purine 3’-deoxy-3’-fluororibosides **16**–**20** and 2-amino-6-substituted purine 3’-deoxy-3’-fluororiboses **21**–**23** (Figure 2). In addition, their anticancer activity was evaluated.

## Results and Discussion

Current strategies to the synthesis of 3’-fluorine and 6-substituted purine require harsh conditions and laborious protecting group manipulation that results in low product yields. It was reported that 3’-deoxy-3’-fluoroadenosine (**2**) (Figure 1) was synthesized in 3.5% yield starting from adenosine [31–32]. This eight-step synthesis required harsh reaction conditions and HPLC purification of the final product. In addition, the product cannot be utilized for further derivatization to reach our objectives. This 3’-fluorine-modified adenosine **2** has also been synthesized starting from a well-protected xylofuranosyladenine derivative using a complicated strategy [33]. Similarly, 3’-deoxy-3’-fluoroguanosine (**23**, Figure 2) was isolated in 2% yield after protecting group manipulation from arabinoguanosine [34]. Complicated orthogonally protected adenosine and guanosine derivatives with three or four different protecting groups have also been used for the synthesis of compounds **2** and **23**, and the protocols required extensive manipulation of the protecting groups [35]. Compound **2** has also been synthesized starting from xylofuranoside by manipulating the protecting groups on the carbohydrate moiety [36]. De Clercq and co-workers [37] developed a protocol for the synthesis of 3’-fluororibofuranose in 10 steps, and it requires epoxide formation and ring opening as well as reversion of the hydroxy group on the sugar ring. Jeong and co-workers [38–40] synthesized fluorine-substituted ribofuranose but isolation required a challenging separation. Therefore, the synthetic challenges for preparing 3’-fluorine modified sugars and nucleosides have limited the synthesis and biological testing of these promising fluorine modified nucleoside derivatives. To open up research and therapeutic exploration of this class of compounds, we developed efficient synthetic routes for the construction of 3’-deoxy-3’-fluoroadenosine and guanosine derivatives with a number of different modifications on position 6.

**Figure 3:**
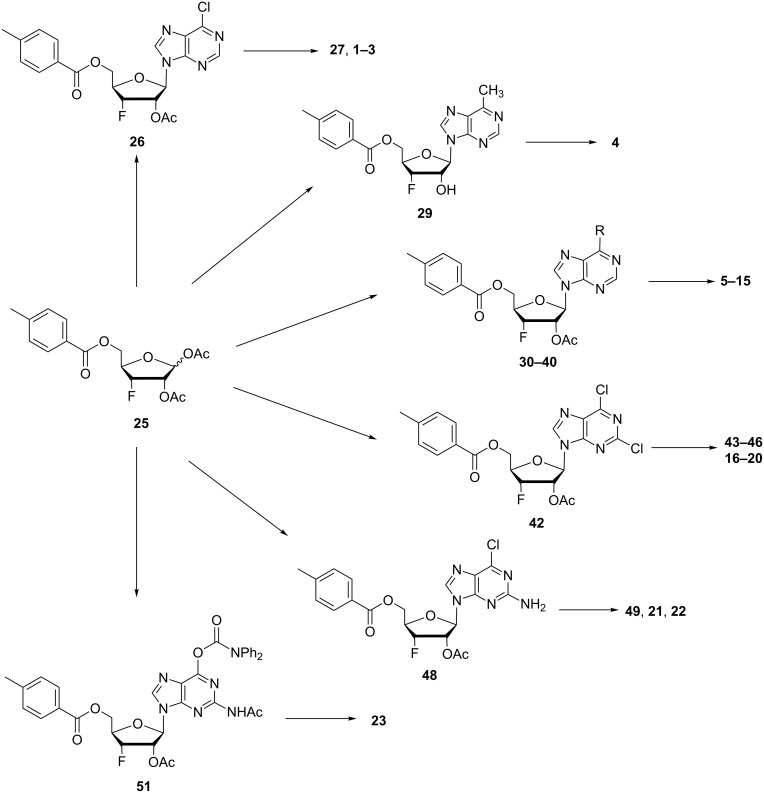
3’-Deoxy-3’-fluororibosides constructed from universal intermediate **25**.

**Scheme 1:**
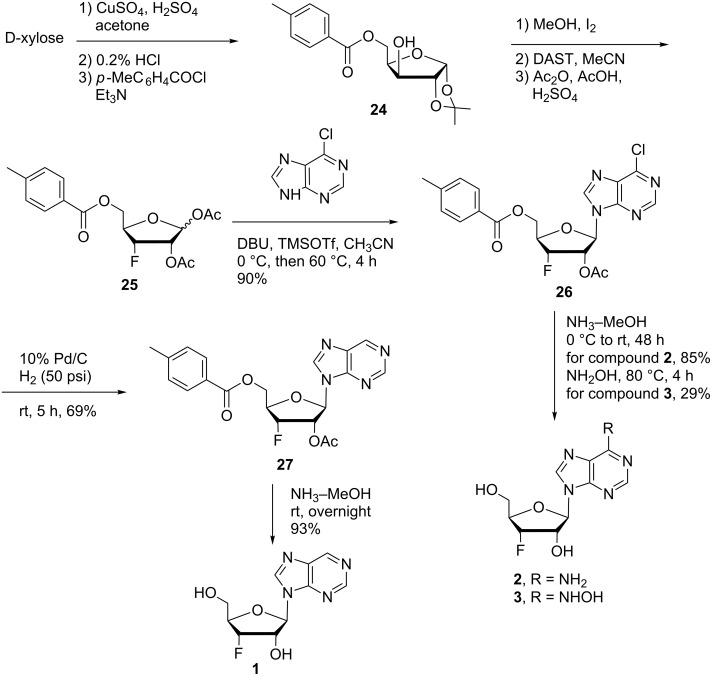
Synthesis of 3’-deoxy-3’-fluoropurine ribosides **1**–**3**.

**Scheme 2:**
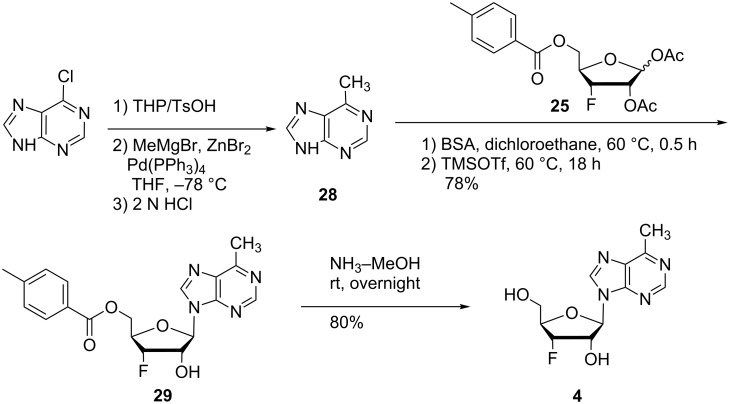
Synthesis of 6-methylpurine 3’-deoxy-3’-fluororiboside **4**.

**Scheme 3:**
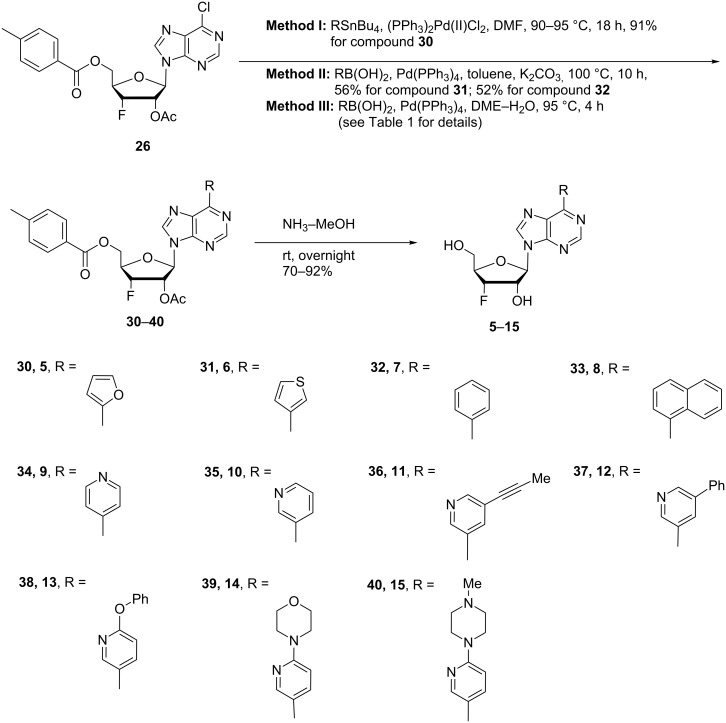
Synthesis of 6-substituted purine 3’-deoxy-3’-fluororibosides **5**–**15**.

**Scheme 4:**
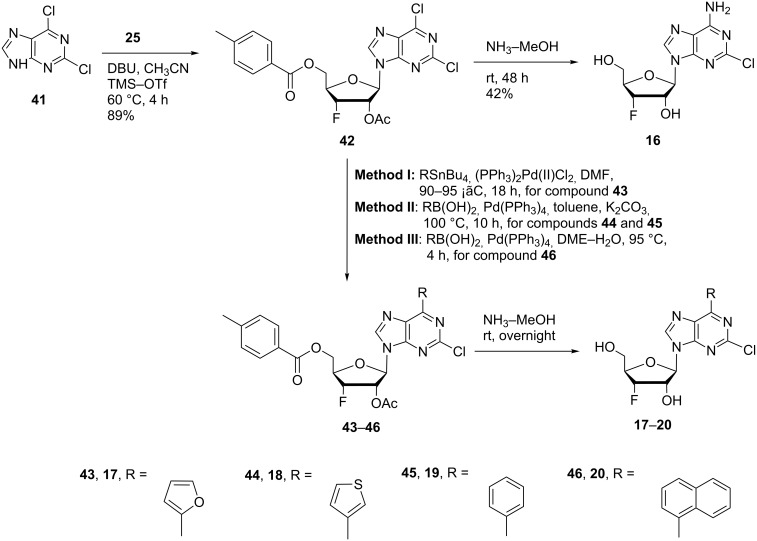
Synthesis of 6-substituted 2-chloropurine 3’-deoxy-3’-fluororibosides **16**–**20**.

**Scheme 5:**
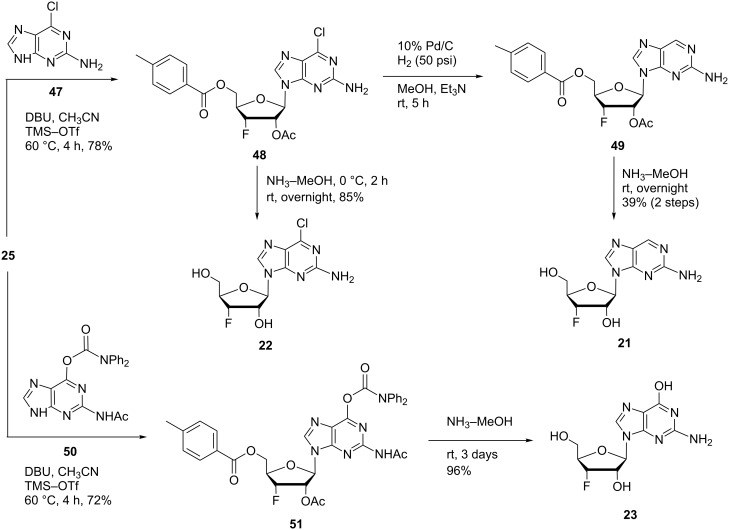
Synthesis of 2-aminopurine 3’-deoxy-3’-fluororibosides **21**–**23**.

Substituted purine 3’-deoxy-3’-fluoro-β-D-ribofuranosyl nucleosides were successfully constructed from the universal intermediate 1’,2’-di-*O*-acetyl-5’-*O*-*p*-toluyl-3’-fluoro-3’-deoxy-β-D-ribofuranose (**25**, Figure 3). The final 3’-deoxy-3’-fluoro-β-D-ribofuranose derivatives **1**–**23** (Figure 1 and Figure 2) were derivatized from the corresponding intermediates **26**, **29**, **30**–**40**, **42**, **48** and **51**. All of these compounds are novel derivatives except compounds **2** and **23**. The syntheses of these compounds are outlined in Schemes 1–5. 1,2-*O*-Isopropylidene-5-*O*-(4-methylbenzoyl)-α-D-xylofuranose (**24**) was synthesized from D-xylose according to literature procedures [41]. Compound **24** was treated with iodine in methanol, and fluorinated with diethylaminosulfur trifluoride (DAST). The universal intermediate 1’,2’-di-*O*-acetyl-5’-*O*-*p*-toluyl-3’-fluoro-3’-deoxy-β-D-ribofuranose (**25**) was then obtained in 33.13% overall yield after further treatment with acetic anhydride–acetic acid–sulfuric acid system. More than 200 g scale was achieved for the synthesis, and high purity (98%) product was obtained. This key intermediate **25** can be used to synthesize a variety of 3’-fluoro-modified nucleoside derivatives, and it was utilized for the synthesis of all analogues reported herein. This strategy avoids tedious orthogonal protecting group manipulations previously reported in literature [31–35]. Our strategy provides the desired nucleoside intermediates and also opens up the opportunity for modification on any class of nucleosides with a 3’-fluorine atom to explore their biological and therapeutic potential. While this work is related with purine nucleosides, the strategy can be used for the synthesis of a variety of nucleosides with a wide range of heterocyclic moieties to investigate the impact of a 3’-fluorine atom on the biological activity of nucleosides.

The 6-chloropurine was silylated with trimethylsilyl triflate and then glycosylated with the universal intermediate 3’-fluoro-3’-deoxy-β-D-ribofuranose **25** using 1,8-diazabicyclo[5.4.0]undec-7-ene (DBU) to provide the desired protected key intermediate **26** in 90% yield (Scheme 1). To construct the first series of fluorinated purine analogues, compound **26** was treated with a saturated solution of ammonia in methanol, which resulted in the amination at the 6-position and deprotection of the protecting groups to furnish 3’-deoxy-3’-fluoroadenosine (**2**) in 85% yield. Our synthetic strategy provided compound **2** in excellent yield (76%, 2 steps) compared to previously reported literature protocols (3.5%, 8 steps) [31–32]. The 6-chlorine of compound **26** was replaced by hydroxylamine, with concomitant removal of the protecting groups to yield *N*^6^-hydroxy-3’-fluoro-3’-deoxyadenosine (**3**). Hydrogenation of compound **26** under hydrogen pressure (50 psi) over 10% Pd/C resulted in the de-chlorinated compound **27**, which was further deprotected in a saturated solution of ammonia in methanol providing the desired 6-deaminated 3’-fluoro-adenosine **1** in 93% yield. We targeted 9-(3-deoxy-3-fluoro-β-D-ribofuranosyl)purine (**1**) in particular because it is the 3’-fluorine analogue of nebularine, a naturally occurring antibacterial and antineoplastic agent [42–43].

### 3’-Fluoro-6-methylpurine riboside **4**, a 6-β-D-MPR mimic

6-Methylpurine-β-D-riboside (6-β-D-MPR) is an isolated antibiotic agent that possesses potent antifungal, antiviral, and antitumor activities [44]. In order to explore the effect of fluorine on the biological activity of this pharmacophore, we synthesized 6-methylpurine-3’-deoxy-3’-fluoro-β-D-riboside (**4**) (Scheme 2). 6-Methylpurine (**28**) was synthesized from 6-chloropurine according to the reported protocol [44]. Compound **28** was silylated with BSA and glycosylated with the 3’-fluoro-sugar **25** to provide the desired compound **29** in 78% yield. Subsequent deprotection furnished the targeted novel fluorine modified 6-methylpurine riboside **4** in 80% yield.

### 3’-Fluoro-6-*C*-substituted purine nucleosides

The 6-*C*-substituted purine nucleosides have demonstrated anticancer, antiviral and other biological activities [11–20]. In order to explore the effect of fluorine on the biological activity of 6-*C*-substituted purine nucleosides, we designed and synthesized 3’-deoxy-3’-fluoro-β-D-ribofuranosyl purine derivatives **5**–**15** with various aromatic and heterocyclic moieties at position 6 of the purine base from the chlorine intermediate **26** (Scheme 3).

The synthesis of 3’-deoxy-3’-fluoro-β-D-ribofuranosyl purine derivatives began with a palladium-catalyzed cross coupling [45–46] to install the aromatic moieties to the 6-position of the purine ring. In order to accomplish efficient cross-couplings of a wide range of aromatic rings, we utilized three different protocols that were employed for Stille and Suzuki reaction conditions. To this end, 2-(tributylstannyl)furan was coupled with 6-chloropurine nucleoside **26** by Stille cross coupling [47–48] catalyzed by bis(triphenylphosphine)palladium(II) chloride in DMF (Method I) (Scheme 3). The resulting 6-aryl compound **30** was obtained in 91% yield and deprotected with a saturated solution of ammonia in methanol to give the desired analogue **5** with furan-2-yl substituent at position 6 of the purine base. Considering the possible toxicity of organostannyl reagents, we then utilized various organoboronic acids for Suzuki cross couplings [49–51] to synthesize compounds **6**–**15**. The 6-chloropurine intermediate **26** was coupled with 3-thienylboronic acid and phenylboronic acid catalyzed by tetrakis(triphenylphosphine)palladium (Pd(PPh_3_)_4_) in toluene (Method II), resulting in the desired protected intermediates **31** and **32** in 56% and 52% yields, respectively. Suzuki coupling efficiency in DMF (entries 2 and 3, Table 1) was not as high as Stille coupling (entry 1, Table 1). Suzuki coupling of intermediate **26** with 1-naphthylboronic acid, pyridine-4-boronic acid, pyridine-3-boronic acid and 5-propynylpyridine-3-boronic acid (Method II) (entries 4, 6, 8, and 10, Table 1) provided no detectable product. After screening various solvent systems, we discovered that dimethoxyethane (DME)–water was the optimal solvent system (Method III), and achieved construction of 6-aryl compounds **33**–**36** (entries 5, 7, 9 and 11, Table 1), albeit in low to moderate yields. Next, substituted pyridine boronic acids [52–54] were coupled with intermediate **26** using Method III in DME–water to provide the desired 6-aryl products **37**–**40** (entries 12–15, Table 1). These results indicated that the DME–water solvent system was more favourable for more challenging Suzuki C–C coupling reactions on the 6-chloropurine nucleosides. Intermediate compounds **31**–**40** were deprotected with a saturated solution of ammonia in methanol to furnish the desired products **6**–**15** in 70–92% yields (Table 1).

**Table 1:** C–C bond formation by Stille and Suzuki cross-coupling.

Entry	Method^a^	Reagents	Coupling product (yield)	Final product (yield)

1	I	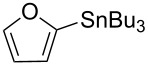	**30** (91%)	**5** (70%)
2	II	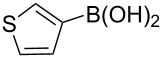	**31** (56%)	**6** (92%)
3	II	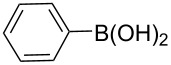	**32** (52%)	**7** (84%)
4	II	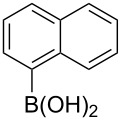	**33** (no product)	–
5	III		**33** (57%)	**8** (92%)
6	II	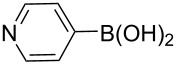	**34** (no product)	–
7	III		**34** (32%)	**9** (89%)
8	II	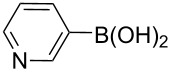	**35** (no product)	–
9	III		**35** (29%)	**10** (84%)
10	II	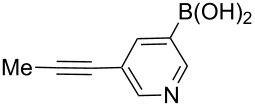	**36** (no product)	–
11	III		**36** (17%)	**11** (70%)
12	III	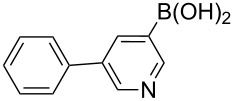	**37** (21%)	**12** (77%)
13	III	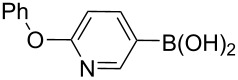	**38** (46%)	**13** (87%)
14	III	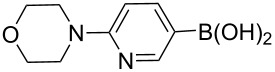	**39** (49%)	**14** (90%)
15	III	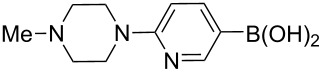	**40** (16%)	**15** (72%)
16	I	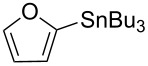	**43** (24%)	**17** (85%)
17	II	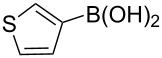	**44** (16%)	**18** (70%)
18	II	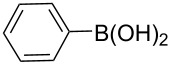	**45** (21%)	**19** (72%)
19	III	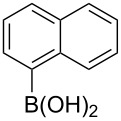	**46** (22%)	**20** (71%)

^a^Method I: (PPh_3_)_2_Pd(II)Cl_2_, DMF; Method II: Pd(PPh_3_)_4_, toluene; Method III: Pd(PPh_3_)_4_, DME–water.

### 3’-Fluoro-2-chloropurine nucleosides – cladribine and clofarabine mimics

Anticancer drugs cladribine and clofarabine [55–57] are purine nucleoside derivatives having a chlorine atom at position 2 of the purine moiety. With this in mind, we designed and synthesized 2-chloropurine nucleosides **16**–**20** (Figure 2 and Scheme 4) with 3’-fluorine and 6-*C*-aromatic or heterocyclic modifications to explore a broad biological space for this class of nucleoside derivatives. The 2,6-dichloropurine (**41**) was glycosylated with the 3’-fluororibose intermediate **25** to furnish the 2,6-dichloropurine intermediate **42** in 89% yield (Scheme 4). Chemoselective amination of the 6-position over the 2-position of the purine and deprotection of 2,6-dichloropurine **42** was achieved with a saturated solution of ammonia in methanol to furnish 2-chloro-3’-deoxy-3’-fluoroadenosine (**16**). The 2,6-dichloro-intermediate **42** was coupled with 2-(tributylstannyl)furan catalyzed by bis(triphenylphosphine)palladium(II) chloride in DMF to provide monochloro-intermediate **43** (Method I, entry 16, Table 1). Monochloro-intermediates **44** and **45** were constructed by Suzuki coupling of dichloro-intermediate **42** with 3-thienylboronic acid and phenylboronic acid in toluene (Method II; entries 17 and 18, Table 1), and monochloro-intermediate **46** was synthesized by coupling of **42** with 1-naphthylboronic acid in DME–water (Method III; entry 19, Table 1). The resulted coupling products **43–46** were deprotected with a saturated solution of ammonia in methanol to provide corresponding final products **17–20** in 70–90% yields.

Of particular importance, the 2-chlorine atom on the intermediate **42** was stable under the Stille and Suzuki reaction conditions and under ammonia deprotection conditions. However, the palladium-catalyzed cross coupling of dichloro-intermediate **42** with organostannane and organoboronic acid reagents resulted in lower yields when compared to the cross coupling of monochloro-intermediate **26**. From the amination studies of 2,6-dichloropurines, the 6-position of the purine possesses higher reactivity towards nucleophiles than the 2-position. In addition, the selectivity for the 6-position is also higher for the Stille than for the Suzuki cross coupling. The lower yields obtained from the cross-coupling of 2,6-dichloropurines is most likely contributed to the 2-chlorine reducing the reactivity of the 6-chlorine, or undesired cross-coupled products at the 2-position of the purine that may have resulted, but were not isolated.

### 3’-Fluoro-2-aminopurine nucleosides

As mentioned above, chloropurine [55–57] and deaminopurine [42–43] nucleosides have exhibited potent biological activities. We synthesized 2-amino-6-chloropurine 3’-deoxy-3’-fluororiboside **22** and 2-aminopurine-3’-deoxy-3’-fluororiboside **21** to explore the SAR of 3’-fluorine-substituted purine nucleoside derivatives (Scheme 5). The 2-amino-6-chloropurine (**47**) was glycosylated with the 3’-fluorine riboside **25** under DBU and trimethylsilyl triflate conditions. The corresponding protected intermediate nucleoside **48** was obtained in 78% yield and utilized for further derivatization. Deprotection of compound **48** with a saturated solution of ammonia in methanol at 0 °C resulted in the desired product **22** in 85% yield. In addition, the 6-chlorine atom was left untouched during the deprotection. Compound **48** was hydrogenated over 10% Pd/C under hydrogen pressure (50 psi) giving the de-chlorinated compound **49**, which was deprotected under ammonia treatment to complete the desired final product **21**. In order to further explore the SAR of this pharmacophore, we constructed 3’-deoxy-3’-fluoroguanosine (**23**, Scheme 5). *N^2^*-Acetyl-6-*O*-(diphenylcarbamoyl)guanine (**50**) was prepared according to reported protocol [58–59]. Compound **50** was silylated and glycosylated with 3’-fluorine riboside **25** providing the fully protected intermediate **51** in 72% yield. The final product **23** was obtained in 96% yield after global deprotection of **51** using a saturated solution of ammonia in methanol. This protocol of direct glycosylation for the synthesis of 3’-fluorine modified guanosine derivatives is highly advantageous compared to the previously reported methods utilizing orthogonal protecting groups, selective deprotection, and fluorination of the starting guanosine [34].

### Biological evaluation

Newly synthesized compounds were tested against the human colon cancer cell line HCT-116 and the human osteosarcoma cancer cell line 143B (Table 2). The fluorinated nebularine analog **1** and its protected analog **27** demonstrated potent tumor cell growth inhibition at sub-micromolar concentration. The protected 6-methylpurine riboside **29** exhibited better antitumor activity than its deprotected analog **4**, the 3’-fluoro-6-MPR analog. The 6-chlorine compound **22**, as well as protected purine nucleosides **32**, **39** and **40**, also showed potent inhibitory activity. 3’-Fluorine purine nucleosides **2**–**4**, **12**, **30**, and **49** showed moderate levels of inhibitory activity, but other derivatives did not show detectable activity against the tested tumor cell lines. Antiviral and other biological evaluation of these 3’-fluorine modified nucleosides is in progress and will be reported in due course.

**Table 2:** Antitumor activity of newly synthesized compounds.

Compound	Cytotoxicity IC_50_ (μM)		Compound	Cytotoxicity IC_50_ (μM)	
	HCT116	143B		HCT116	143B

**1**	0.5–1.0	0.5–1.0	**22**	0.5–1.0	0.5–1.0
**2**	1.0–5.0	1.0–5.0	**27**	0.5–1.0	0.5–1.0
**3**	5.0–10	5.0–10	**29**	0.5–1.0	0.5–1.0
**4**	5.0–10	5.0–10	**30**	1.0–5.0	1.0–5.0
**5**	>10	>10	**31**	>10	>10
**6**	>10	>10	**32**	0.5–1.0	0.5–1.0
**7**	>20	>20	**33**	>20	>30
**8**	>20	>20	**34**	>10	>10
**9**	>10	>10	**35**	>10	>10
**10**	>10	>10	**36**	>20	>20
**11**	>20	>20	**37**	>20	>20
**12**	1.0–5.0	1.0–5.0	**38**	>10	>10
**13**	>10	>10	**39**	0.5–1.0	0.5–1.0
**14**	>10	>10	**40**	0.5–5.0	0.5–5.0
**15**	>20	>20	**43**	>10	>10
**16**	>10	>10	**44**	>10	>10
**17**	>10	>10	**45**	>20	>20
**18**	>10	>10	**46**	>20	>20
**19**	>20	>20	**49**	1.0–5.0	1.0–5.0
**20**	>20	>20	Camptothecin	0.30	1.20

## Conclusion

The 6-chloropurine, 2,6-dichloropurine, and 2-amino-6-chloropurine were glycosylated with the protected 3’-deoxy-3’-fluororibose **25** to provide the corresponding key intermediates **26**, **42**, and **48**. These intermediates were then further derivatized to furnish final products **1**–**3** and **5**–**23**. The glycosylation of 6-methylpurine **28** with **25** furnished 3’-deoxy-3’-fluoro-6-methylpurine riboside **4**, the analogue of biologically active natural product, 6-methylpurine-β-D-riboside (6-β-D-MPR). We constructed 3’-deoxy-3’-fluororibofuranosylpurine nucleosides **5**–**11** with various aromatic and heterocyclic moieties at position 6 from the key intermediate **26** by Stille and Suzuki cross-coupling reactions. 3’-Deoxy-3’-fluororibofuranosyl 2-chloropurine nucleosides **17**–**20** were synthesized by the similar strategy from the key intermediate **42**. DME–water was found to be an efficient solvent system for Suzuki reaction on challenging substrates. De-chlorination of the 6-chloro-intermediates **26** and **48**, followed by deprotection, resulted in 6-deaminopurine nucleosides **1** and **21**, new analogues of the naturally isolated antibacterial and antineoplastic agent 6-deaminoadenosine, nebularine. Newly synthesized compounds were evaluated for their antitumor activity. Eleven compounds exhibited potent tumour cell growth inhibition activity against the human colon tumor cell line HCT116 and the human osteosarcoma cell line 143B at sub- or low micromolar concentration.

## Supporting Information

File 1 Experimental procedures, characterization data, and ^1^H NMR and mass spectral data for new compounds.
