# Supplementary material for: Versatile synthesis and biological evaluation of novel 3’-fluorinated purine nucleosides
Source: Beilstein J Org Chem. 2015 Dec 9;11:2509–20. doi: 10.3762/bjoc.11.272 (PMC4685831; doi:10.3762/bjoc.11.272)

Supporting Information

for

Versatile synthesis and biological evaluation of novel 3’-fluorinated purine nucleosides

Hang Ren[1,2], Haoyun An*[2], Paul J. Hatala[2], William C. Stevens, Jr.[2], Jingchao Tao*[1], Baicheng He[3]

Address: [1]College of Chemistry and Molecular Engineering, Zhengzhou University, 100 Science Avenue, Zhengzhou, China 450001; [2]Granlen, Inc., 7864 Paseo Tulipero, Carlsbad, CA 92009, USA and [3]Molecular Oncology Laboratory, The University of Chicago Medical Center, Chicago, Illinois 60637, USA

Email: Haoyun An - han@granlen.com

*Corresponding author

Experimental procedures, characterization data, and ^{1}H NMR and mass spectral data for new Compounds

Table of contents

Experimental procedures of compound **25** and products **1**–**23**……….………..….......…S3

^{1}H NMR spectrum of compound **25**………………………………………………………….…..S24

Mass spectrum of compound **25**…….……………………………..…………………….…S25

^{1}H NMR spectrum of product **1**…………………………………….……………………S26

HRMS spectrum of product **1**…………………………………….…………………....…S27

^{1}H NMR spectrum of product **2**………………………..………………………..…………..S28

HRMS spectrum of product **2**…………………………………………………..……......S29

HRMS spectrum of product **3**……………………………………………………………..…..…..S30
^{1}H NMR spectrum of product **4**…………………………………………………………...…S31
HRMS spectrum of product **4**…………………………………………………………......….S32
^{1}H NMR spectrum of product **5**………………………………………………..……………S33
^{1}H NMR spectrum of product **6**……………………………………………...…..….....S34
HRMS spectrum of product **6**…………………………………………………...…..……..S35
^{1}H NMR spectrum of product **7**…………………..……………………………………...…..…S36
HRMS spectrum of product **7**……………………………………………………………..……S37
^{1}H NMR spectrum of product **8**……………………………………………….……...…...S38
HRMS spectrum of product **8**……………………………………………….……….….....S39
^{1}H NMR spectrum of product **9**…………………..……………………………………....…S40
HRMS spectrum of product **9**………………………………………………………………...S41
^{1}H NMR spectrum of product **10**……………………………………………...………..S42
HRMS spectrum of product **10**…………………………………………...……………..S43
^{1}H NMR spectrum of product **11**…………………..……………………………………………S44
HRMS spectrum of product **11**………………………………………………………………S45
^{1}H NMR spectrum of product **12**……………………………………………...………..S46
HRMS spectrum of product **12**…………………………………………...……………..S47
^{1}H NMR spectrum of product **13**…………………..………………………………………....S48
HRMS spectrum of product **13**…………………………………………………………... S49
^{1}H NMR spectrum of product **14**……………………………………………...………..S50
HRMS spectrum of product **14**…………………………………………...……………..S51
^{1}H NMR spectrum of product **15**…………………..………………………………….....…S52
HRMS spectrum of product **15**……………………………………………………………...……S53
^{1}H NMR spectrum of product **16**……………………………………………...………..S54
HRMS spectrum of product **16**…………………………………………...……………..S55
^{1}H NMR spectrum of product **17**…………………..………………………………………...S56
HRMS spectrum of product **17**…………………………………………………………………S57

^{1}H NMR spectrum of product **18**……………………………………………………….S58
HRMS spectrum of product **18**……………………………………………………….S59
HRMS spectrum of product **19**……………………………………………………….S60
HRMS spectrum of product **20**……………………………………………………….S61
^{1}H NMR spectrum of product **21**……………………………………………………….S62
HRMS spectrum of product **21**……………………………………………………….S63
^{1}H NMR spectrum of product **22**……………………………………………………….S64
HRMS spectrum of product **22**……………………………………………………….S65
^{1}H NMR spectrum of product **23**……………………………………………………….S66
HRMS spectrum of product **23**……………………………………………………….S67
^{1}H NMR spectrum of product **26**……………………………………………………….S68

Experimental procedures of compound 25 and products 1–23

General remarks: Starting materials and reagents were obtained from commercial suppliers and were used without further purification unless otherwise stated. ^{1}H NMR spectra were obtained with a Bruker Avance 400 spectrometer using DMSO-d_6 or $CDCl_3$ as solvents. Chemical shifts are reported as δ (ppm) downfield with respect to an internal standard of tetramethylsilane (TMS). High-resolution mass spectra were obtained on a Micro-Q–TOF mass spectrometer. Product purity was tested by an Agilent 1260 analytical HPLC system. LC–MS spectra were measured on an Agilent 6120 LC–MS spectrometer. TLC was performed on silica gel GF254. Flash chromatography was performed on silica gel 200–300 mesh (Yantai Silica Gel Co. LTD). The synthesis of 1,2-*O*-isopropylidine-5-*O*-(4-methylbenzoyl)-α-D-xylofuranose (**24**) was performed with D-xylose utilizing literature procedures [1]. The synthesis of 6-methylpurine (**28**) was accomplished from 6-chloropurine according to the reported protocol [2]. The synthesis of 5-(propyn-1-

yl)pyridine-3-boronic acid, 5-phenylpyridine-3-boronic acid, 6-phenoxypyridine-3-boronic acid, 6-(4-morpholinyl)pyridine-3-boronic acid and 6-(4-methylpiperazinyl)pyridine-3-boronic acid was accomplished from their corresponding bromides according to the reported protocols [3–5]. N^2-Acety-6-*O*-(diphenylcarbamoyl)guanine (**50**) was prepared following literature procedures [6,7].

Synthesis of 1',2'-di-*O*-acetyl-5'-*O*-*p*-toluyl-3'-fluoro-3'-deoxy-β-D-ribofuranose (25). 1,2-*O*-Isopropylidene-5-*O*-(4-methylbenzoyl)-α-D-xylofuranose (**24**) (552.0 g, 1.79 mol) was dissolved in 2700 mL of methanol, and iodine (69.0 g, 0.27 mol) was added. The resulted reaction mixture was stirred at room temperature overnight, and treated with saturated sodium thiosulfate solution. The reaction mixture was concentrated under reduced pressure and purified by flash chromatography on a silica gel column using petroleum ether–ethyl acetate (5:1 and 3:1) as eluents providing 290 g (1.03 mol) of pale yellow sticky product. It was dissolved in 2500 mL of acetonitrile, and diethylaminosulfur trifluoride (DAST) (410 mL, 500 g, 3.10 mol) was added at 0 °C. The reaction mixture was stirred at room temperature overnight and quenched with water. The solution was concentrated under reduced pressure to remove acetonitrile, and then extracted with dichloromethane. The organic phase was dried over anhydrous sodium sulfate and concentrated under reduced pressure. The residue was purified by flash chromatography on a silica gel column using petroleum ether–ethyl acetate (10:1 and 5:1) as eluents providing 180 g (0.63 mol) of pay yellow sticky product. This material was dissolved in 250 mL of acetic acid and 500 mL of acetic anhydride, and 3.0 mL of concentrated sulfuric acid was added under stirring. The reaction mixture was stirred at room temperature for 2 h,

and treated with water. It was neutralized with 20.0 g of sodium bicarbonate and extracted with dichloromethane. The organic phase was dried over anhydrous sodium sulfate and concentrated under reduced pressure. The residue was purified by flash chromatography on a silica gel column using petroleum ether–ethyl acetate (10:1) as an eluent. 210 g (0.593 mol) of white sticky product **25** was obtained in 33.13% overall yield for three steps with an HPLC purity of 98.0%. R_f = 0.60 (petroleum ether–ethyl acetate = 5:1). ^{1}H NMR (400 MHz, $CDCl_3$) δ 7.85–7.99, 7.21–7.29 (m, m, 4H, α- and β- isomers, Ar-H), 6.50, 6.24 (d, t, J_d = 6.2 Hz, J_t = 2.6 Hz, 1H, α- and β- isomers, 1'-H), 5.11–5.45 (m, 2H, α- and β- isomers, 2'-H, 3'-H), 4.43–4.70, 4.40–4.49 (m, m, 3H, α- and β- isomers, 4'-H, CH_2), 2.41 (s, 3H, α- and β- isomers, Ar-CH_3), 2.09–2.20 (m, 3H, α- and β- isomers, 1'-OAc), 1.95 (s, 3H, α- and β- isomers, 2'-OAc); MS (ESI) *m/z* 376.8 $[M + Na]^+$, 730.7 $[2M + Na]^+$.

General procedure for the glycosylation of 6-chloropurine, 2,6-dichloropurine (41), 2-amino-6-chloropurine (47) and *N*^{2}-acety-6-*O*-(diphenylcarbamoyl)guanine (50) with 1',2'-di-*O*-acetyl-5'-*O*-*p*-toluyl-3'-fluoro-3'-deoxy-β-D-ribofuranose (25) (Schemes 1, 4, 5): A solution of 1',2'-di-*O*-acetyl-5'-*O*-*p*-toluyl-3'-fluoro-3'-deoxy-β-D-ribofuranose (**25**) (1.78 g, 5.0 mmol) and purine bases 6-chloropurine, **41**, **47** or **50** (5.5 mmol, 1.1 equiv) in anhydrous acetonitrile (30 mL) was cooled to 0 °C. 1,8-Diazabicyclo[5.4.0]undec-7-ene (DBU) (2.24 mL, 2.28 g, 15.0 mmol, 3.0 equiv) in anhydrous acetonitrile (30 mL) was added dropwise, followed by addition of trimethylsilyl triflate (3.62 mL, 4.45 g, 20.0 mmol, 4.0 equiv). The reaction mixture was then stirred at 60 °C for 4 h. Upon the completion of the reaction as monitored by TLC, the reaction mixture was poured into ice water and was treated with saturated sodium bicarbonate and ethyl acetate. The organic phase was

separated, and the aqueous phase was extracted with ethyl acetate. The combined organic phases were dried over anhydrous sodium sulfate. The drying agent was filtered off, and the filtrate was concentrated under reduced pressure. The residue was purified by flash chromatography on a silica gel column using petroleum ether–ethyl acetate (10:1 to 3:1) as eluents providing products **26**, **42**, **48** or **51** in 88–90% yields as white solids.

Experimental details for 9-(2-*O*-acetyl-5-*O*-*p*-toluyl-3-deoxy-3-fluoro-β-D-ribofuranosyl)-6-chloropurine (26) (Scheme 1): 2.02 g white solid, 90% yield; R_f = 0.2 (petroleum ether–ethyl acetate = 3:1). ^{1}H NMR (400 MHz, $CDCl_3$) δ 8.47 (s, 1H, Ar-H), 8.18 (s, 1H, Ar-H), 7.94 (d, *J* = 8.0 Hz, 2H, Ar-H), 7.26 (d, *J* = 6.8 Hz, 2H, Ar-H), 6.28 (d, *J* = 7.2 Hz, 1H, 1'-H), 6.11–6.20 (m, 1H, 2'-OH), 5.54–5.72 (m, 1H, 5'-OH), 4.70–4.84 (m, 2H, 2',3'-H), 4.52 (dd, J_1 = 8.8 Hz, J_2 = 3.2 Hz, 1H), 2.42 (s, 3H, CH_3), 2.13 (s, 3H, CH_3); MS (ESI) *m/z* 448.7 $[M]^+$, 471.7 $[M + Na]^+$.

Experimental details for 9-(2-*O*-acetyl-5-*O*-*p*-toluyl-3-deoxy-3-fluoro-β-D-ribofuranosyl)-2,6-dichloropurine (42) (Scheme 4): To a precooled (0 °C) mixture of 1',2'-di-*O*-acetyl-5'-*O*-*p*-toluyl-3'-fluoro-3'-deoxy-β-D-ribofuranose (**25**) (1.78 g, 5.0 mmol) and 2,6-dichloropurine (**41**) (1.04 g, 5.50 mmol, 1.1 equiv) in anhydrous acetonitrile (30 mL) was added dropwise a solution of 1,8-diazabicyclo[5.4.0]undec-7-ene (DBU) (2.24 mL, 2.28 g, 15.0 mmol, 3.0 equiv) in anhydrous acetonitrile (30 mL), followed by addition of trimethylsilyl triflate (3.62 mL, 4.45 g, 20.0 mmol, 4.0 equiv). The reaction mixture was then stirred at 60 °C for 4 h. Upon completion of the reaction as monitored by TLC, the reaction mixture was poured into ice water and was treated with saturated sodium bicarbonate and

ethyl acetate. The organic phase was separated, and the aqueous phase was extracted with ethyl acetate. The combined organic phases were dried over anhydrous sodium sulfate. The drying agent was filtered off, and the filtrate was concentrated under reduced pressure. The residue was purified by flash chromatography on a silica gel column using petroleum ether–ethyl acetate (5:1 to 3:1) as eluents providing product **42** (2.15 g, 89%) as a white solid.

Experimental details for 9-(2-*O*-acetyl-5-*O*-*p*-toluyl-3-deoxy-3-fluoro-β-D-ribofuranosyl)-2-amino-6-chloropurine (48) (Scheme 5): To a precooled (0 °C) mixture of **25** (1.78 g, 5.0 mmol) and 2-amino-6-chloropurine (**47**) (0.93 g, 5.5 mmol, 1.1 equiv) in anhydrous acetonitrile (30 mL) was added dropwise a solution of 1,8-diazabicyclo[5.4.0]undec-7-ene (DBU) (2.24 mL, 2.28 g, 15.0 mmol, 3.0 equiv) in anhydrous acetonitrile (30 mL), followed by addition of trimethylsilyl triflate (3.62 mL, 4.45 g, 20.0 mmol, 4.0 equiv). The reaction mixture was then stirred for at 60 °C for 4 h. Upon completion of the reaction as monitored by TLC, the reaction mixture was poured into ice water and was treated with saturated sodium bicarbonate and ethyl acetate. The organic phase was separated, and the aqueous phase was extracted with ethyl acetate. The combined organic phases were dried over anhydrous sodium sulfate. The drying agent was filtered off, and the filtrate was concentrated under reduced pressure. The residue was purified by flash chromatography on a silica gel column using petroleum ether–ethyl acetate (10:1 to 5:1) as eluents providing product **48** (1.80 g, 78%) as a white solid. MS (ESI) *m/z* 463.9 $[M]^+$.

Synthesis of 9-(2-*O*-acetyl-5-*O*-*p*-toluyl-3-deoxy-3-fluoro-β-D-ribofuranosyl)-N^2-acetyl-2-amino-6-*O*-(diphenylcarbamoyl)guanine (51) and 3'-deoxy-3'-fluoroguanosine (23) (Scheme 5): A solution of 1',2'-di-*O*-acetyl-5'-*O*-*p*-toluyl-3'-fluoro-3'-deoxy-β-D-ribofuranose (**25**) (2.67 g, 7.5 mmol) and N^2-acetyl-6-*O*-(diphenylcarbamoyl)guanine (**50**) (3.20 g, 8.25 mmol, 1.1 equiv) in anhydrous acetonitrile (30 mL) was cooled to 0 °C. 1,8-Diazabicyclo[5.4.0]undec-7-ene (DBU) (3.36 mL, 3.42 g, 22.5 mmol, 3.0 equiv) in anhydrous acetonitrile (20 mL) was added dropwise, followed by addition of trimethylsilyl triflate (5.43 mL, 6.67 g, 30.0 mmol, 4.0 equiv). The reaction mixture was then stirred at 60 °C for 4 h. Upon completion of the reaction as monitored by TLC, the reaction mixture was poured into ice water and was treated with saturated sodium bicarbonate and ethyl acetate. The organic phase was separated, and the aqueous phase was extracted with ethyl acetate. The combined organic phases were dried over anhydrous sodium sulfate. The drying agent was filtered off, and the filtrate was concentrated under reduced pressure. The residue was purified by flash chromatography on a silica gel column using petroleum ether–ethyl acetate (5:1 to 2:1) as eluents providing product **51** (3.69 g, 72%) as a white solid. Thus obtained compound **51** (1.0 g, 1.5 mmol) was dissolved in NH_3–MeOH (15 mL), and the reaction mixture was allowed to stir at room temperature for 3 days. Upon completion of the reaction as monitored by TLC, the solution was concentrated in vacuum, and the residue was purified on a silica gel column resulting in compound **23** (0.40 g, 96%) as a white solid with an HPLC purity of 95.1%. R_f = 0.2 (dichloromethane–methanol = 1:5). ^{1}H NMR (400 MHz, DMSO-d_6) δ 10.54 (bs, 1H, NH), 7.94 (s, 1H, Ar-H), 6.48 (bs, 2H, NH_2), 5.89 (d, *J* = 6.0 Hz, 1H, 1'-H), 5.73 (d, *J* = 8.0 Hz, 1H, 2'-OH), 5.24 (s, 1H, 5'-OH), 5.02 (dd, J_1 = 51.6 Hz, J_2 = 2.8 Hz, 1H, 3'-H), 4.66–4.82

(m, 1H, 2'-H), 4.12–4.28 (m, 1H, 4'-H), 3.59 (s, 2H, CH_2); MS (ESI) *m/z* 285 $[M]^+$, 286 $[M + H]^+$, 308 $[M + Na]^+$; HRMS (EI) *m/z* 308.0768 $[M + Na]^+$; Calcd for $C_{10}H_{12}FN_5O_4$: 308.0771 $[M + Na]^+$.

Synthesis of 9-(2-*O*-acetyl-5-*O*-*p*-toluyl-3-deoxy-3-fluoro-β-D-ribofuranosyl)purine (27) and 9-(3-deoxy-3-fluoro-β-D-ribofuranosyl)purine (1) (Scheme 1): 9-(2-*O*-Acetyl-5-*O*-*p*-toluyl-3-deoxy-3-fluoro-β-D-ribofuranosyl)-6-chloropurine (**26**) (800 mg, 1.78 mmol) was dissolved in a mixture of anhydrous methanol (30 mL), tetrahydrofuran (1.0 mL), and triethylamine (1.0 mL, 7.17 mmol). Catalyst 10% Pd/C (160 mg, 50% w/w) was added, and the mixture was stirred for 5 h under H_2 (50 Psi) atmosphere. Upon completion of the reaction as monitored by TLC, the catalyst was filtered off through Celite. The solvent was evaporated, and the crude product was purified by flash chromatography on a silica gel column using ethyl acetate as eluent giving compound **27** (506 mg, 69%) as a white solid. Compound **27** (438 mg, 1.06 mmol) was dissolved in NH_3–MeOH (10 mL). The mixture was allowed to stir at room temperature overnight and was then concentrated in vacuum. The residue was purified on a silica gel column to give compound **1** (250 mg, 93%) as a white solid with an HPLC purity of 99.5%; R_f = 0.20 (dichloromethane–methanol = 10:1). ^{1}H NMR (400 MHz, DMSO-d_6) δ 9.22 (s, 1H, Ar-H), 8.97 (s, 1H, Ar-H), 8.86 (s, 1H, Ar-H), 6.08 (d, J = 10.4 Hz, 1H, 1'-H), 5.98 (d, J = 8.8 Hz, 1H, 2'-OH), 5.30 (t, J = 7.6 Hz, 1H, 5'-OH), 5.12 (dd, J_1 = 66.8 Hz, J_2 = 5.4 Hz, 1H, 3'-H), 4.90–5.06 (m, 1H, 2'-H), 4.22–4.36 (m, 1H, 4'-H), 3.58–3.68 (m, 2H, CH_2); MS (ESI) *m/z* 254.9 $[M + H]^+$, 276.9 $[M + Na]^+$; HRMS (EI) *m/z* 255.0894 $[M + H]^+$; Calcd for $C_{10}H_{11}FN_4O_3$: 255.0815 $[M + H]^+$.

Synthesis of 3'-deoxy-3'-fluoroadenosine (2) (Scheme 1): 9-(2-*O*-Acetyl-5-*O*-*p*-toluyl-3-deoxy-3-fluoro-β-D-ribofuranosyl)-6-chloropurine (**26**) (500 mg, 1.11 mmol) was dissolved in methanol (20 mL), and the solution was saturated with dry ammonia gas at 0 °C. The reaction mixture was allowed to stand for 48 h, and was concentrated under reduced pressure. The resulting residue was purified by flash chromatography on a silica gel column using dichloromethane–methanol (20:1 to 15:1) giving 254 mg (85%) of compound **2** as a white solid with an HPLC purity of 95.0%. R_f = 0.20 (dichloromethane–methanol = 8:1). ^{1}H NMR (400 MHz, DMSO-d_6) δ 8.36 (s, 1H, Ar-H), 8.14 (s, 1H, Ar-H), 7.43 (s, 2H, NH_2), 5.89–5.95 (m, 2H, 1' -H, 2'-OH), 5.87 (s, 1H, 5'-OH), 5.08 (dd, J_1 = 67.2 Hz, J_2 = 5.4 Hz, 1H, 3'-H), 4.80–4.98 (m, 1H, 2'-H), 4.20–4.40 (m, 1H, 4'-H), 3.46–3.65 (m, 2H, CH_2); MS (ESI) *m/z* 270 $[M + H]^+$, 292 $[M + Na]^+$; HRMS (EI) *m/z* 270.0998 $[M + H]^+$; Calcd for $C_{10}H_{13}FN_5O_3$: 270.1002 $[M + H]^+$.

Synthesis of *N*^{6}-hydroxy-3'-deoxy-3'-fluoroadenosine (3) (Scheme 1): 9-(2-*O*-Acetyl-5-*O*-*p*-toluyl-3-deoxy-3-fluoro-β-D-ribofuranosyl)-6-chloropurine (**26**) (500 mg, 1.11 mmol) was dissolved in 50 % hydroxylamine aqueous solution (13 mL), and the resulted reaction mixture was then stirred at 80 °C for 4 h. Upon completion of the reaction as monitored by TLC, the reaction mixture was concentrated under reduced pressure. The residue was dissolved in NH_3–MeOH (10 mL). The mixture was allowed to stir at room temperature overnight and concentrated in vacuum. The residue was purified on a silica gel column giving compound **3** (92 mg, total yield 29%) as a white solid with an HPLC purity of 99.8%. MS (ESI) *m/z* 286 $[M + H]^+$, 308 $[M + Na]^+$; HRMS (EI) *m/z* 286.0947 $[M + H]^+$; Calcd for $C_{10}H_{13}FN_5O_4$: 286.0952 $[M + H]^+$.

Synthesis of 9-(2-*O*-acetyl-3-deoxy-3-fluoro-5-*O*-*p*-toluyl-β-D-ribofuranosyl)-6-methy-purine (29) and 9-(3-deoxy-3-fluoro-β-D-ribofuranosyl)-6-methylpurine (4) (Scheme 2): A dry N_2-flushed flask equipped with a magnetic stirrer and a septum was charged with 6-methylpurine (**28**) (2.68 g, 20.0 mmol), anhydrous 1,2-dichloroethane (40 mL), and *N,O*-bis(trimethylsilyl)acetamide (BSA) (3.7 mL, 3.10 g, 15.0 mmol, 0.75 equiv). The reaction mixture was heated to 60 °C for 30 minutes and then cooled to 20 °C. Compound **25** (7.80 g, 22.0 mmol, 1.1 equiv) and trimethylsilyl triflate (5.5 mL, 6.67 g, 30.0 mmol, 1.5 equiv) were added slowly. The mixture was stirred at 60 °C for 18 h, and then cooled to 20 °C. The reaction mixture was treated with saturated sodium bicarbonate and dichloromethane. The organic phase was separated, and the aqueous phase was extracted with dichloromethane. The combined organic phases were dried over anhydrous sodium sulfate, filtered, and concentrated in vacuum providing compound **29** (6.65 g, 78%). Compound **29** (6.65 g, 17.2 mmol) was treated with saturated ammonia methanol solution (500 mL) at room temperature overnight, and concentrated under reduced pressure. The residue was then purified by flash chromatography on a silica gel column using dichloromethane–methanol (20:1 to 10:1) as eluent providing 3.66 g (80%) of product **4** as a white solid with an HPLC purity of 98.8%; R_f = 0.40 (dichloromethane–methanol = 10:1). ^{1}H NMR (400 MHz, DMSO-d_6) δ 8.79 (s, 1H, Ar-H), 8.76 (s, 1H, Ar-H), 6.05 (d, J = 10.4 Hz, 1H, 1'-H), 5.96 (d, J = 8.8 Hz, 1H, 2'-OH), 5.33 (t, J = 7.6 Hz, 1H, 5'-OH), 5.10 (dd, J_1 = 67.2 Hz, J_2 = 5.4 Hz, 1H, 3'-H), 4.81–4.98 (m, 1H, 2'-H), 4.13–4.39 (m, 1H, 4'-H), 3.52–3.63 (m, 2H, CH_2), 2.73 (s, 3H, CH_3); MS (ESI) *m/z* 269.1 $[M + H]^+$, 291.1 $[M + Na]^+$; HRMS (EI) *m/z* 269.1046 $[M + H]^+$; Calcd for $C_{11}H_{14}FN_4O_3$: 269.1050 $[M + H]^+$.

Synthesis of 9-(2-*O*-acetyl-5-*O*-*p*-toluyl-3-deoxy-3-fluoro-β-D-ribofuranosyl)-6-(furan-2-yl)purine (30) and 9-(3-deoxy-3-fluoro-β-D-ribofuranosyl)-6-(furan-2-yl)purine (5) (Method I, Scheme 3): To a stirred mixture of 9-(2-*O*-acetyl-5-*O*-*p*-toluyl-3-deoxy-3-fluoro-β-D-ribofuranosyl)-6-chloropurine (**26**) (565 mg, 1.26 mmol) and bis(triphenylphosphine)-palladium (II) chloride (44 mg, 0.0625 mmol, 0.05 equiv) in 15 mL of anhydrous DMF was added 2-(tributylstannyl)furan (1.60 g, 4.5 mmol, 3.6 equiv) under an nitrogen atmosphere. The reaction mixture was stirred at 90–95 °C for 18 h and concentrated to dryness under reduced pressure. The residue was purified by flash chromatography on a silica gel column using petroleum ether–ethyl acetate (5:1 to 3:1 to 1:1) as eluents resulting in 550 mg of pale yellow solid product **30** in 91% yield. The resulting compound **30** (520 mg, 1.08 mmol) was treated with a saturated ammonia solution in methanol at room temperature for 18 h until completion as monitored by TLC. The reaction mixture was concentrated, and the residue was purified by flash chromatography on a silica gel column using methylene chloride–methanol (10:0 to 10:1) resulting in 244 mg of off-white solid product **5** in 70% yield with 98.0% HPLC purity; R_f = 0.40 (dichloromethane–methanol = 50:1). ^{1}H NMR (400 MHz, DMSO-d_6) δ 8.92 (s, 1H, Ar-H), 8.89 (s, 1H, Ar-H), 8.09 (s, 1H, Ar-H), 7.87 (d, J = 4.8 Hz, 1H, Ar-H), 6.82–6.85 (m, 1H, Ar-H), 6.11 (d, J = 10.4 Hz, 1H, 1'-H), 6.01 (d, J = 8.8 Hz, 1H, 2'-OH), 5.35 (t, J = 7.6 Hz, 1H, 5'-OH), 5.14 (dd, J_1 = 66.8 Hz, J_2 = 5.2 Hz, 1H, 3'-H), 4.86–5.02 (m, 1H, 2'-H), 4.24–4.40 (m, 1H, 4'-H), 3.60–3.78 (m, 2H, CH_2); MS (ESI) *m/z* 321 $[M + H]^+$, 343 $[M + Na]^+$, 359 $[M + K]^+$.

Synthesis of 9-(2-*O*-acetyl-5-*O*-*p*-toluyl-3-deoxy-3-fluoro-β-D-ribofuranosyl)-6-(thiophen-3-yl)purine (31) and 9-(3-deoxy-3-fluoro-β-D-ribofuranosyl)-6-(thiophen-3-yl)purine (6) (Method II, Scheme 3): To a stirred mixture of 9-(2-*O*-acetyl-5-*O*-*p*-toluyl-3-deoxy-3-fluoro-β-D-ribofuranosyl)-6-chloropurine (**26**) (700 mg, 1.56 mmol), 3-thienyl boronic acid (299.4 mg, 2.34 mmol, 1.5 equiv), and potassium carbonate (323.28 mg, 2.34 mmol, 1.5 equiv) in 10 mL of toluene was added $Pd(PPh_3)_4$ (92.0 mg, 0.078 mmol, 0.05 equiv). The reaction mixture was stirred at 100 °C for 10 h and cooled to room temperature. The reaction mixture was diluted with methylene chloride (10 mL) and washed with saturated ammonium chloride solution (20 mL). The aqueous phase was extracted with methylene chloride (2×5 mL), and the combined organic layer was dried over anhydrous sodium sulfate. The drying agent was filtered off, and the filtrate was concentrated under reduced pressure. The residue was purified by flash chromatography on a silica gel column using petroleum ether–ethyl acetate (3:1 to 1:1) giving 430 mg of product **31** as a white solid in 56%. Compound **31** (400 mg, 0.81 mmol) thus obtained was treated with a saturated solution of ammonia in methanol (10 mL) at room temperature overnight. The reaction mixture was concentrated under reduced pressure. The residue was purified by flash chromatography on a silica gel column using petroleum ether–ethyl acetate (2:1 to 1:1) as eluents providing 250 mg of product **6** in 92% yield with an HPLC purity of 96.3%. R_f = 0.20 (ethyl acetate–petroleum ether = 1:1). ^{1}H NMR (400 MHz, DMSO-d_6) δ 8.93–8.96 (m, 1H, Ar-H), 8.91 (s, 1H, Ar-H), 8.88 (d, J = 10.4 Hz, 1H, Ar-H), 8.19–8.22 (m, 1H, Ar-H), 7.72–7.76 (m, 1H, Ar-H), 6.08 (d, J = 7.6 Hz, 1H, 1'-H), 5.98 (d, J = 6.4 Hz, 1H, 2'-OH), 5.33 (t, J = 5.6 Hz, 1H, 5'-OH), 5.11 (dd, J_1 = 49.6 Hz, J_2 = 4.4 Hz,

1H, 3'-H), 4.93–5.03 (m, 1H, 2'-H), 4.23–4.35 (m, 1H, 4'-H), 3.62–3.67 (m, 2H, CH_2); HRMS (EI): *m/z* 337.0763 $[M + H]^+$; calcd for $C_{14}H_{14}FN_4O_3S$, 337.0771 $[M + H]^+$.

Synthesis of 9-(2-*O*-acetyl-5-*O*-*p*-toluyl-3-deoxy-3-fluoro-β-D-ribofuranosyl)-6-[6-(4-morpholinyl)pyridin-3-yl]purine (39) and 9-(3-deoxy-3-fluoro-β-D-ribofuranosyl)-6-[6-(4-morpholinyl)pyridin-3-yl]purine (14) (Method III, Scheme 3): To a stirred mixture of 9-(2-*O*-acetyl-5-*O*-*p*-toluyl-3-deoxy-3-fluoro-β-D-ribofuranosyl)-6-chloropurine (**26**) (800 mg, 1.78 mmol), 6-(4-morpholinyl)pyridine-3-boronic acid (555 mg, 2.67 mmol, 1.5 equiv), and potassium carbonate (369 mg, 2.67 mmol, 1.5 equiv) in 24 mL of DME–water (5:1) was added $Pd(PPh_3)_4$ (103 mg, 0.089 mmol, 0.05 equiv). The reaction mixture was stirred at 95 °C for 4 h and cooled to room temperature. The reaction mixture was diluted with water and adjusted to neutral with diluted hydrochloric acid. This mixture was extracted with ethyl acetate, and the combined organic phases were dried over anhydrous sodium sulfate. The drying agent was filtered off, and the filtrate was concentrated under reduced pressure. The residue was purified by flash chromatography on a silica gel column using petroleum ether–ethyl acetate (5:1 to 2:1) providing 500 mg of product **39** in 49% yield. Compound **39** (480 mg, 0.83 mmol) was treated with a saturated solution of ammonia in methanol at room temperature overnight. Then the solution was concentrated under reduced pressure, and the residue was purified by flash chromatography on a silica gel column using dichloromethane–methanol (100:1 to 60:1) as eluents resulting in 312 mg of final product **14** as a pale yellow solid in 90% yield with an HPLC purity of 95.5%; R_f = 0.20 (petroleum ether–ethyl acetate = 1:1). ^{1}H NMR (400 MHz, DMSO-d_6) δ 9.60 (d, J = 2.4 Hz, 1H, Ar-H), 8.84–8.91 (m, 3H, Ar-H), 7.03 (d, J = 9.2 Hz, 1H, Ar-H), 6.09 (d, J = 7.6 Hz, 1H,

1’-H), 5.98 (d, J = 6.4 Hz, 1H, 2’-OH), 5.36 (t, J = 6.0 Hz, 1H, 5’-OH), 5.12 (dd, J_1 = 50.4 Hz, J_2 = 4.4 Hz, 1H, 3’-H), 4.92–5.04 (m, 1H, 2’-H), 4.25–4.36 (m, 1H, 4’-H), 3.61–3.73 (m, 10H, CH_2). HRMS (EI) *m/z* 417.1681 $[M + H]^+$; Calcd for $C_{19}H_{22}FN_6O_4$: 417.1687 $[M + H]^+$.

Experimental details for 9-(2-*O*-acetyl-5-*O*-*p*-toluyl-3-deoxy-3-fluoro-β-D-ribofuranosyl)-6-phenylpurine (32) and 9-(3-deoxy-3-fluoro-β-D-ribofuranosyl)-6-phenylpurine (7) (Scheme 3): These compounds were synthesized by Method II as described above from 1.0 g of **26** and phenylboronic acid, resulting in 564 mg of compound **32** in 52% yield. Further deprotection of compound **32** (530 mg) resulted in 300 mg of final product **7** as a white solid in 84% yield with an HPLC purity of 95.1%; R_f = 0.20 (petroleum ether–ethyl acetate = 1:1). ^{1}H NMR (400 MHz, DMSO-d_6) δ 9.00 (s, 1H, Ar-H), 8.91 (s, 1H, Ar-H), 8.76–8.82 (m, 2H, Ar-H), 7.54–7.62 (m, 3H, Ar-H), 6.11 (d, J = 8.0 Hz, 1H, 1’-H), 5.98 (d, J = 6.4 Hz, 1H, 2’-OH), 5.32 (t, J = 5.6 Hz, 1H, 5’-OH), 5.12 (dd, J_1 = 50.4 Hz, J_2 = 4.0 Hz, 1H, 3’-H), 4.93–5.02 (m, 1H, 2’-H), 4.24–4.36 (m, 1H, 4’-H), 3.62–3.70 (m, 2H, CH_2); HRMS (EI): *m/z* 331.1204 $[M + H]^+$; Calcd for $C_{16}H_{15}FN_4O$: 331.1128 $[M + H]^+$.

Experimental details of 9-(2-*O*-acetyl-5-*O*-*p*-toluyl-3-deoxy-3-fluoro-β-D-ribofuranosyl)-6-(naphthalen-1-yl)purine (33) and 9-(3-deoxy-3-fluoro-β-D-ribofuranosyl)-6-(naphtahlen-1-yl)purine (8) (Scheme 3): These compounds were synthesized by Method III as described above from 1.0 g of **26** and 1-naphthylboronic acid, resulting in 681 mg of compound **33** in 57% yield. Further deprotection of compound **33** (651 mg) resulted in 422 mg of final product **8** as a white solid in 92% yield with an HPLC

purity of 98.0%; R_f = 0.20 (dichloromethane–methanol = 10:1). ^{1}H NMR (400 MHz, DMSO-d_6) δ 9.12 (s, 1H, Ar-H), 8.86 (s, 1H, Ar-H), 8.13 (d, *J* = 10.8 Hz, 2H, Ar-H), 8.04 (d, *J* = 10.4 Hz, 1H, Ar-H), 7.95 (d, *J* = 10.0 Hz, 1H, Ar-H), 7.68 (t, *J* = 10.4 Hz, 1H, Ar-H), 7.49–7.62 (m, 2H, Ar-H), 6.16 (d, *J* = 10.4 Hz, 1H, 1'-H), 6.03 (d, *J* = 8.4 Hz, 1H, 2'-OH), 5.33 (t, *J* = 8.0 Hz, 1H, 5'-OH), 4.97–5.05 (m, 2H, 3', 2'-H), 4.26–4.40 (m, 1H, 4'-H), 3.62–3.72 (m, 2H, CH_2); HRMS (EI): *m/z* 381.1280 $[M + H]^+$; Calcd for $C_{20}H_{17}FN_4O_3$: 381.1285 $[M + H]^+$.

Experimental details of 9-(2-*O*-acetyl-5-*O*-*p*-toluyl-3-deoxy-3-fluoro-β-D-ribofuranosyl)-6-(pyridin-4-yl)purine (34) and 9-(3-deoxy-3-fluoro-β-D-ribofuranosyl)-6-(pyridin-4-yl)purine (9) (Scheme 3): These compounds were synthesized by Method III as described above from 0.60 g of **26** and pyridine-4-boronic acid, resulting in 210 mg of compound **34** in 32% yield. Further deprotection of compound **34** (200 mg) resulted in 120 mg of final product **9** as a pale yellow solid in 89% yield with an HPLC purity of 98.7%; R_f = 0.20 (dichloromethane–methanol = 10:1). ^{1}H NMR (400 MHz, DMSO-d_6) δ 9.10 (s, 1H, Ar-H), 9.00 (s, 1H, Ar-H), 8.82 (d, *J* = 5.6 Hz, 2H, Ar-H), 8.64 (d, *J* = 6.0 Hz, 2H, Ar-H), 6.13 (d, *J* = 8.0 Hz, 1H, 1'-H), 5.99 (d, *J* = 6.4 Hz, 1H, 2'-OH), 5.29 (t, *J* = 5..6 Hz, 1H, 5'-OH), 5.13 (dd, J_1 = 50.4 Hz, J_2 = 4.0 Hz, 1H, 3'-H), 4.92–5.04 (m, 1H, 2'-H), 4.24–4.36 (m, 1H, 4'-H), 3.61–3.70 (m, 2H, CH_2); HRMS (EI) *m/z* 332.1156 $[M + H]^+$; Calcd for $C_{15}H_{14}FN_5O_3$: 332.1159 $[M + H]^+$.

Experimental details of 9-(2-*O*-acetyl-5-*O*-*p*-toluyl-3-deoxy-3-fluoro-β-D-ribofuranosyl)-6-(pyridin-3-yl)purine (35) and 9-(3-deoxy-3-fluoro-β-D-ribofuranosyl)-6-(pyridin-3-yl)purine (10) (Scheme 3): These compounds were synthesized by Method

III as described above from 1.0 g of **26** and pyridine-3-boronic acid, resulting in 320 mg of compound **35** in 29% yield. Further deprotection of compound **35** (300 mg) resulted in 170 mg of final product **10** as a white solid in 84% yield; R_f = 0.20 (dichloromethane–methanol = 10:1). ^{1}H NMR (400 MHz, DMSO-d_6) δ 9.88 (d, J = 1.6 Hz, 1H, Ar-H), 9.01–9.11 (m, 2H, Ar-H), 8.96 (s, 1H, Ar-H), 8.72–8.76 (m, 1H, Ar-H), 7.60–7.67 (m, 1H, Ar-H), 6.12 (d, J = 8.0 Hz, 1H, 1'-H), 5. 99 (d, J = 6.4 Hz, 1H, 2'-OH), 5.30 (t, J = 5.6 Hz, 1H, 5'-OH), 5.15 (dd, J_1 = 50.4 Hz, J_2 = 4.0 Hz, 1H, 3'-H), 4.92–5.04 (m, 1H, 2'-H), 4.25–4.36 (m, 1H, 4'-H), 3.61–3.71 (m, 2H, CH_2); HRMS (EI) *m/z* 332.1157 [M + H]$^+$; Calcd for $C_{15}H_{15}FN_5O_3$: 332.1159 [M + H]$^+$.

Experimental details of 9-(2-*O*-acetyl-5-*O*-*p*-toluyl-3-deoxy-3-fluoro-β-D-ribofuranosyl)-6-[5-(propyn-1-yl)pyridine-3-yl]purine (36) and 9-(3-deoxy-3-fluoro-β-D-ribofuranosyl)-6-[5-(propyn-1-yl)pyridine-3-yl]purine (11) (Scheme 3): These compounds were synthesized by Method III as described above from 538 mg of **26** and 5-(propyn-1-yl)pyridine-3-boronic acid resulting in compound **36** (108 mg, 17%). Further deprotection resulted in 53 mg of final product **11** as a white solid in 70% yield with an HPLC purity of 97.0%; R_f = 0.20 (dichloromethane–methanol = 50:1). ^{1}H NMR (400 MHz, DMSO-d_6) δ 9.76 (d, J = 1.6 Hz, 1H, Ar-H), 9.05–9.10 (m, 2H, Ar-H), 9.00 (s, 1H, Ar-H), 8.76 (d, J = 2.0 Hz, 1H, Ar-H), 6.14 (d, J = 8.0 Hz, 1H, 1'-H), 6.02 (d, J = 6.4 Hz, 1H, 2'-OH), 5.32 (t, J = 5.6 Hz, 1H, 5'-OH), 5.14 (dd, J_1 = 50.4 Hz, J_2 = 4.0 Hz, 1H, 3'-H), 4.94–5.10 (m, 1H, 2'-H), 4.25–4.40 (m, 1H, 4'-H), 3.60–3.69 (m, 2H, CH_2), 2.13 (s, 3H, CH_3); HRMS (EI) *m/z* 370.1312 [M + H]$^+$; Calcd for $C_{18}H_{17}FN_5O_3$: 370.1315 [M + H]$^+$.

Experimental details of 9-(2-*O*-acetyl-5-*O*-*p*-toluyl-3-deoxy-3-fluoro-β-D-ribofuranosyl)-6-(5-phenylpyridin-3-yl)purine (37) and 9-(3-deoxy-3-fluoro-β-D-ribofuranosyl)-6-(5-phenylpyridine-3-yl)purine (12) (Scheme 3): These compounds were synthesized by Method III as described above from 1.0 g of **26** and 5-phenylpyridine-3-boronic acid resulting in compound **37** (271 mg, 21%). Further deprotection resulted in 150 mg of final product **12** as a white solid in 77% yield with an HPLC purity of 98.1%; R_f = 0.30 (dichloromethane–methanol = 10:1). ^{1}H NMR (400 MHz, DMSO-d_6) δ 9.86 (d, *J* = 2.8 Hz, 1H, Ar-H), 9.31 (t, *J* = 2.8 Hz, 1H, Ar-H), 9.11 (s, 1H, Ar-H), 9.08 (d, *J* = 3.2 Hz, 1H, Ar-H), 9.02 (s, 1H, Ar-H) , 7.77–7.87 (m, 2H, Ar-H), 7.44–7.61 (m, 3H, Ar-H), 6.16 (d, *J* = 10.4 Hz, 1H, 1'-H), 6.02 (d, *J* = 8.4 Hz, 1H, 2'-OH), 5.33 (t, *J* = 7.6 Hz, 1H, 5'-OH), 5.04–5.26 (m, 1H, 3'-H), 4.95–5.05 (m, 1H, 2'-H), 4.25–4.40 (m, 1H, 4'-H), 3.63–3.72 (m, 2H, CH_2); HRMS (EI) *m/z* 408.1469 $[M + H]^+$; Calcd for $C_{21}H_{19}FN_5O_3$: 408.1472 $[M + H]^+$.

Experimental details of 9-(2-*O*-acetyl-5-*O*-*p*-toluyl-3-deoxy-3-fluoro-β-D-ribofuranosyl)-6-(6-phenoxypyridin-3-yl)purine (38) and 9-(3-deoxy-3-fluoro-β-D-ribofuranosyl)-6-(6-phenoxypyridin-3-yl)purine (13) (Scheme 3): These compounds were synthesized by Method III as described above from 500 mg of **26** and 6-phenoxypyridine-3-boronic acid resulting in compound **38** (300 mg, 46%). Further deprotection resulted in 190 mg of final product **13** as a white solid in 87% yield; R_f = 0.10 (dichloromethane–methanol = 15:1). ^{1}H NMR (400 MHz, DMSO-d_6) δ 9.76 (s, 1H, Ar-H), 9.05 (s, 1H, Ar-H), 8.99 (s, 1H, Ar-H), 8.57–8.68 (m, 2H, Ar-H), 7.49 (t, *J* = 7.6 Hz, 2H, Ar-H), 7.16–7.33 (m, 3H, Ar-H), 6.14 (d, *J* = 8.0 Hz, 1H, 1'-H), 6. 03 (d, *J* = 6.4 Hz, 1H, 2'-OH), 5.34 (t, *J* = 5.6 Hz, 1H, 5'-OH), 5.16 (dd, J_1 = 50.4 Hz, J_2 = 4.0 Hz, 1H, 3'-H), 4.93–

5.07 (m, 1H, 2'-H), 4.28–4.42 (m, 1H, 4'-H), 3.59–3.76 (m, 2H, CH_2); HRMS (EI) *m/z* 424.1419 $[M + H]^+$; Calcd for $C_{21}H_{19}FN_5O_4$: 424.1421 $[M + H]^+$.

Experimental details of 9-(2-*O*-acetyl-5-*O*-*p*-toluyl-3-deoxy-3-fluoro-β-D-ribofuranosyl)-6-[6-(4-methylpiperazinyl)pyridin-3-yl]purine (40) and 9-(3-deoxy-3-fluoro-β-D-ribofuranosyl)-6-[6-(4-methylpiperazinyl)purine (15) (Scheme 3): These compounds were synthesized by Method III as described above from 1.0 g of **26** and 6-(4-methylpiperazinyl)pyridine-3-boronic acid resulting in compound **40** (205 mg, 16%). Further deprotection resulted in 107 mg of final product **15** as a pale yellow solid in 72% yield with an HPLC purity of 99.3%; R_f = 0.30 (dichloromethane–methanol = 8:1). ^{1}H NMR (400 MHz, DMSO-d_6) δ 9.62 (d, *J* = 2.4 Hz, 1H, Ar-H), 8.86–8.98 (m, 2H, Ar-H), 7.14 (d, *J* = 9.6 Hz, 1H, Ar-H), 6.10 (d, *J* = 8.0 Hz, 1H, 1'-H), 6.01 (d, *J* = 6.4 Hz, 1H, 2'-OH), 5.38 (t, *J* = 6.0 Hz, 1H, 5'-OH), 5.14 (dd, J_1 = 50.0 Hz, J_2 = 4.4 Hz, 1H, 3'-H), 4.94–5.04 (m, 1H, 2'-H), 4.26–4.36 (m, 1H, 4'-H), 3.62–3.69 (m, 2H, CH_2), 2.72 (s, 3H, CH_3); HRMS (EI): *m/z* 430.2000 $[M + H]^+$; calcd for $C_{20}H_{25}FN_7O_3$, 430.2003 $[M + H]^+$.

Experimental details of 2-chloro-3'-deoxy-3'-fluoroadenosine (16) (Scheme 4): Compound **16** was synthesized as described above for compound **2** from the corresponding intermediate **42** (500 mg). Flash chromatographic purification on a silica gel column using dichloromethane–methanol (20:1 to 15:1) as eluents provided 133 mg of product **16** as a white solid in 42% yield; R_f = 0.20 (dichloromethane–methanol = 10:1). ^{1}H NMR (400 MHz, DMSO-d_6) δ 8.40 (s, 1H, Ar-H), 7.92 (bs, 2H, NH_2), 5.94 (d, *J* = 6.8 Hz, 1H, 1'-H), 5.86 (d, *J* = 8.0 Hz, 1H, 2'-OH), 5.26 (t, *J* = 6.0 Hz, 1H, 5'-OH), 5.08 (dd, J_1 =

50.0 Hz, J_2 = 4.4 Hz, 1H, 3'-H), 4.78–4.93 (m, 1H, 2'-H), 4.21–4.33 (m, 1H, 4'-H), 3.59–3.68 (m, 2H, CH_2); HRMS (EI) *m/z* 304.0607 $[M + H]^+$; Calcd for $C_{10}H_{12}ClFN_5O_3$: 304.0613 $[M + H]^+$.

Experimental details of 9-(2-*O*-acetyl-5-*O*-*p*-toluyl-3-deoxy-3-fluoro-β-D-ribofuranosyl)-2-chloro-6-(furan-2-yl)purine (43) and 2-chloro-9-(3-deoxy-3-fluoro-β-D-ribofuranosyl)-6-(furan-2-yl)purine (17) (Scheme 4): These compounds were synthesized by Method I as described above for compounds **30** and **5** from key intermediate **42** (700 mg, 1.45 mmol) and 2-(tributylstannyl)furan (569.6 mg, 1.60 mmol, 1.1 equiv) resulting in compound **43** (175 mg, 24%). Further deprotection resulted in 102 mg of final product **17** as a white solid in 85% yield with an HPLC purity of 95.0%; R_f = 0.50 (dichloromethane–methanol = 50:1). ^{1}H NMR (400 MHz, DMSO-d_6) δ 8.92 (s, 1H, Ar-H), 8.15 (d, *J* = 0.8 Hz, 1H, Ar-H), 7.91 (d, *J* = 3.2 Hz, 1H, Ar-H), 6.85–6.91 (m, 1H, Ar-H), 5.99–6.07 (m, 2H, 1', 2'-OH), 5.27 (t, *J* = 5.6 Hz, 1H, 5'-OH), 5.14 (dd, J_1 = 50.0 Hz, J_2 = 4.0 Hz, 1H, 3'-H), 4.86–4.99 (m, 1H, 2'-H), 4.27–4.38 (m, 1H, 4'-H), 3.63–3.72 (m, 2H, CH_2); HRMS (EI) *m/z* 355.0616 $[M + H]^+$; Calcd for $C_{14}H_{13}ClFN_4O_4$: 355.0609 $[M + H]^+$.

Experimental details of 9-(2-*O*-acetyl-5-*O*-*p*-toluyl-3-deoxy-3-fluoro-β-D-ribofuranosyl)-2-chloro-6-(thiophen-3-yl)purine (44) and 2-chloro-9-(3-deoxy-3-fluoro-β-D-ribofuranosyl)-6-(thiophen-3-yl)purine (18) (Scheme 4): These compounds were synthesized by Method II as described above for compounds **31** and **6** from key intermediate **42** (950 mg, 1.66 mmol) and 3-thienylboronic acid (319 mg, 2.49 mmol, 1.5 equiv) resulting in compound **44** (164 mg, 16%). Further deprotection resulted in 80 mg of

final product **18** as a white solid in 70% yield with an HPLC purity of 95.0%; R_f = 0.50 (dichloromethane–methanol = 10:1). ^{1}H NMR (400 MHz, DMSO-d_6) δ 8.99 (d, J = 2.0 Hz, 1H, Ar-H), 8.95 (s, 1H, Ar-H), 8.18 (d, J = 5.2 Hz, 1H, Ar-H), 7.75–7.86 (m, 1H, Ar-H), 6.01–6.16 (m, 2H, 1', 2'-OH), 5.30 (t, J = 5.2 Hz, 1H, 5'-OH), 5.15 (dd, J_1 = 50.0 Hz, J_2 = 4.4 Hz, 1H, 3'-H), 4.87–5.01 (m, 1H, 2'-H), 4.27–4.39 (m, 1H, 4'-H), 3.60–3.76 (m, 2H, CH_2); HRMS (EI) *m/z* 371.0376 $[M + H]^+$; Calcd for $C_{14}H_{13}ClFN_4O_3S$: 371.0381 $[M + H]^+$.

Experimental details of 9-(2-*O*-acetyl-5-*O*-*p*-toluyl-3-deoxy-3-fluoro-β-D-ribofuranosyl)-2-chloro-6-phenylpurine (45) and 2-chloro-9-(3-deoxy-3-fluoro-β-D-ribofuranosyl)-6-phenylpurine (19) (Scheme 4): These compounds were synthesized by Method II as described above for compounds **31** and **6** from 500 mg of **42** and phenylboronic acid, resulting in compound **45** (113 mg, 21%). Further deprotection resulted in 57 mg of final product **19** as a white solid in 72% yield; HRMS (EI) *m/z* 365.0811 $[M + H]^+$; Calcd for $C_{16}H_{15}ClFN_4O_3$: 365.0817 $[M + H]^+$.

Experimental details of 9-(2-*O*-acetyl-5-*O*-*p*-toluyl-3-deoxy-3-fluoro-β-D-ribofuranosyl)-2-chloro-6-(naphthalen-1-yl)purine (46) and 2-chloro-9-(3-deoxy-3-fluoro-β-D-ribofuranosyl)-6-(naphthalen-1-yl)purine (20) (Scheme 4): These compounds were synthesized by Method III as described above for compounds **31** and **6** from 240 mg of **42** and naphthalene-2-boronic acid, resulting in compound **46** (61 mg, 22%). Further deprotection of compound **46** resulted in 31 mg of final product **20** as a white solid in 71% yield; HRMS (EI) *m/z* 415.0970 $[M + H]^+$; Calcd for $C_{20}H_{17}ClFN_4O_3$: 415.0973 $[M + H]^+$.

Synthesis of 9-(2-*O*-acetyl-5-*O*-*p*-toluyl-3-deoxy-3-fluoro-β-D-ribofuranosyl)-2-aminopurine (49) and 2-amino-9-(3-deoxy-3-fluoro-β-D-ribofuranosyl)purine (21) (Scheme 5): Compounds **49** and **21** were synthesized as described above for compounds **27** and **1** by hydrogenation of key intermediate **48** (1.80 g, 3.9 mmol), followed by deprotection. 400 mg of compound **21** was obtained as a white solid in 39% overall yield with an HPLC purity of 95.1%; R_f = 0.30 (dichloromethane–methanol = 10:1). ^{1}H NMR (400 MHz, DMSO-d_6) δ 8.62 (s, 1H, Ar-H), 8.31 (s, 1H, Ar-H), 6.59 (s, 2H, NH_2), 5.94 (d, J = 6.4 Hz, 1H, 1'-H), 5.88 (d, J = 8.0 Hz, 1H, 2'-OH), 5.27 (t, J = 5.6 Hz, 1H, 5'-OH), 5.07 (dd, J_1 = 50.4 Hz, J_2 = 4.4 Hz, 1H, 3'-H), 4.79–4.95 (m, 1H, 2'-H), 4.16–4.29 (m, 1H, 4'-H), 3.59–3.64 (m, 2H, CH_2); MS (ESI) *m/z* 304 $[M + H]^+$, 326.9 $[M + Na]^+$; HRMS (EI) *m/z* 270.0999 $[M + H]^+$; Calcd for $C_{10}H_{13}FN_5O_3$: 270.1002 $[M + H]^+$.

2-Amino-6-chloro-9-(3-deoxy-3-fluoro-β-D-ribofuranosyl) purine (22) (Scheme 5): Compound **22** was synthesized as described above for compound **2** from key intermediate **48** (251 mg, 0.54 mmol), and the reaction was monitored by TLC. 140 mg of product **22** was obtained as a white solid in 85% yield after flash chromatographic purification on a silica gel column using dichloromethane–methanol (50:1 to 20:1) as eluents; R_f = 0.40 (dichloromethane–methanol = 10:1); with an HPLC purity of 98.0%. ^{1}H NMR (400 MHz, DMSO-d_6) δ 8.38 (s, 1H, Ar-H), 7.01 (s, 2H, NH_2), 5.96 (d, J = 6.0 Hz, 1H, 1'-H), 5.85 (d, J = 8.0 Hz, 1H, 2'-OH), 5.24 (t, J = 5.2 Hz, 1H, 5'-OH), 5.07 (dd, J_1 = 50.4 Hz, J_2 = 4.0 Hz, 1H, 3'-H), 4.75–4.90 (m, 1H, 2'-H), 4.16–4.28 (m, 1H, 4'-H), 3.58–3.65 (m, 2H, CH_2); MS

(ESI) *m/z* 304 $[M + H]^+$, 326.9 $[M + Na]^+$; HRMS (EI) *m/z* 304.0608 $[M + H]^+$; Calcd for $C_{10}H_{12}ClFN_5O_3$: 304.0613 $[M + H]^+$.

Assay: Proliferation inhibitory effect assay: Exponentially growing HCT116 cells were seeded in 96 well plates, the initial density is 30%. The cells were treated with a serial concentrations of the synthesized compounds for 24 h. Before assay, the medium were changed with fresh medium, added the WST-8 agent in each well (10 μl per well), and then incubated for 1h. Finally, the absorbance was measured at 450 nm. Each assay was done in triplicate.

References

1. Ozols, A. M.; Azhayev, A. V.; Dyatkina, N. B.; Krayevsky, A. A. *Synthesis* **1980,** 557–559.
2. Marasco, C., Jr.; Pera P. J.; Spiess, A. J.; Bernacki, R.; Sufrin, J. R. *Molecules* **2005,** *10,* 1015–1020.
3. Parry, P. R.; Bryce, M. R.; Tarbit, B. *Synthesis* **2003**, *(7)*, 1035–1038.
4. Li, W.-J.; Nelson, D. P.; Jensen, M. S.; Hoerrner R. S.; Cai, D.-W.; Larsen, R. D.; Reider, P. J. *J. Org. Chem.* **2002**, *67*, 5394–5397.
5. Cioffi, C. L.; Spencer, W. T.; Richards, J. J.; Herr, R. J. *J. Org. Chem.* **2004**, *69*, 2210–2212.
6. Dalpozzo, R.; De Nino, A.; Maiuolo, L.; Procopio, A.; De Munno, G.; Sindona, G. *Tetrahedron* **2001**, *57*, 4035–4038.
7. Robins, M. J.; Zou, R.-M.; Guo, Z.-Q.; Wnuk, S. F. *J. Org. Chem.* **1996,** *61,* 9207–9212.

Compound 25: ^{1}H NMR (400 MHz, $CDCl_3$)

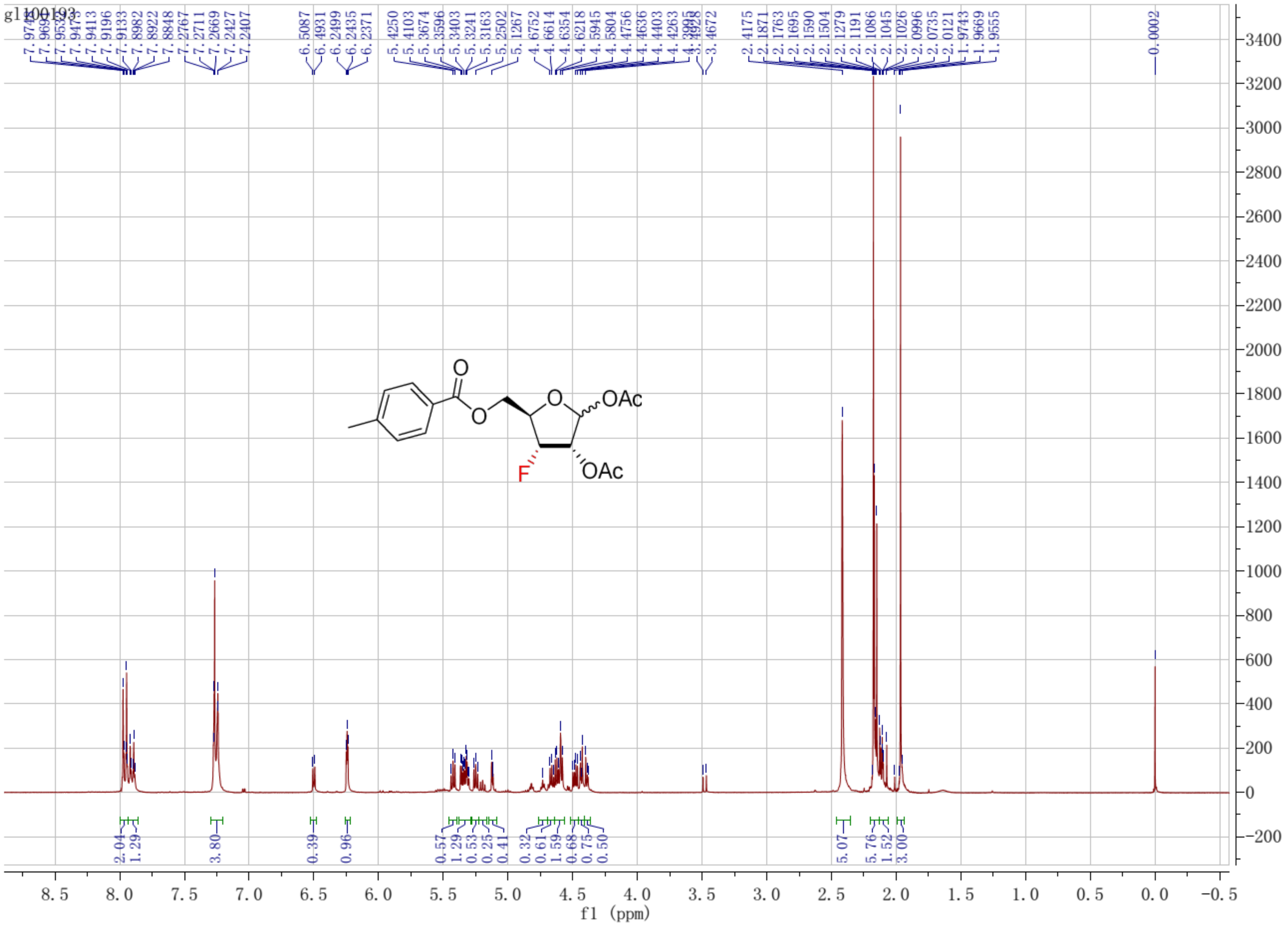

Compound 25: Mass Spectrum

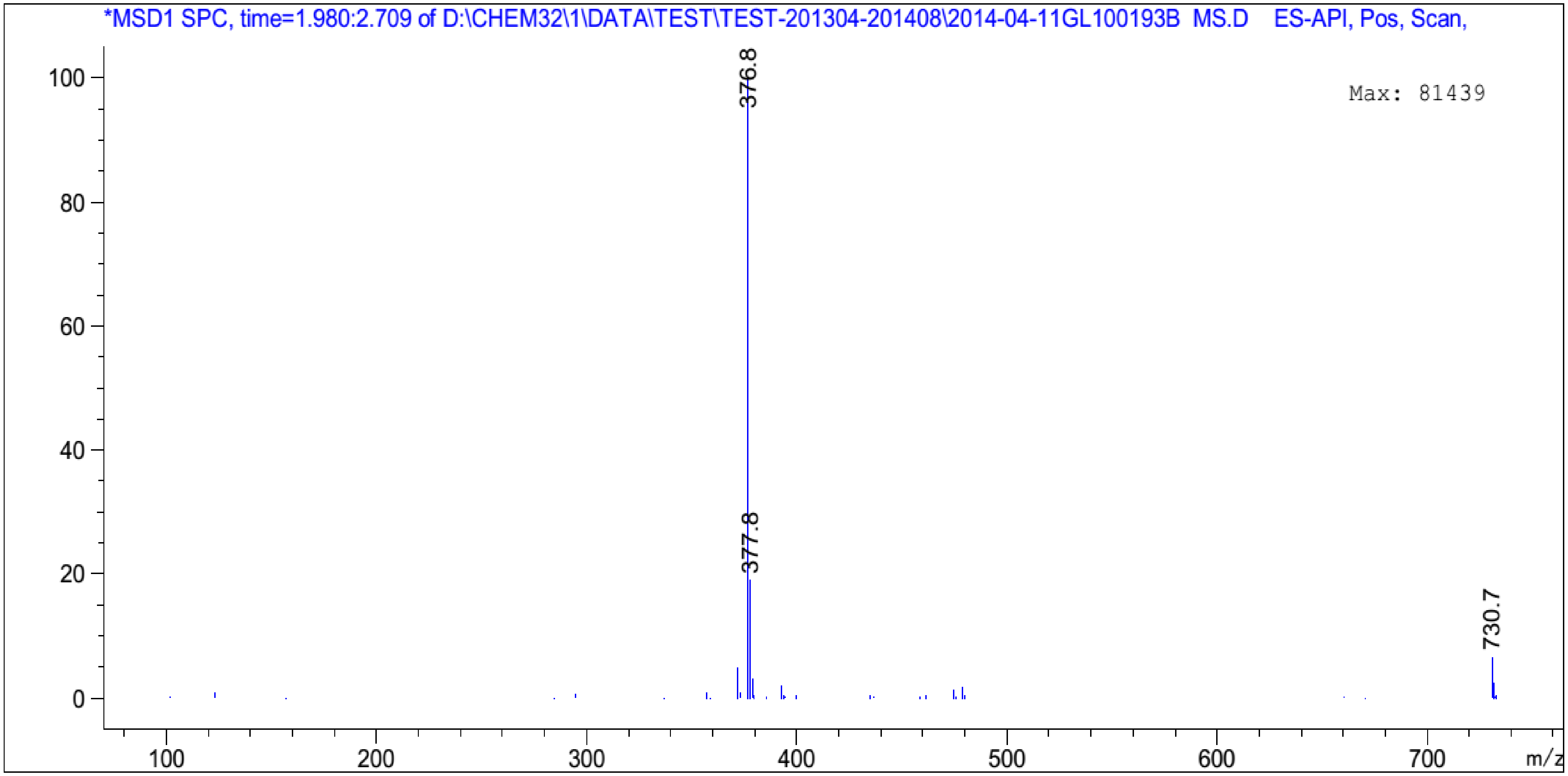

Product 1: ^1H NMR (400 MHz, DMSO-d_6)

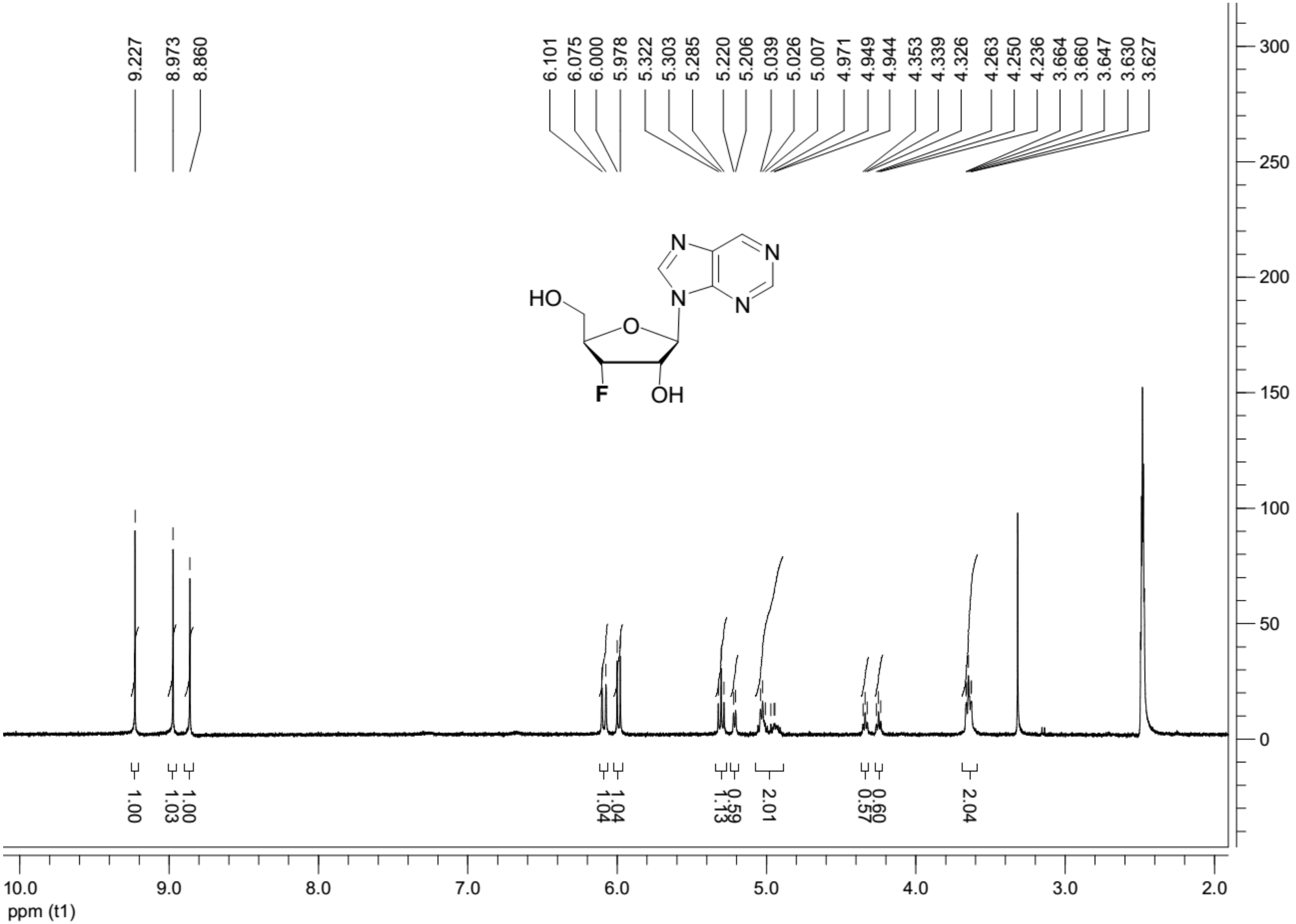

Product 1: HRMS

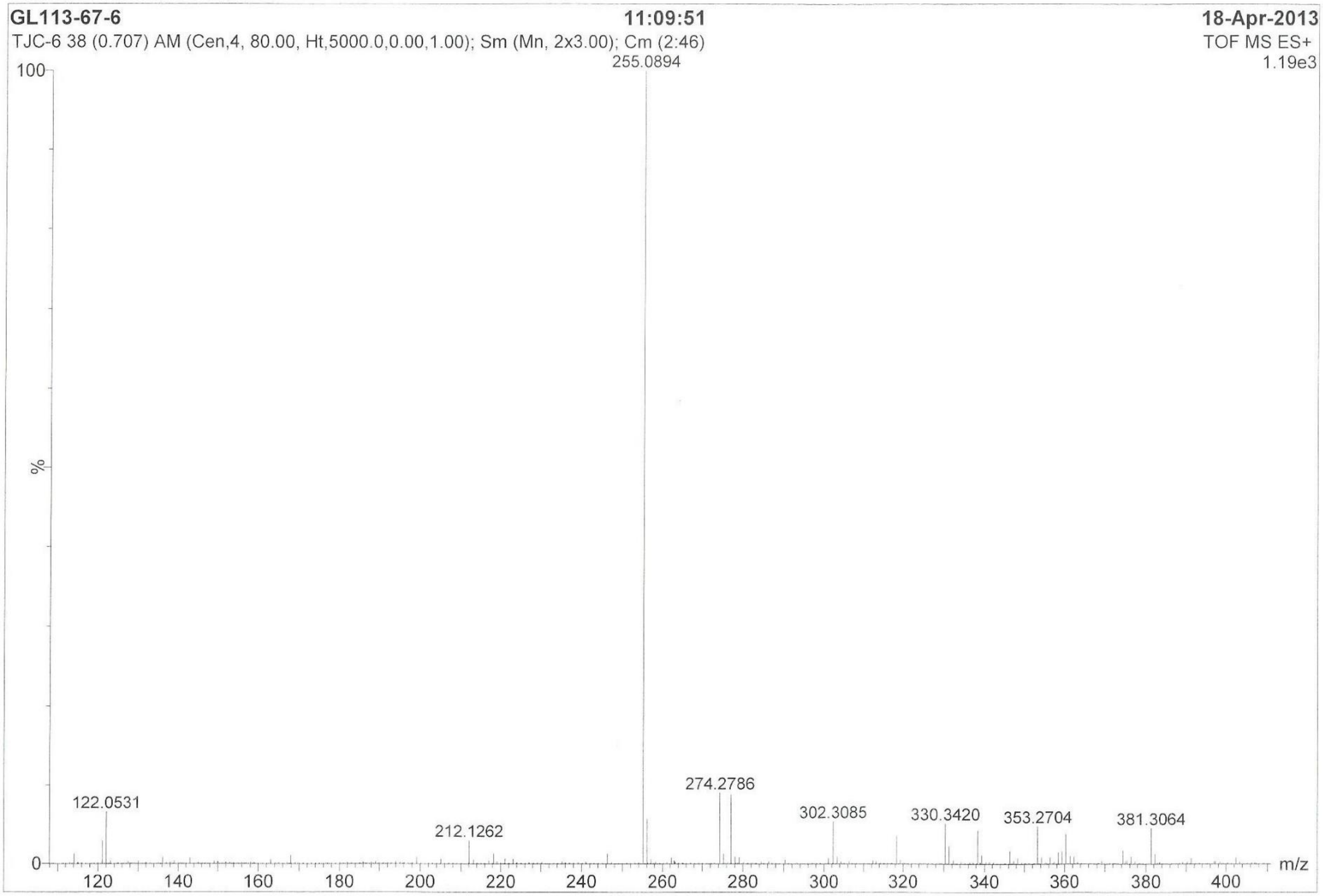

Product 2: ^{1}H NMR (400 MHz, DMSO-d_6)

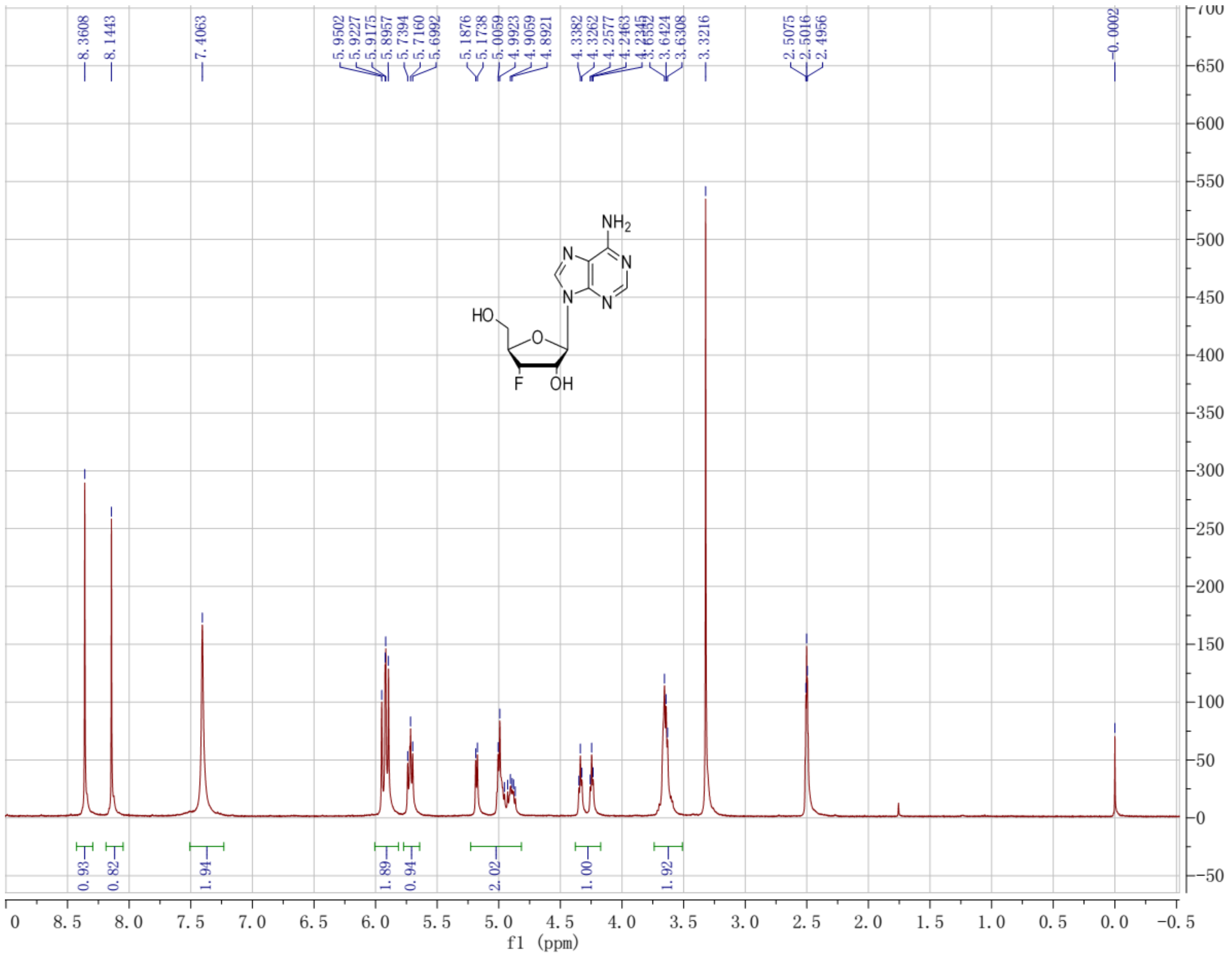

Product 2: HRMS

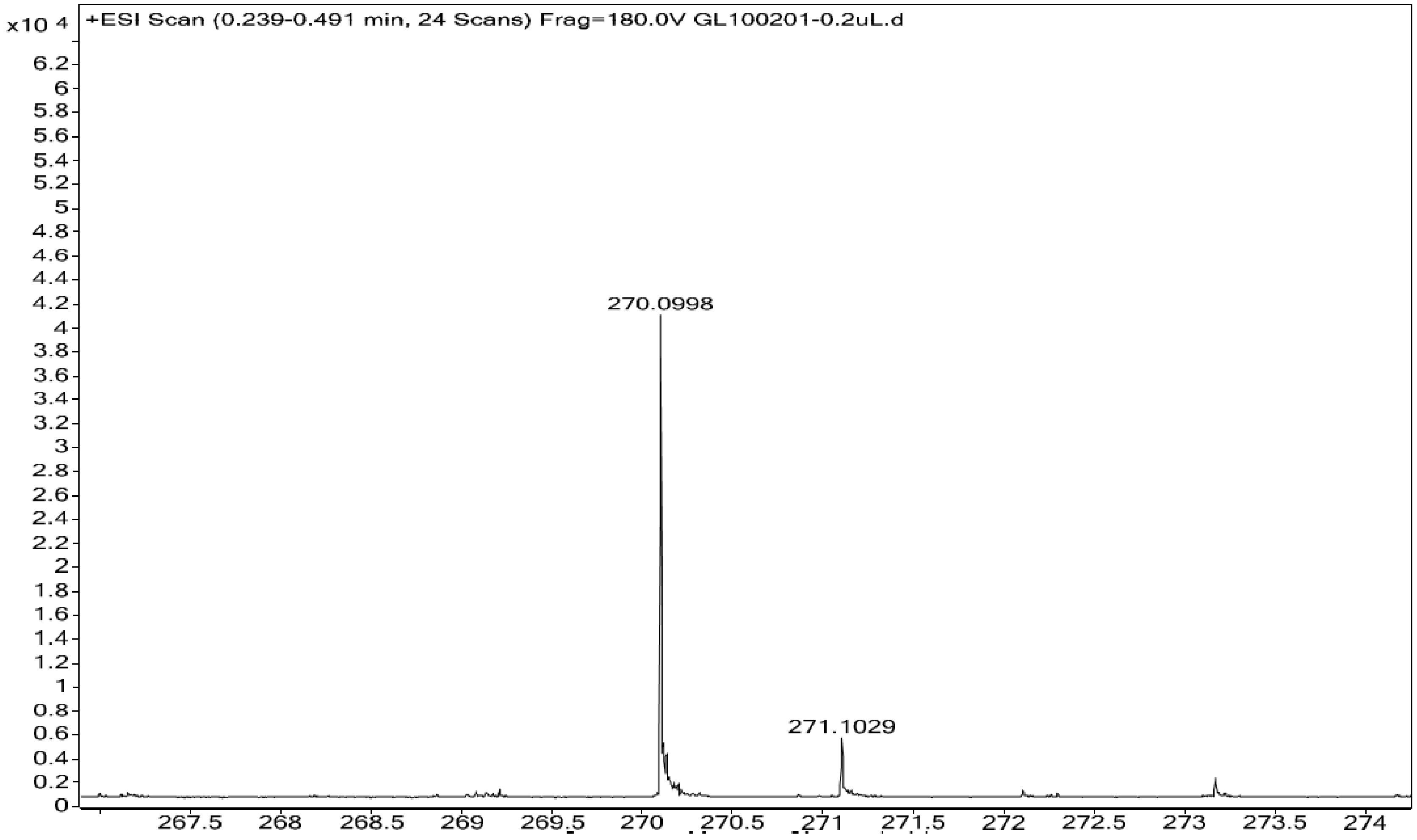

Product 3: HRMS

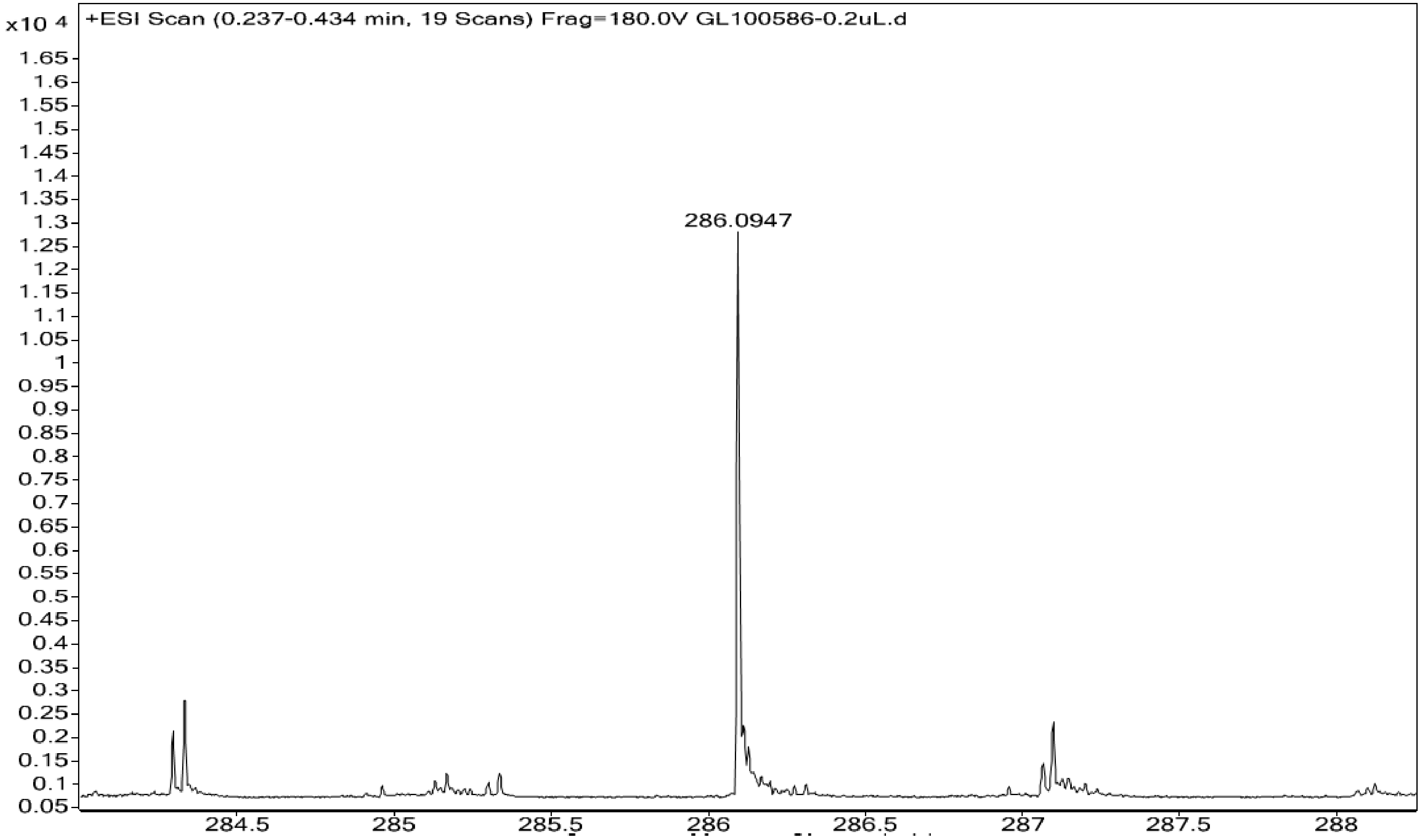

Product 4: ^{1}H NMR (400 MHz, DMSO-d_6)

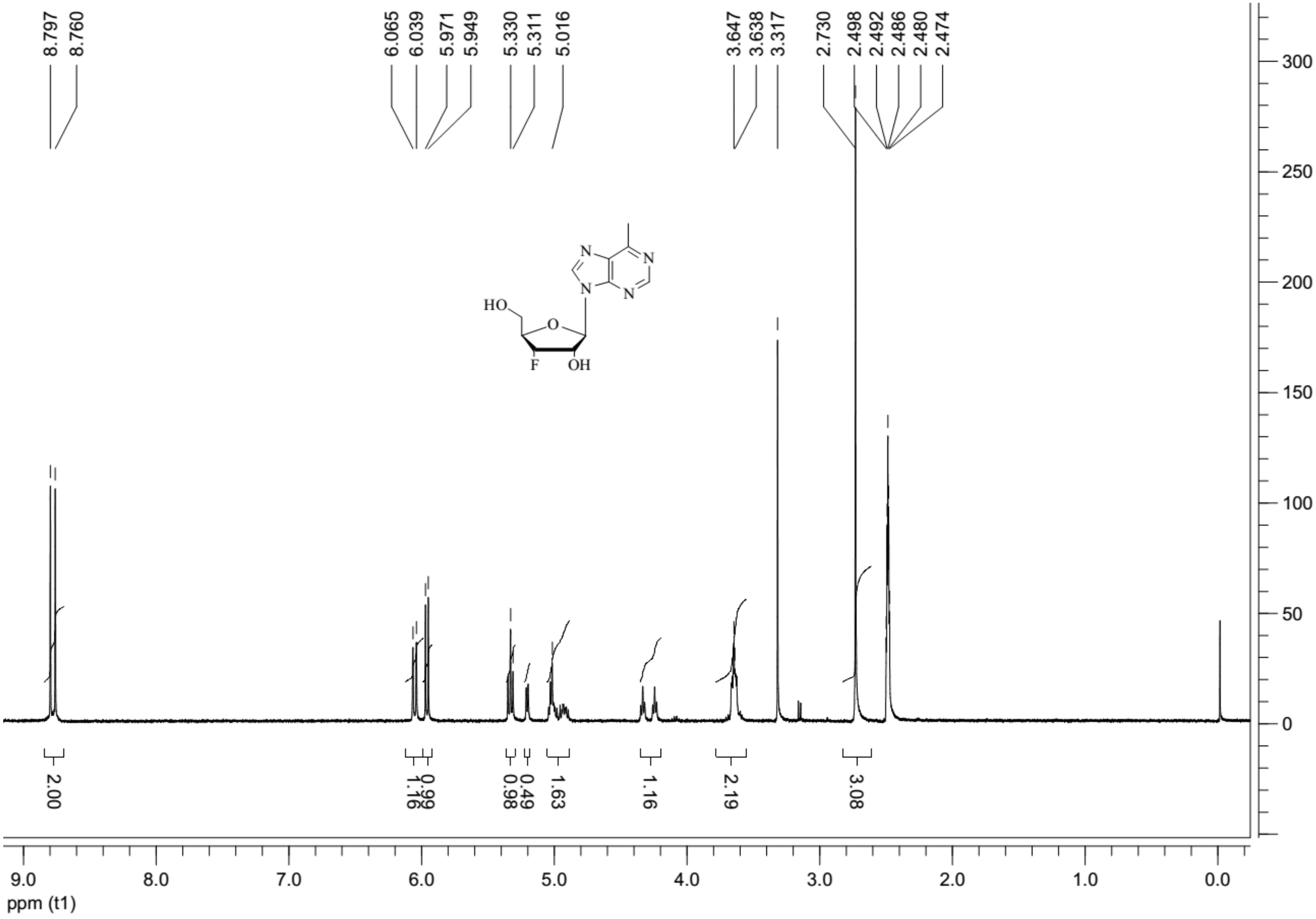

Product 4: HRMS

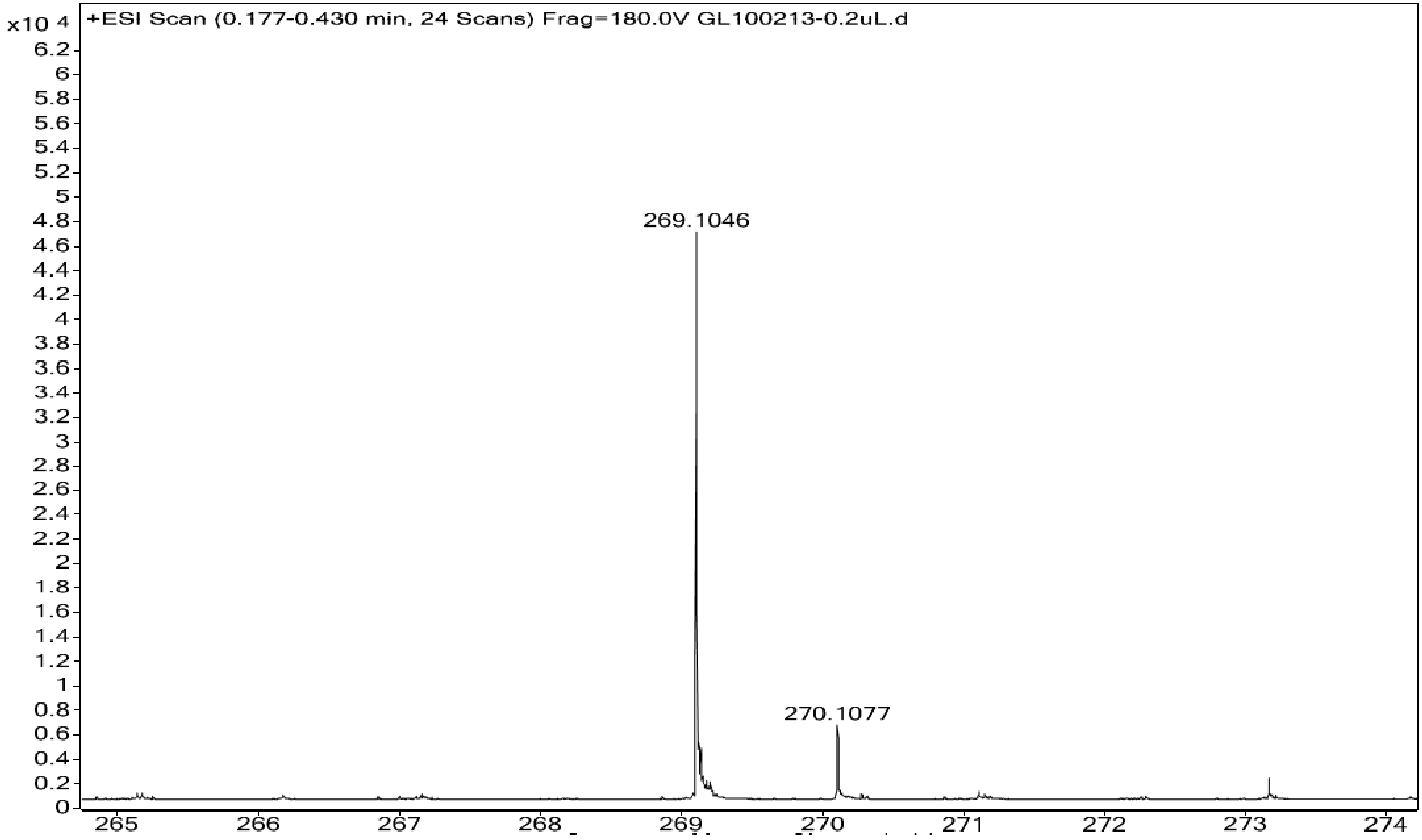

Product 5: ^{1}H NMR (400 MHz, DMSO-d_6)

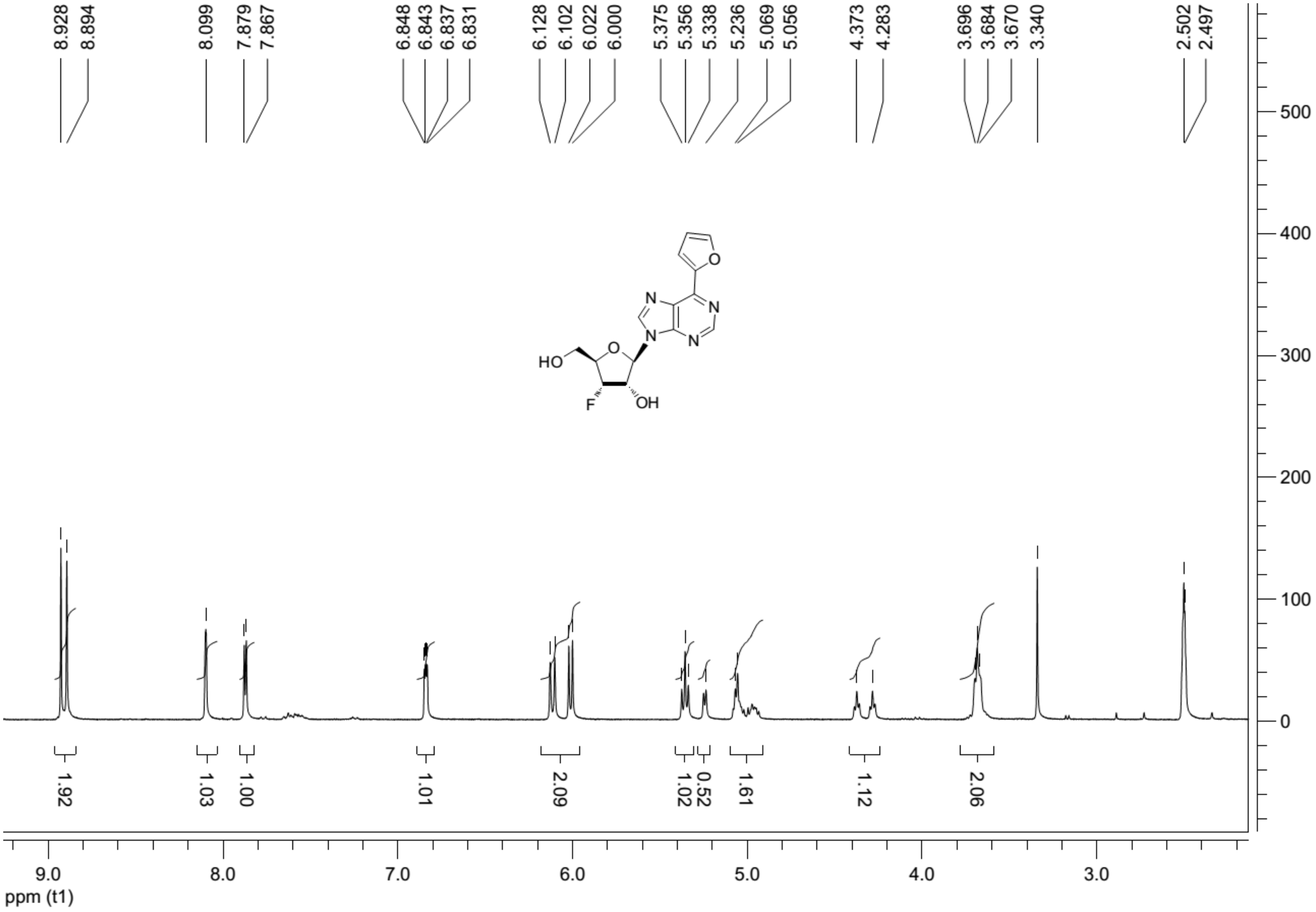

Product 6: ^{1}H NMR (400 MHz, DMSO-d_6)

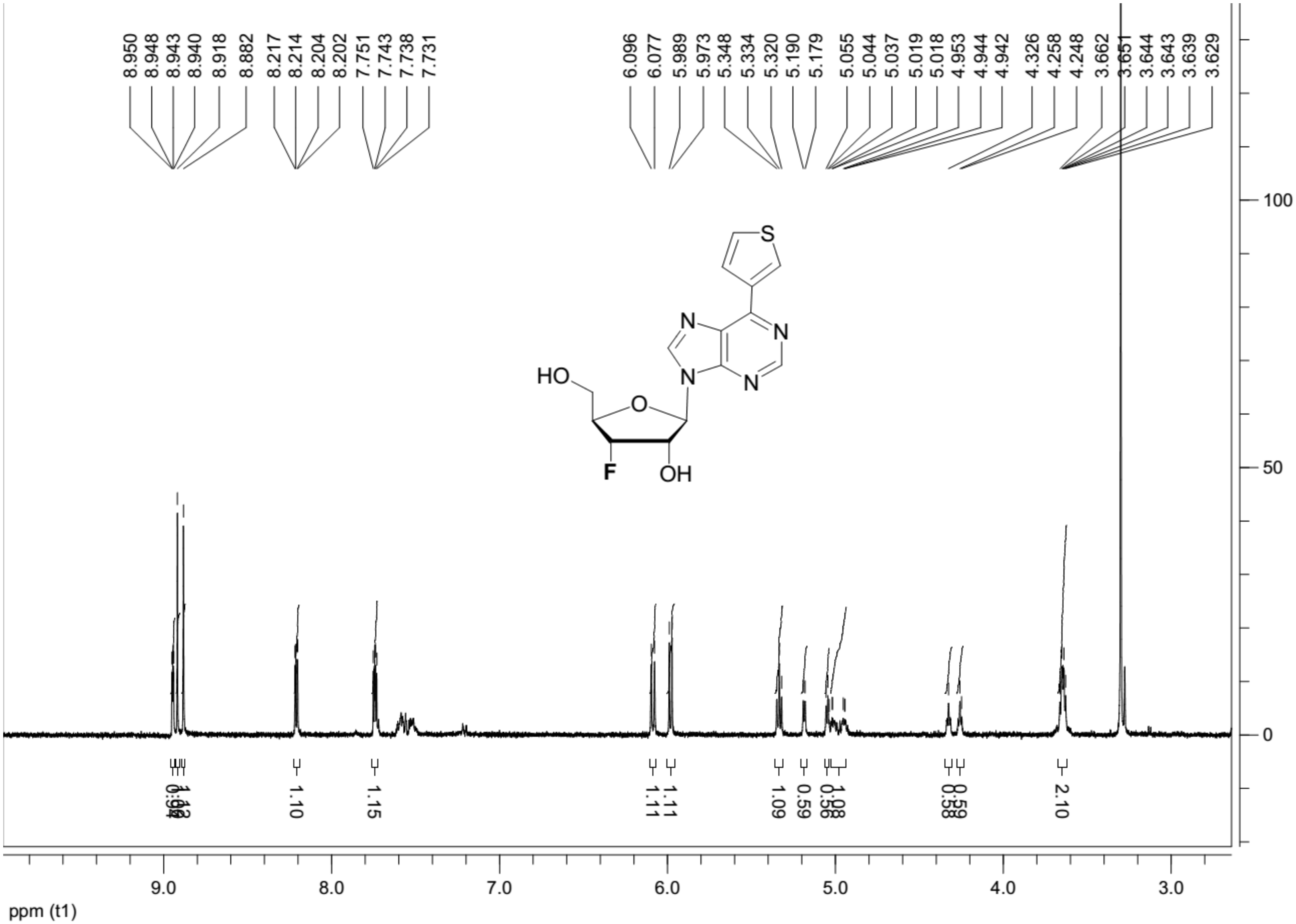

Product 6: HRMS

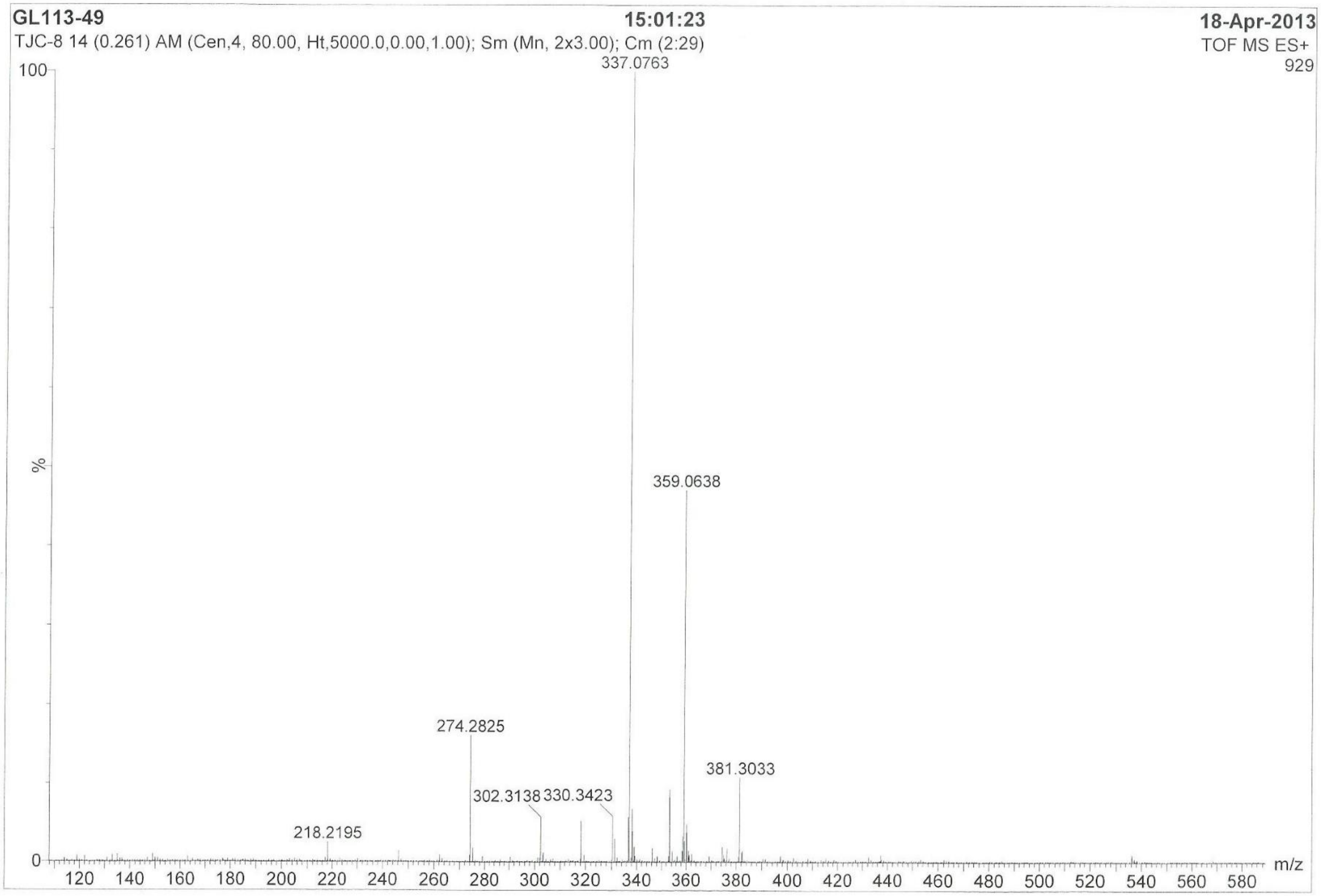

Product 7: ^{1}H NMR (400 MHz, DMSO-d_6)

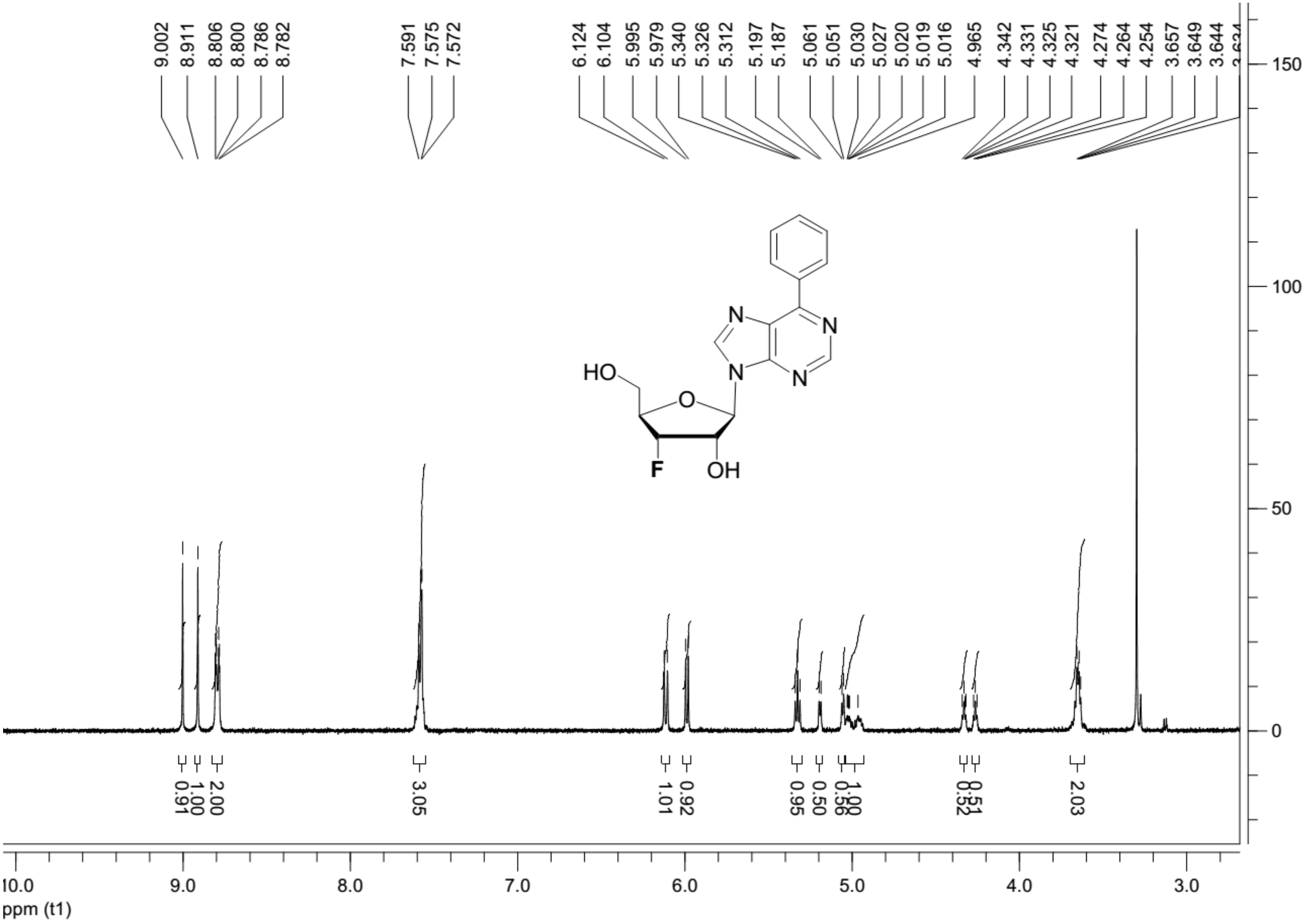

Product 7: HRMS

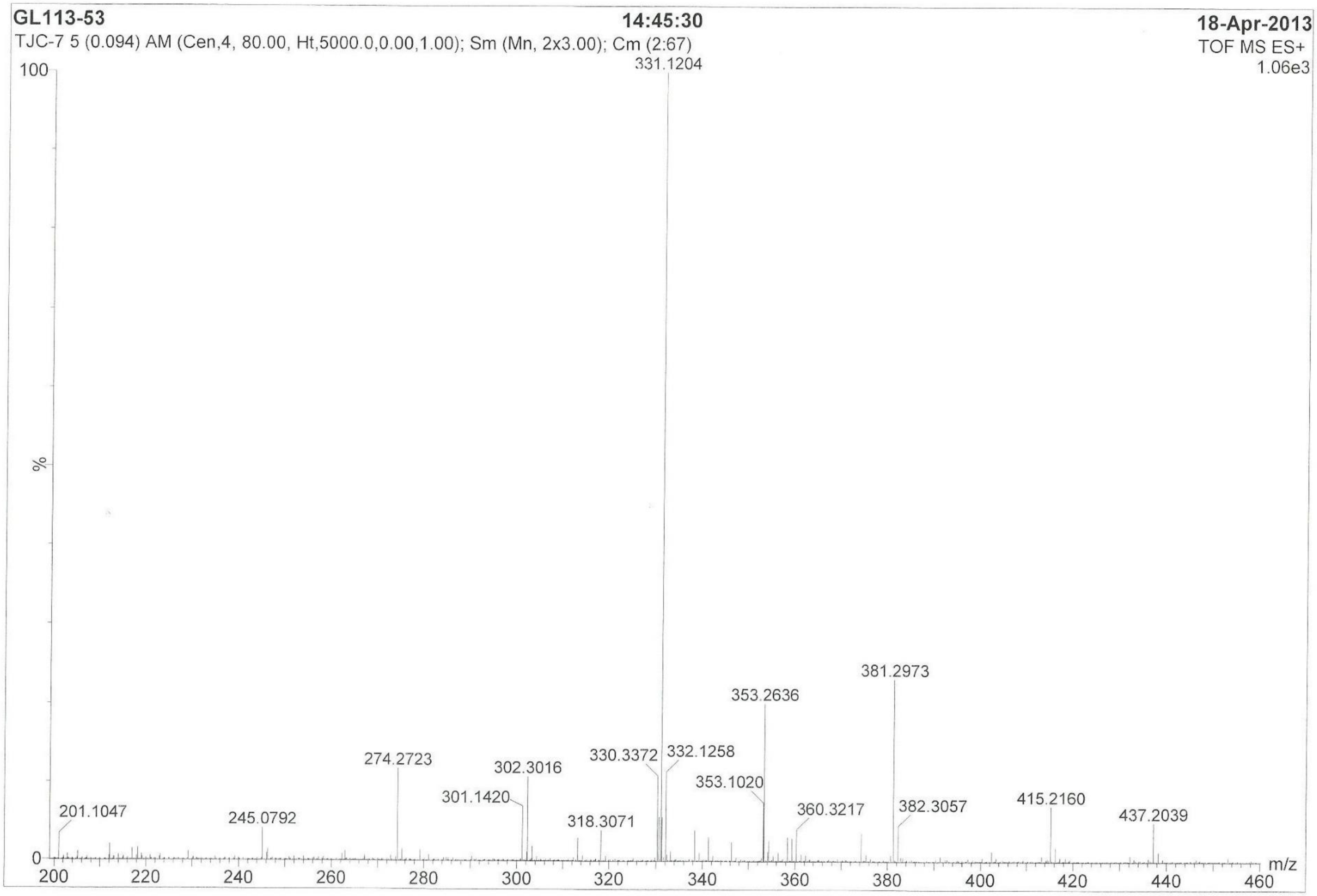

Product 8: ^{1}H NMR (400 MHz, DMSO-d_6)

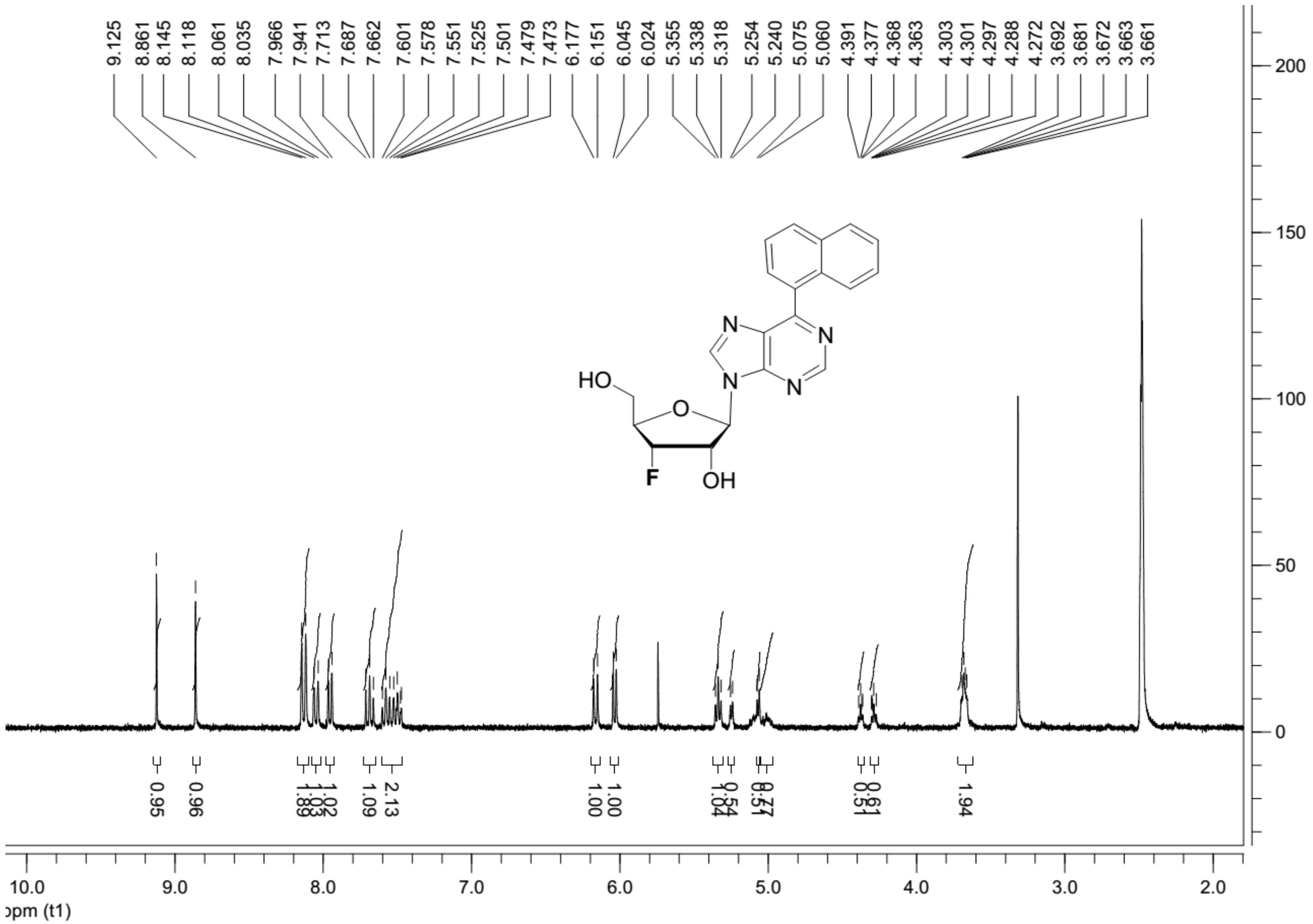

Product 8: HRMS

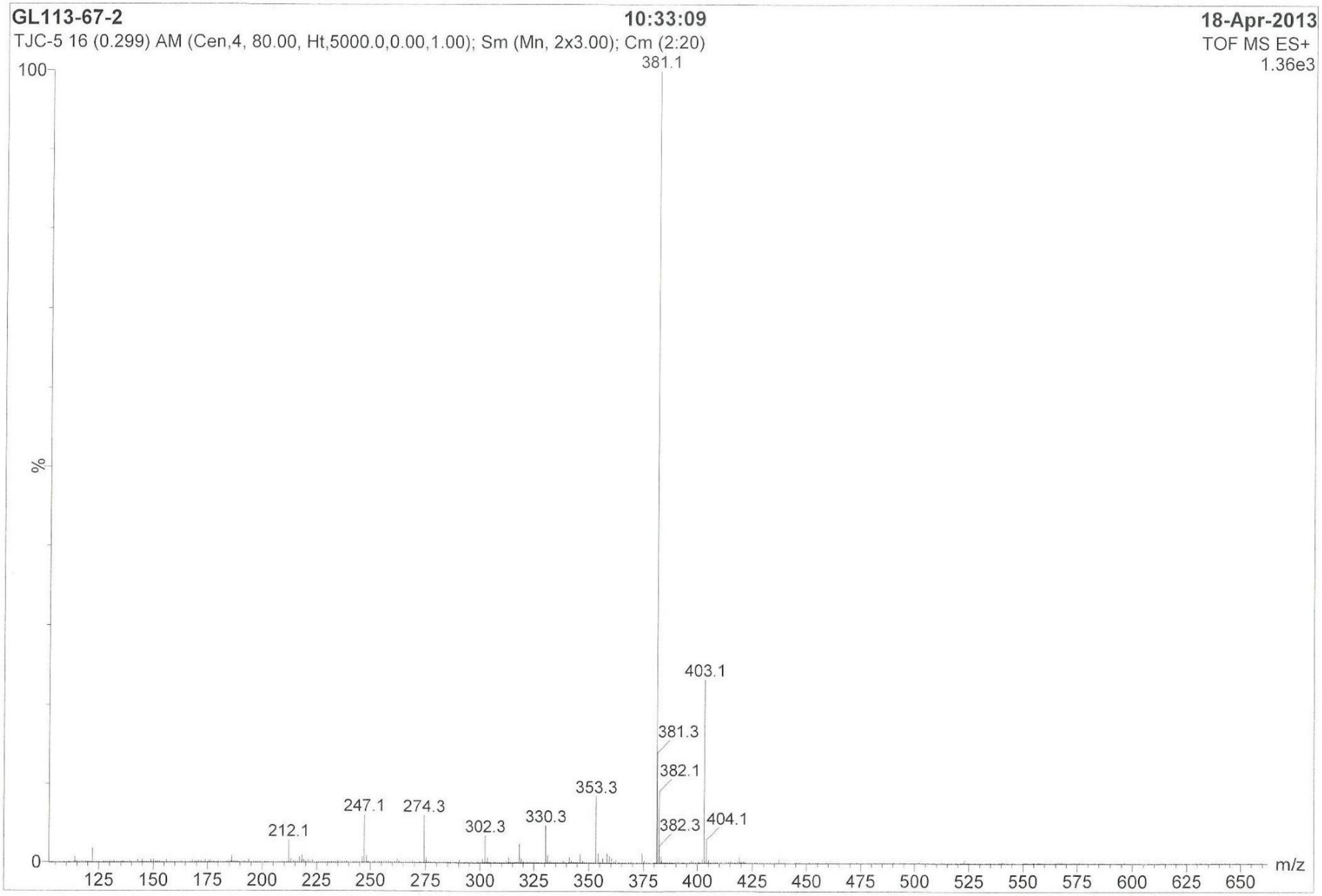

Product 9: ^{1}H NMR (400 MHz, DMSO-d_6)

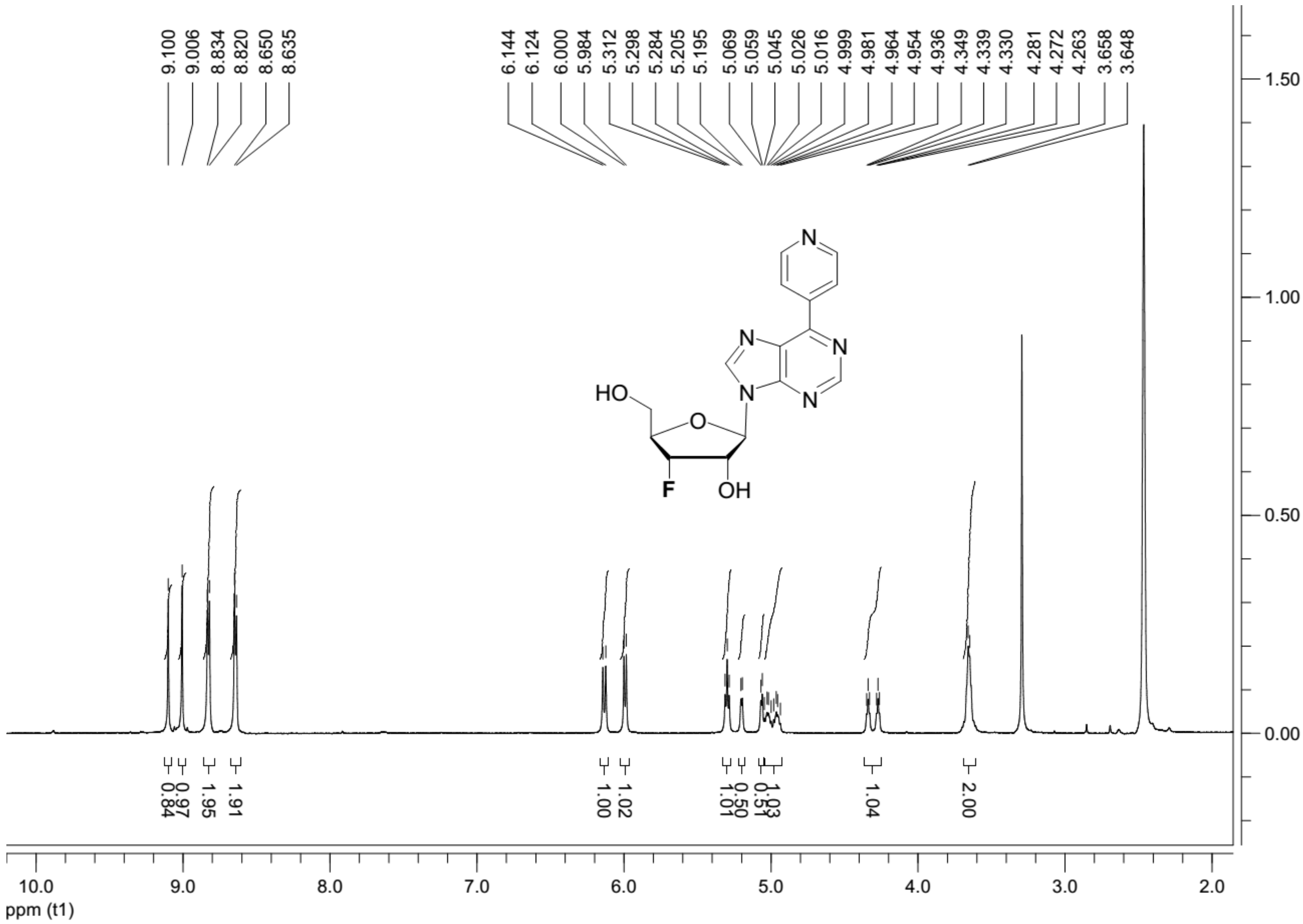

Product 9: HRMS

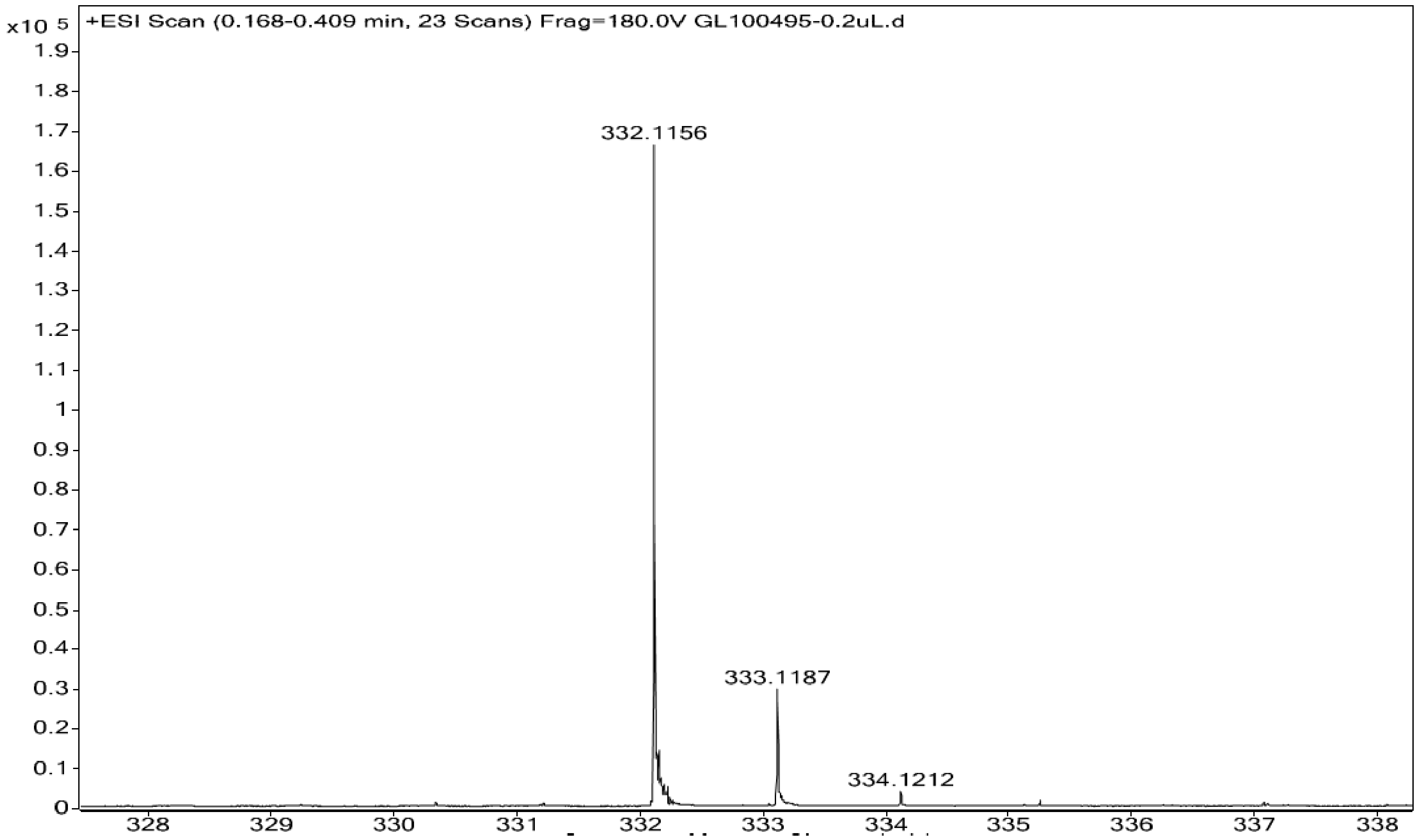

Product 10: ^{1}H NMR (400 MHz, DMSO-d_6)

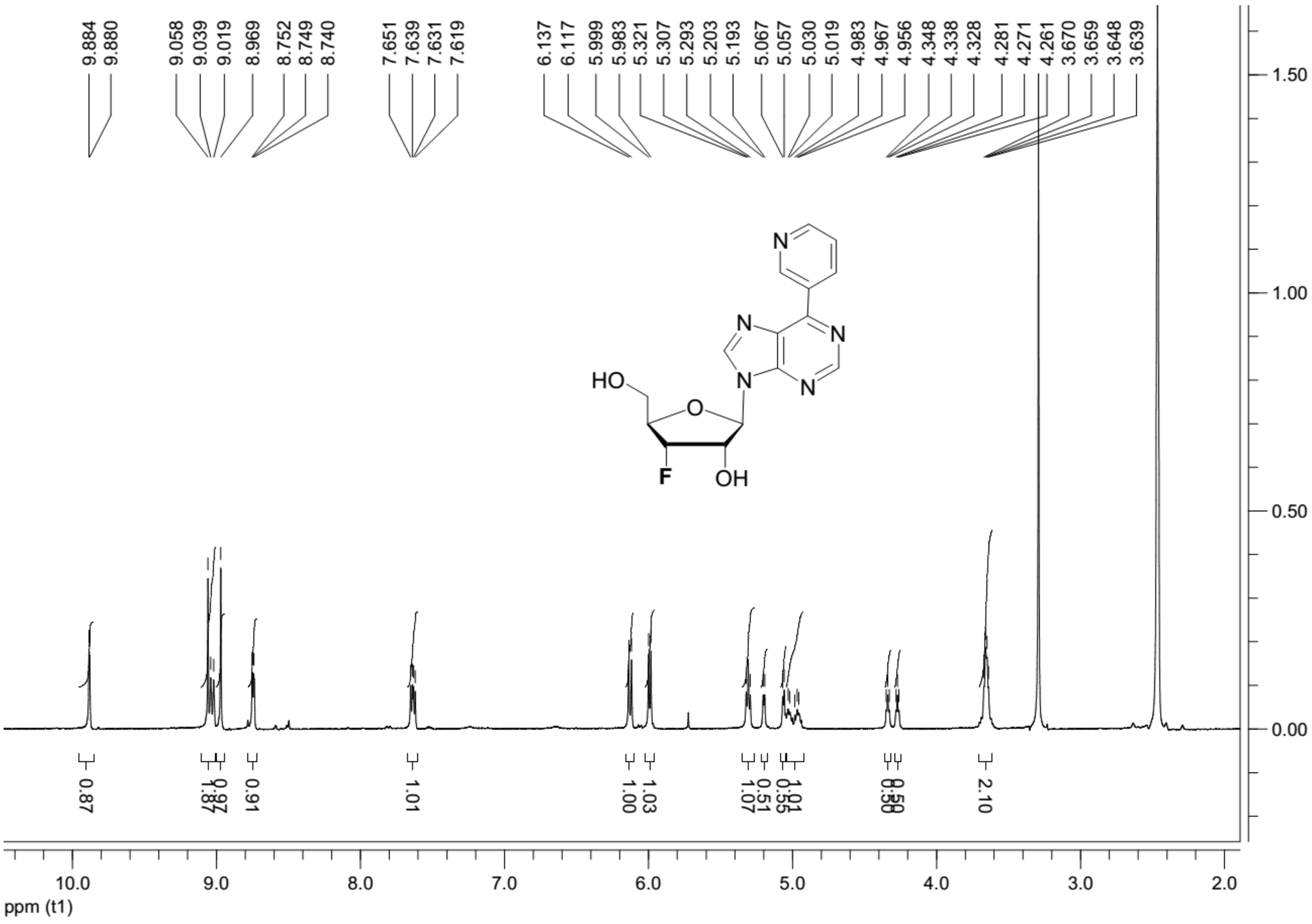

Product 10: HRMS

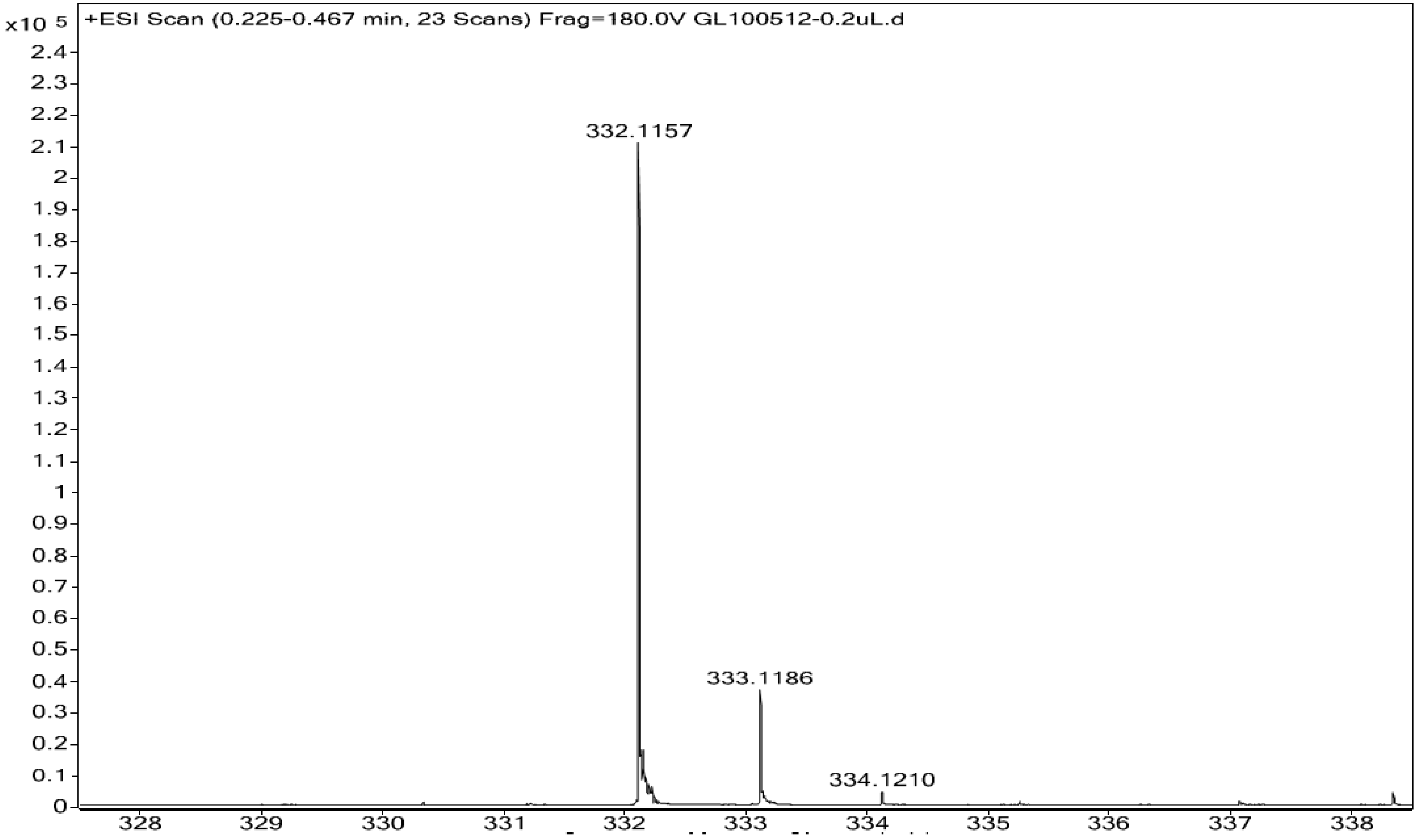

Product 11: ^{1}H NMR (400 MHz, DMSO-d_6)

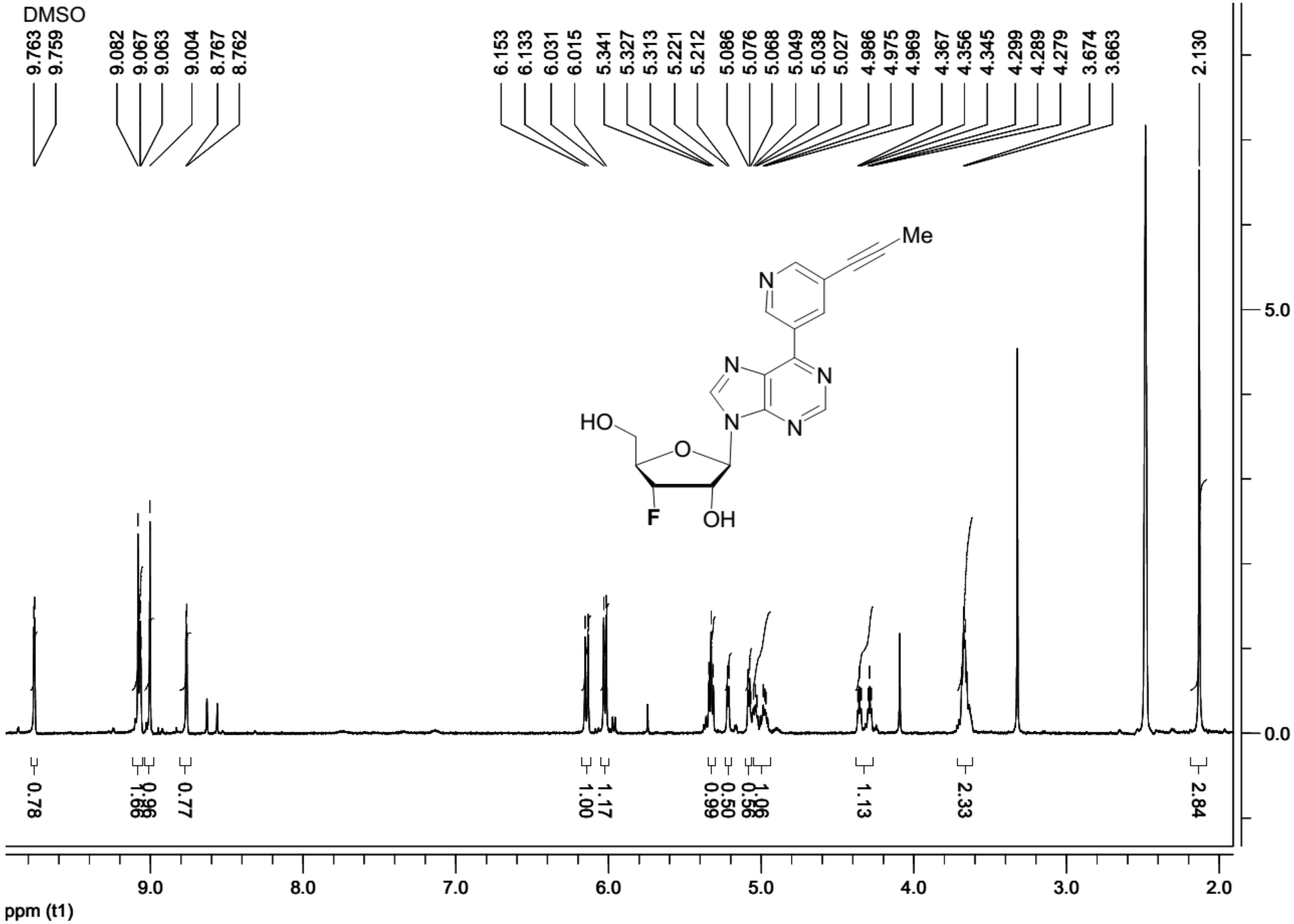

Product 11: HRMS

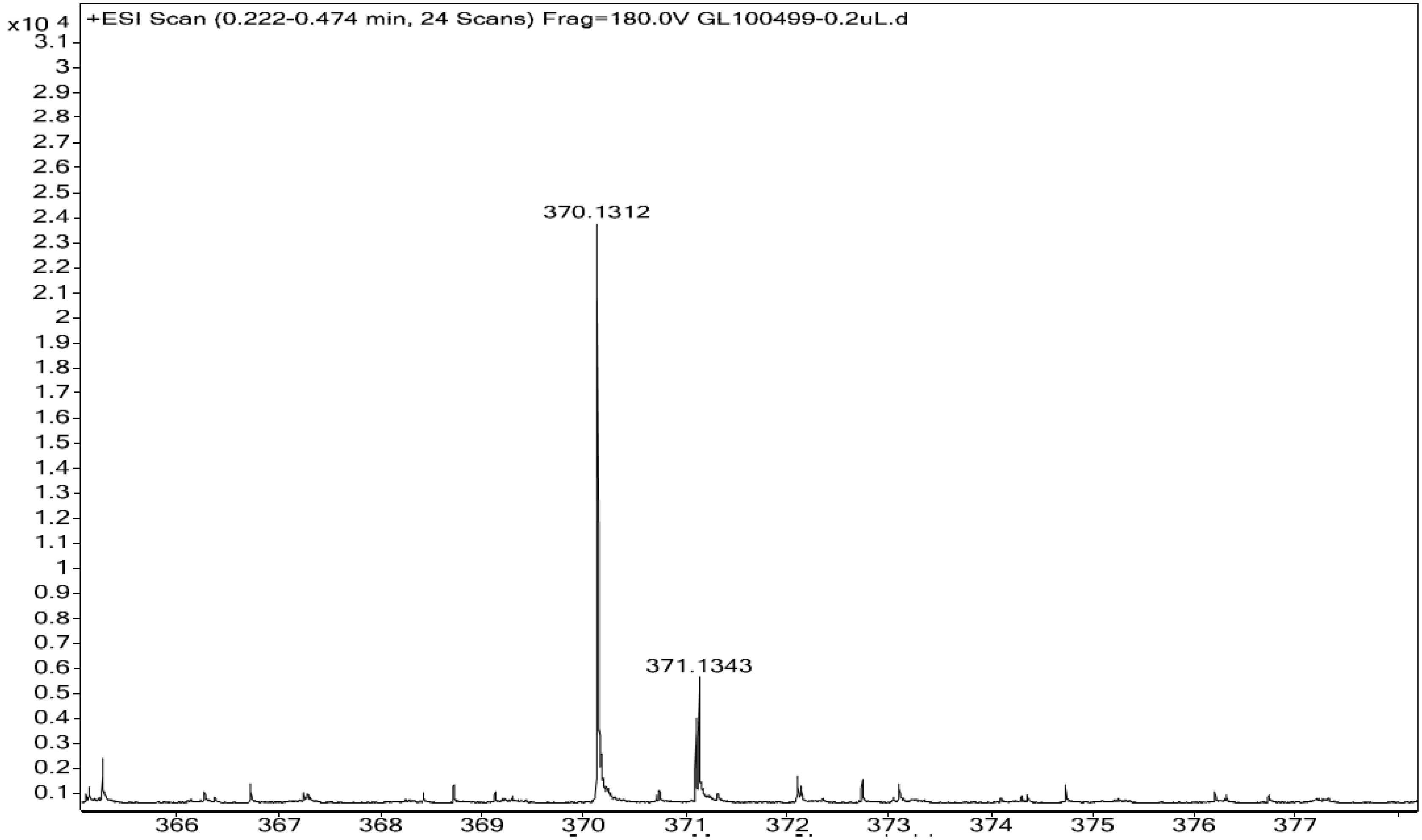

Product 12: ^{1}H NMR (400 MHz, DMSO-d_6)

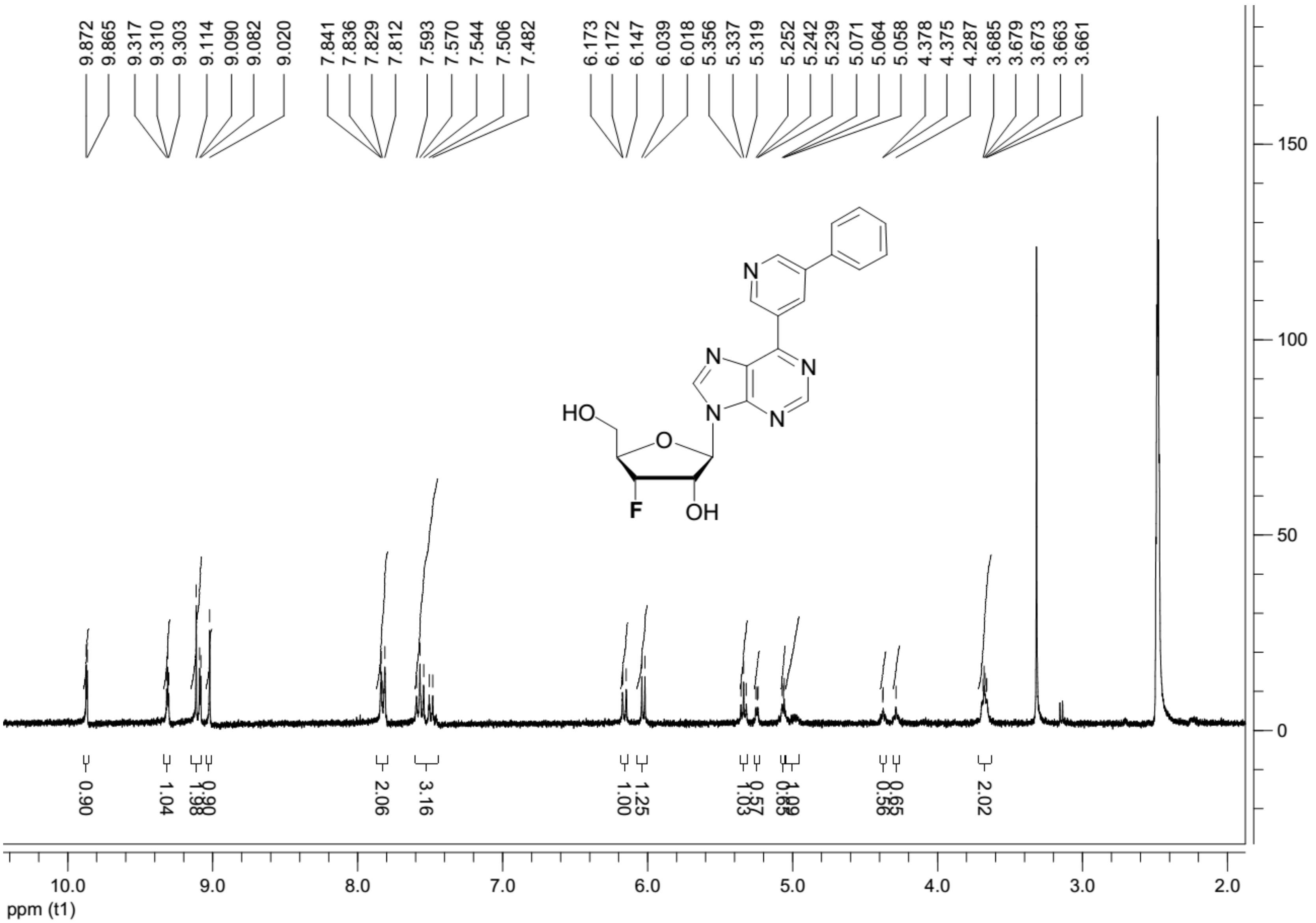

Product 12: HRMS

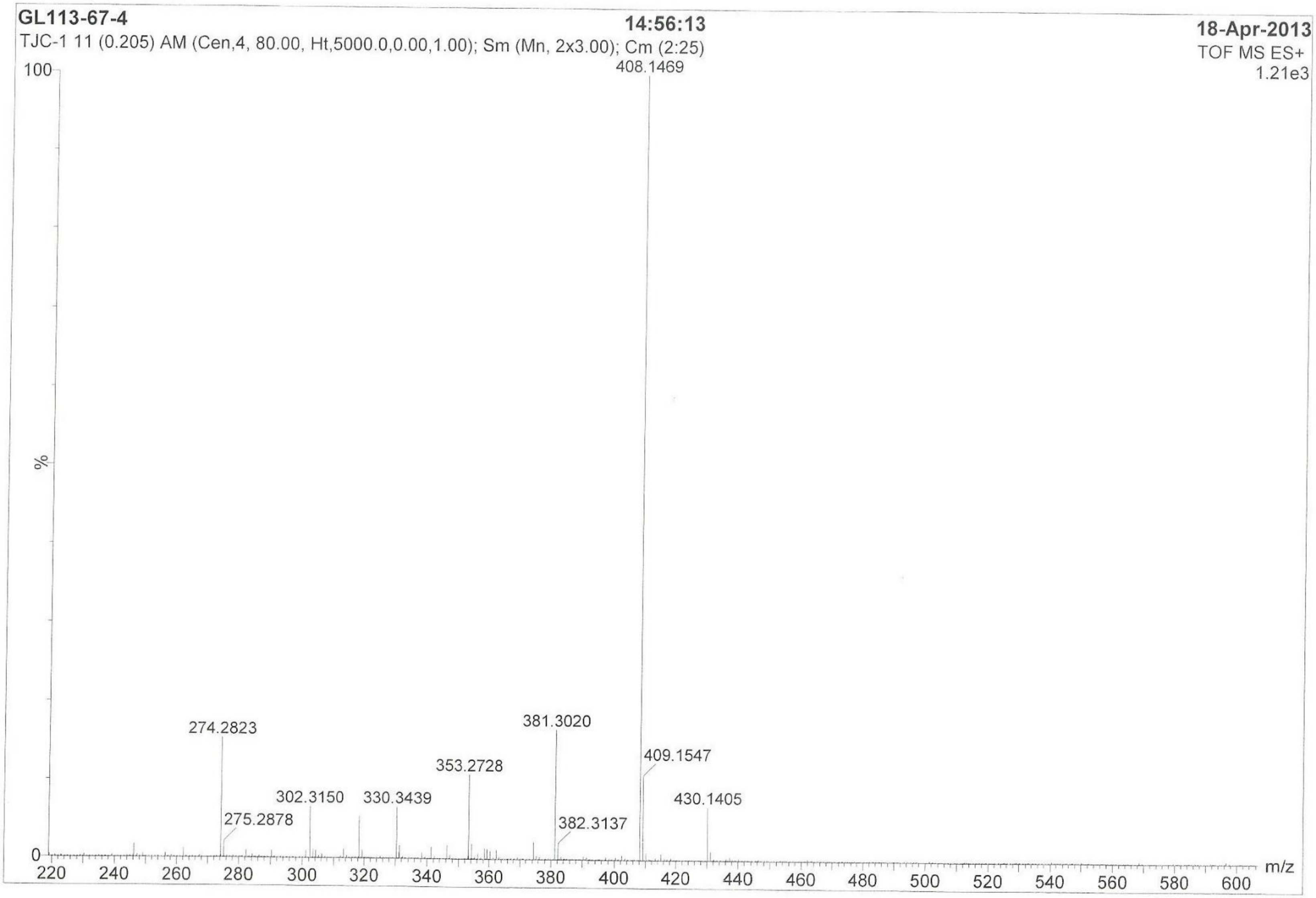

Product 13: ^{1}H NMR (400 MHz, DMSO-d_6)

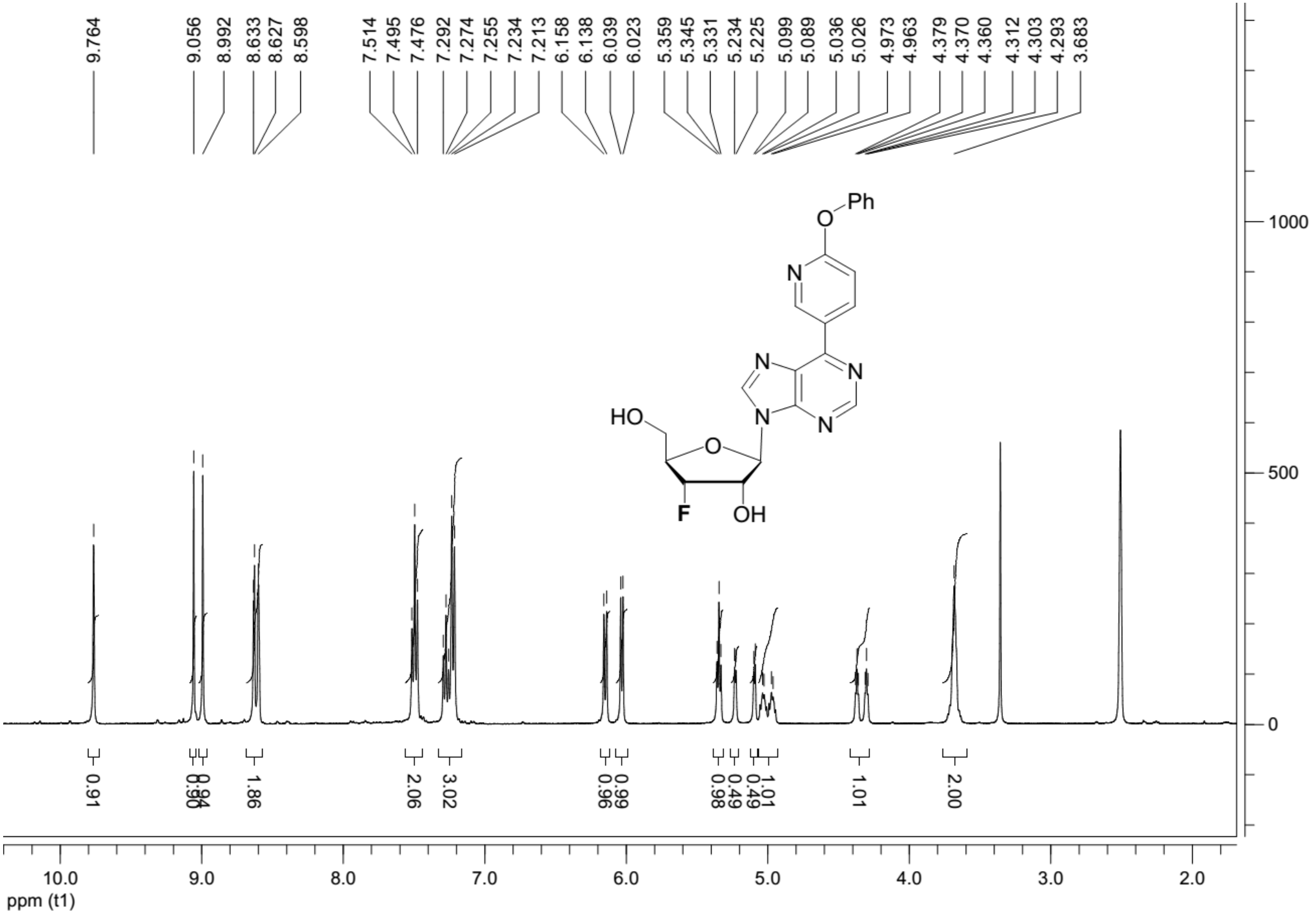

Product 13: HRMS

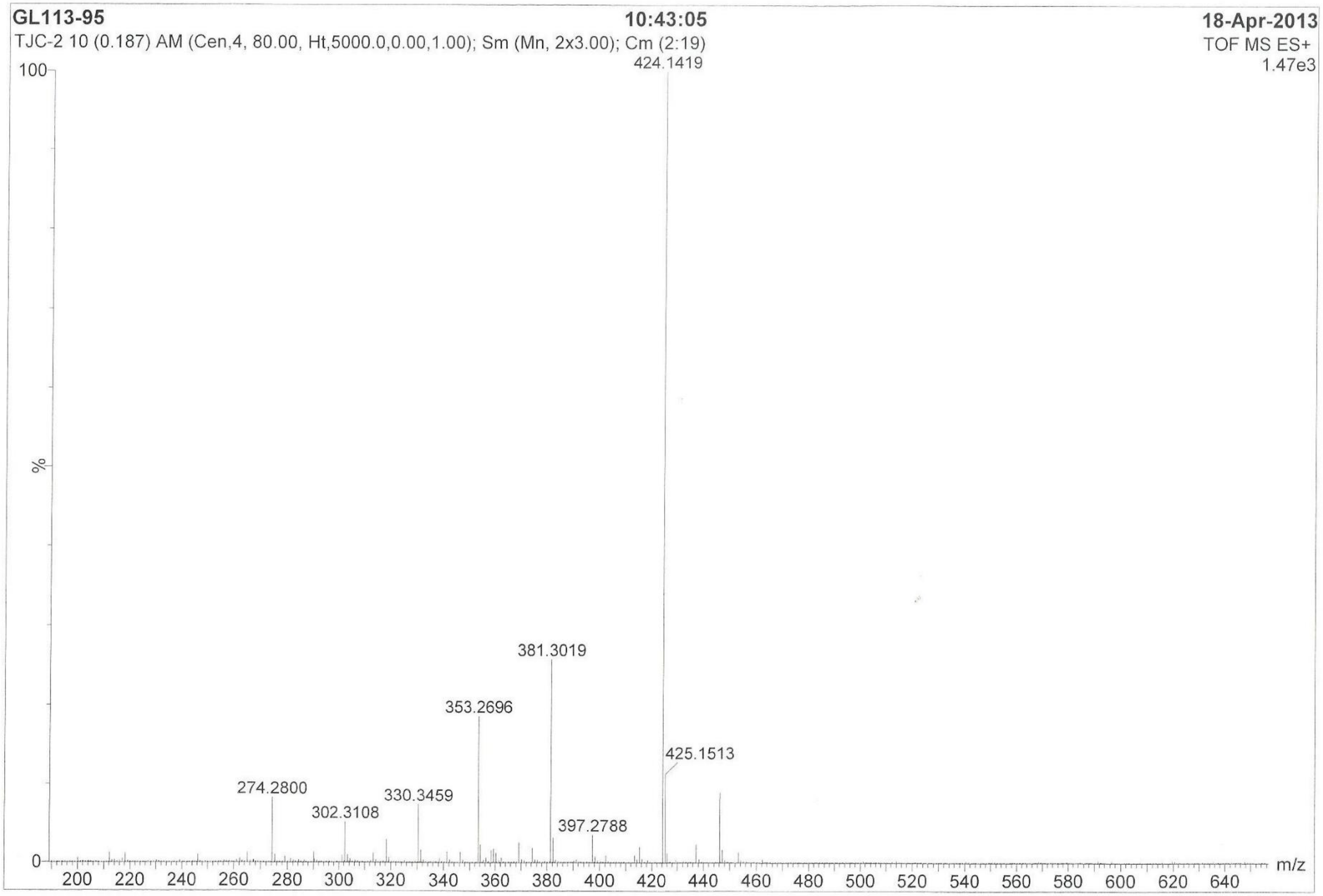

Product 14: ^{1}H NMR (400 MHz, DMSO-d_6)

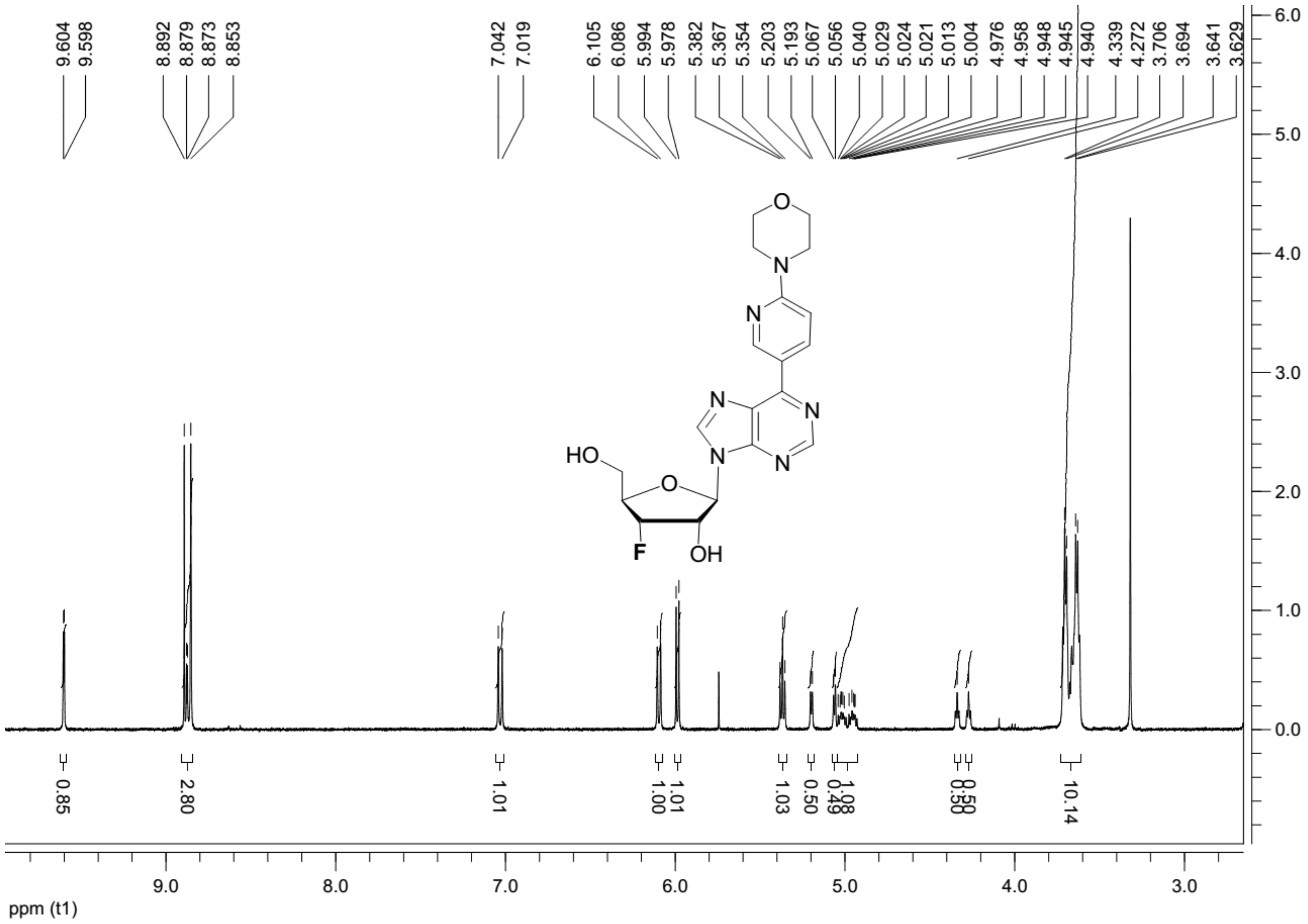

Product 14: HRMS

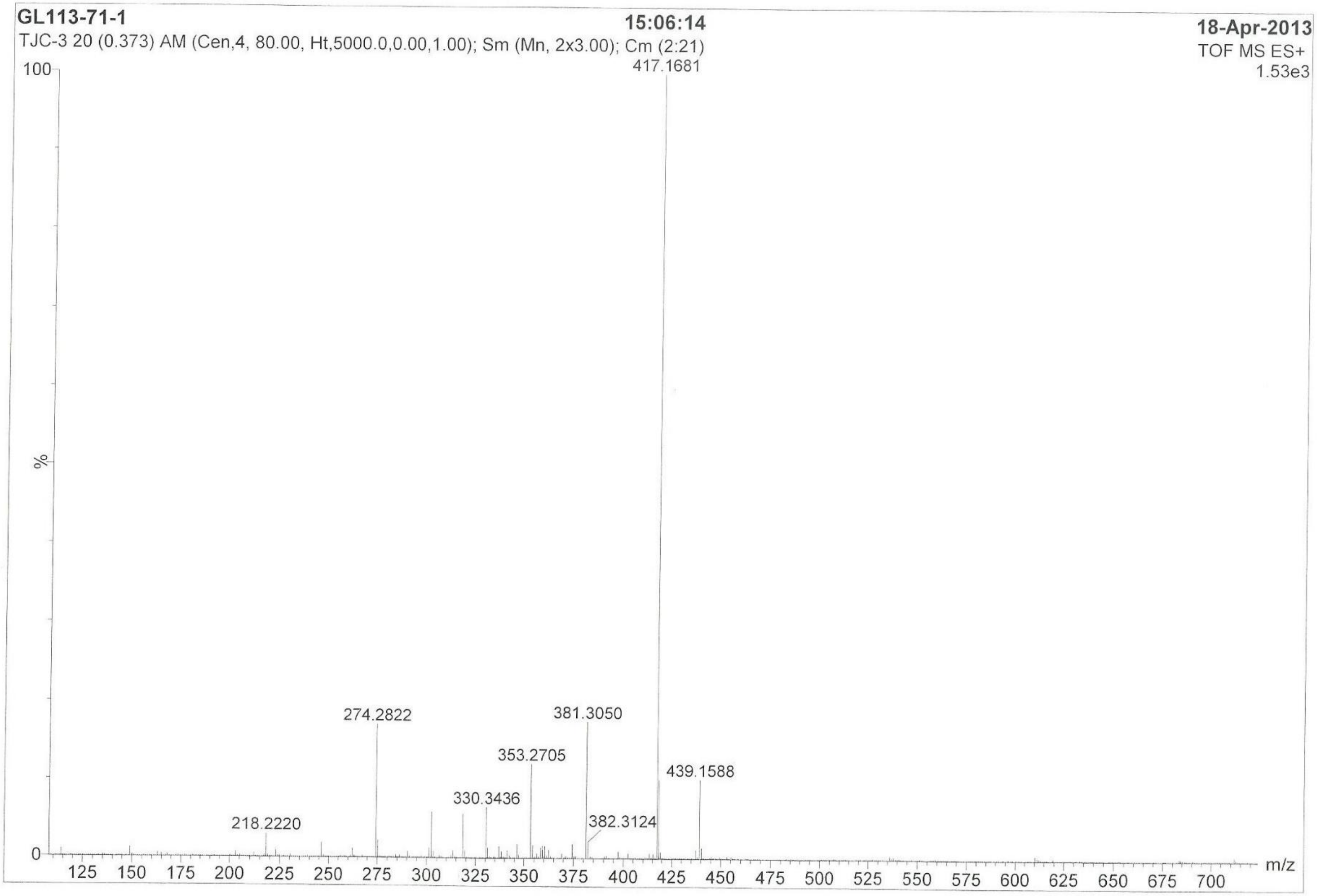

Product 15: ^{1}H NMR (400 MHz, DMSO-d_6)

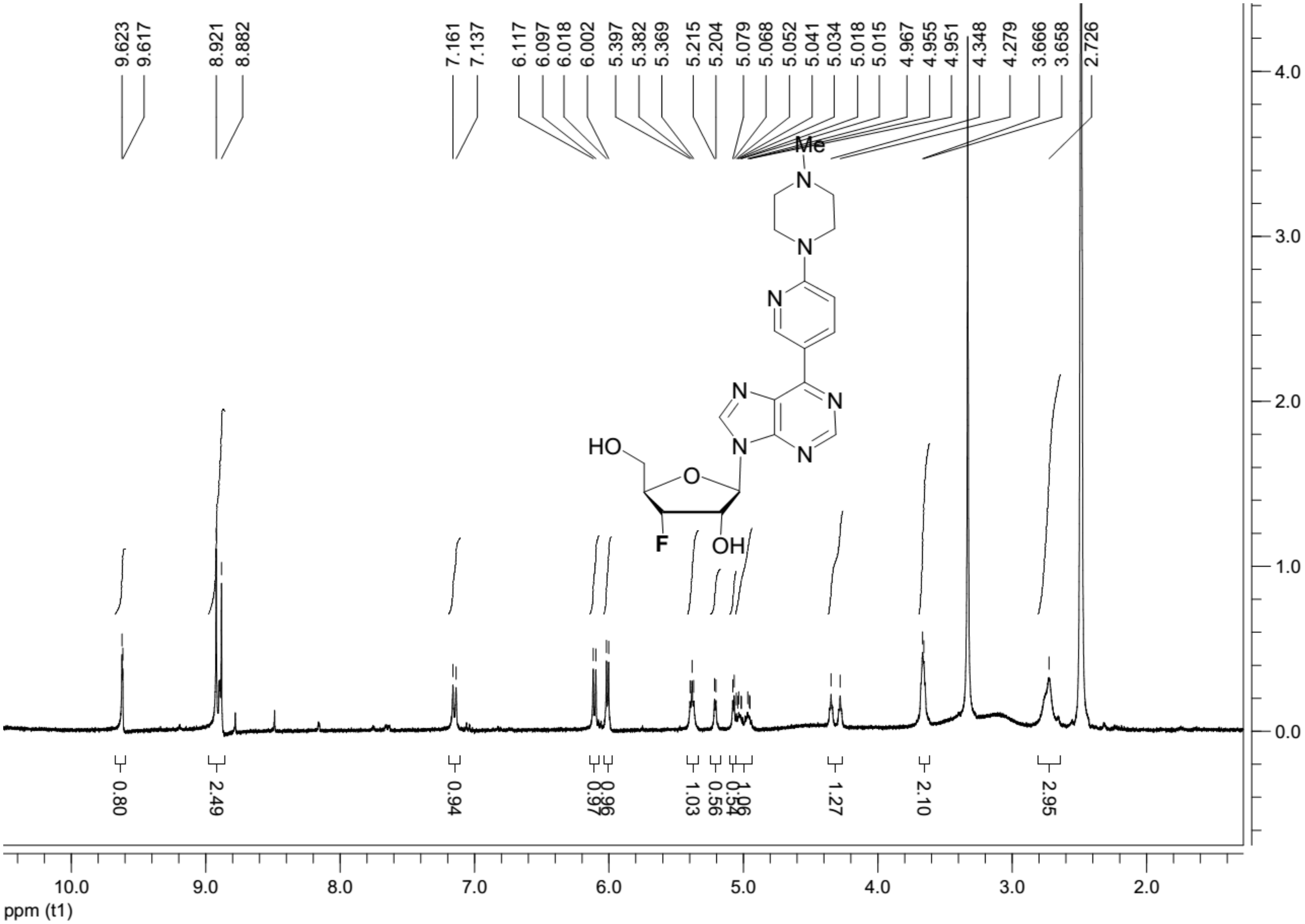

Product 15: HRMS

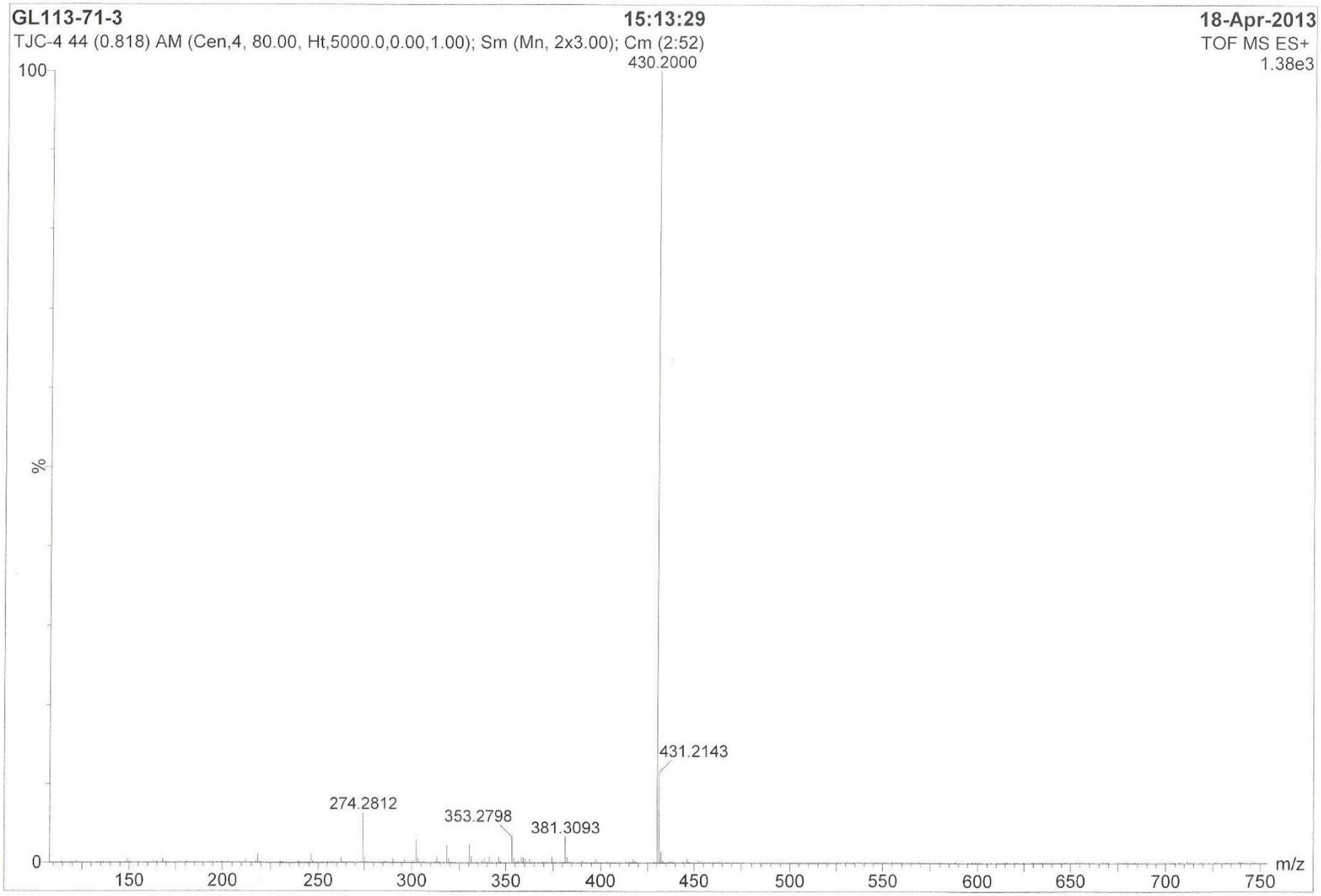

Product 16: ^{1}H NMR (400 MHz, DMSO-d_6)

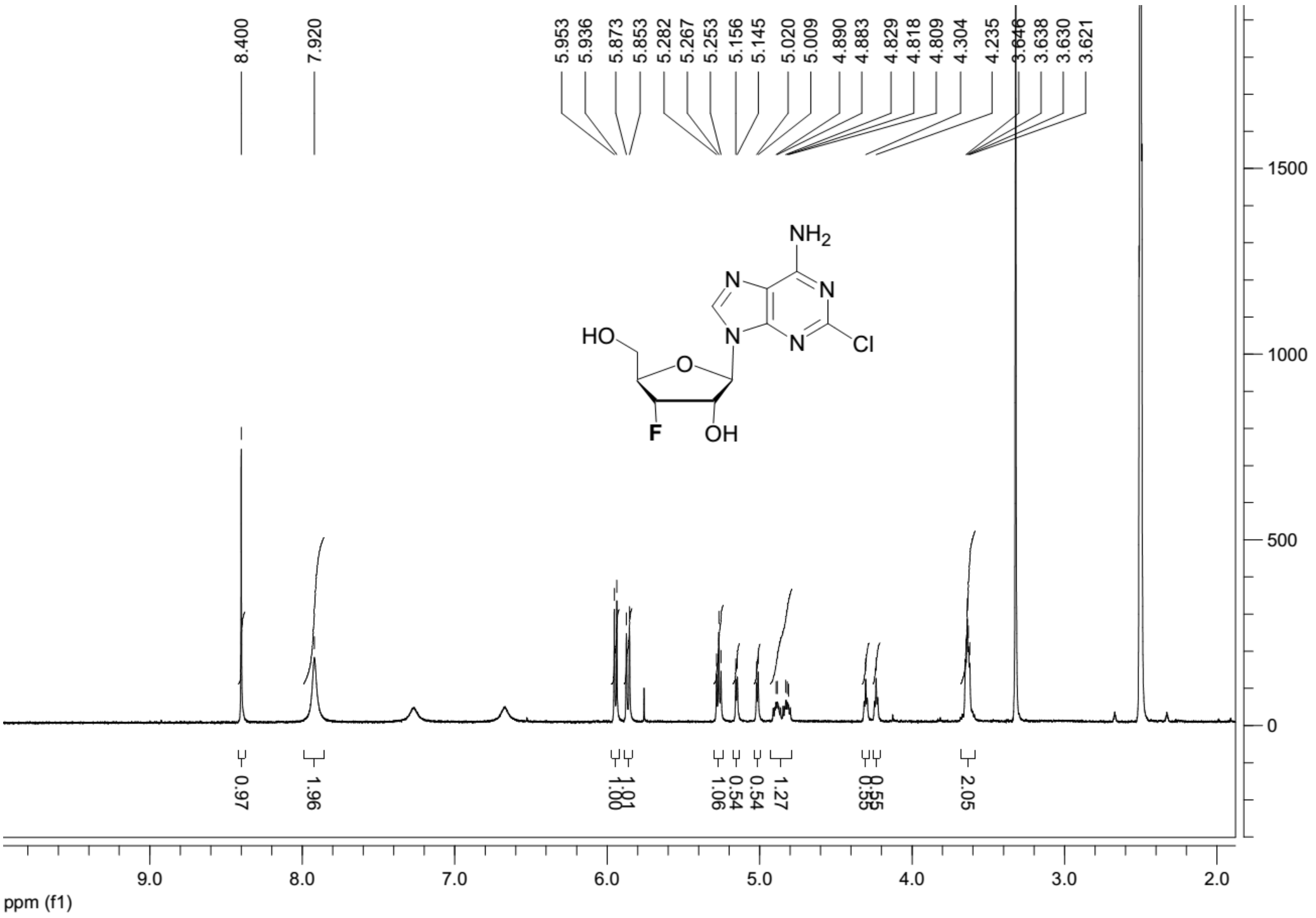

Product 16: HRMS

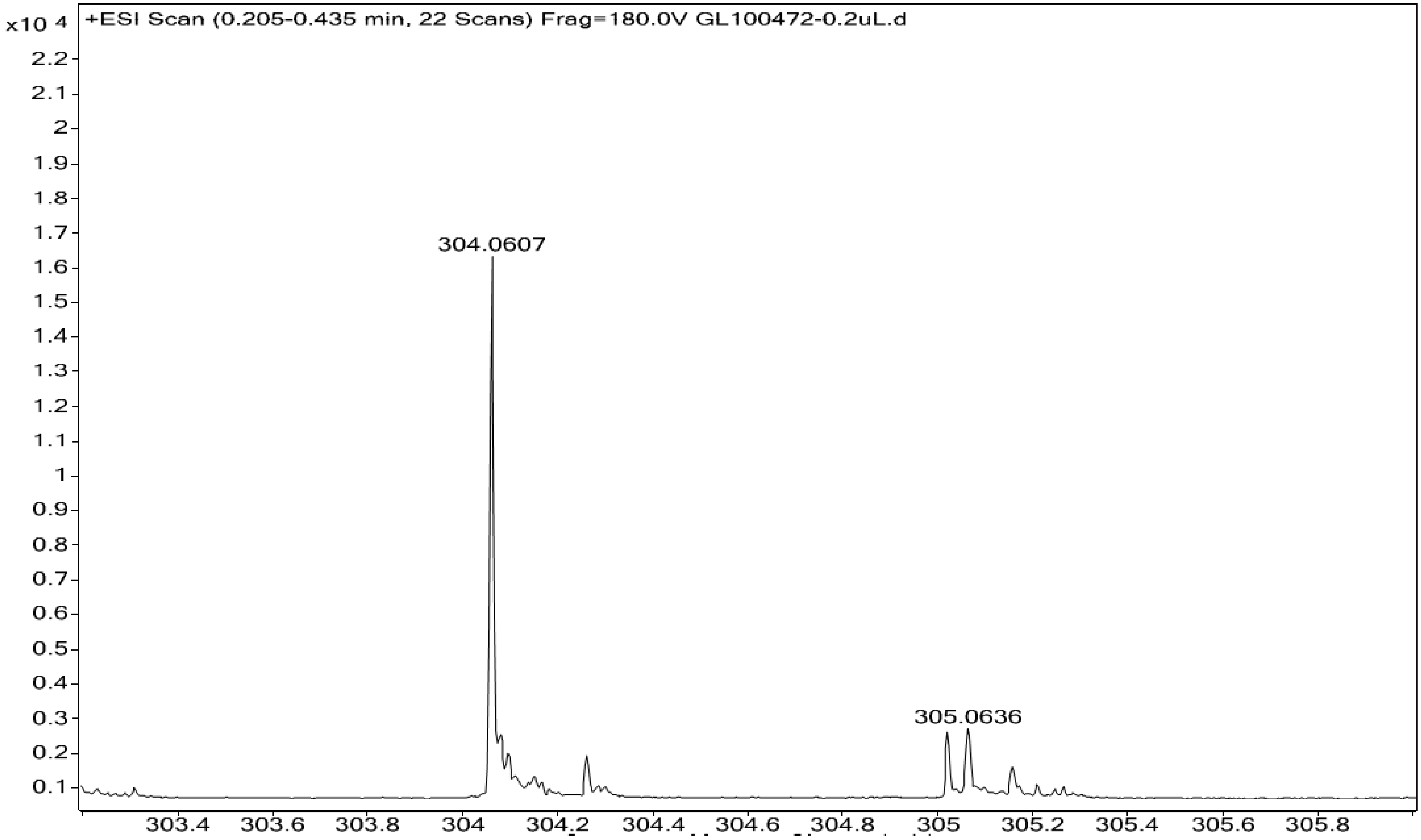

Product 17: ^{1}H NMR (400 MHz, DMSO-d_6)

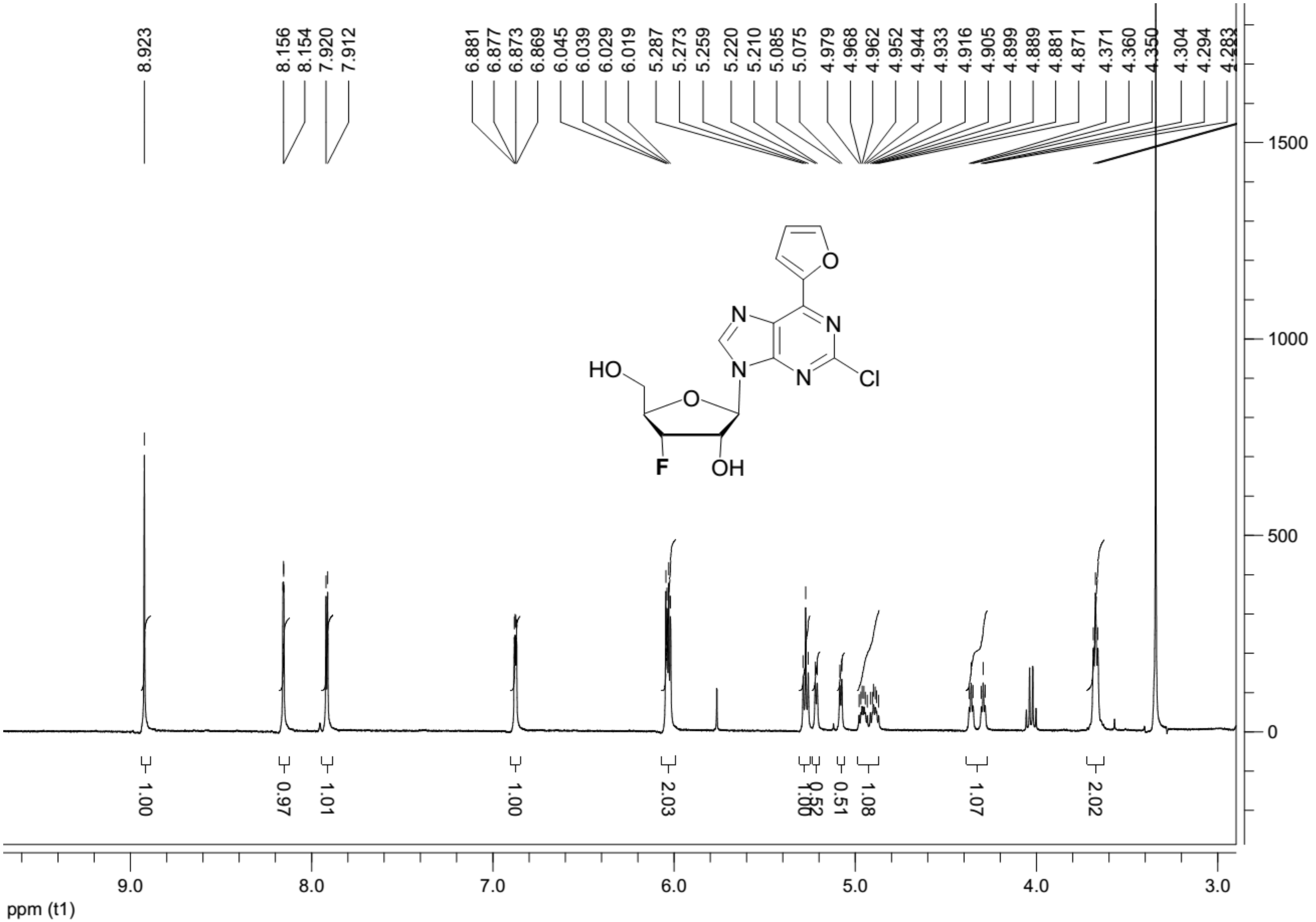

Product 17: HRMS

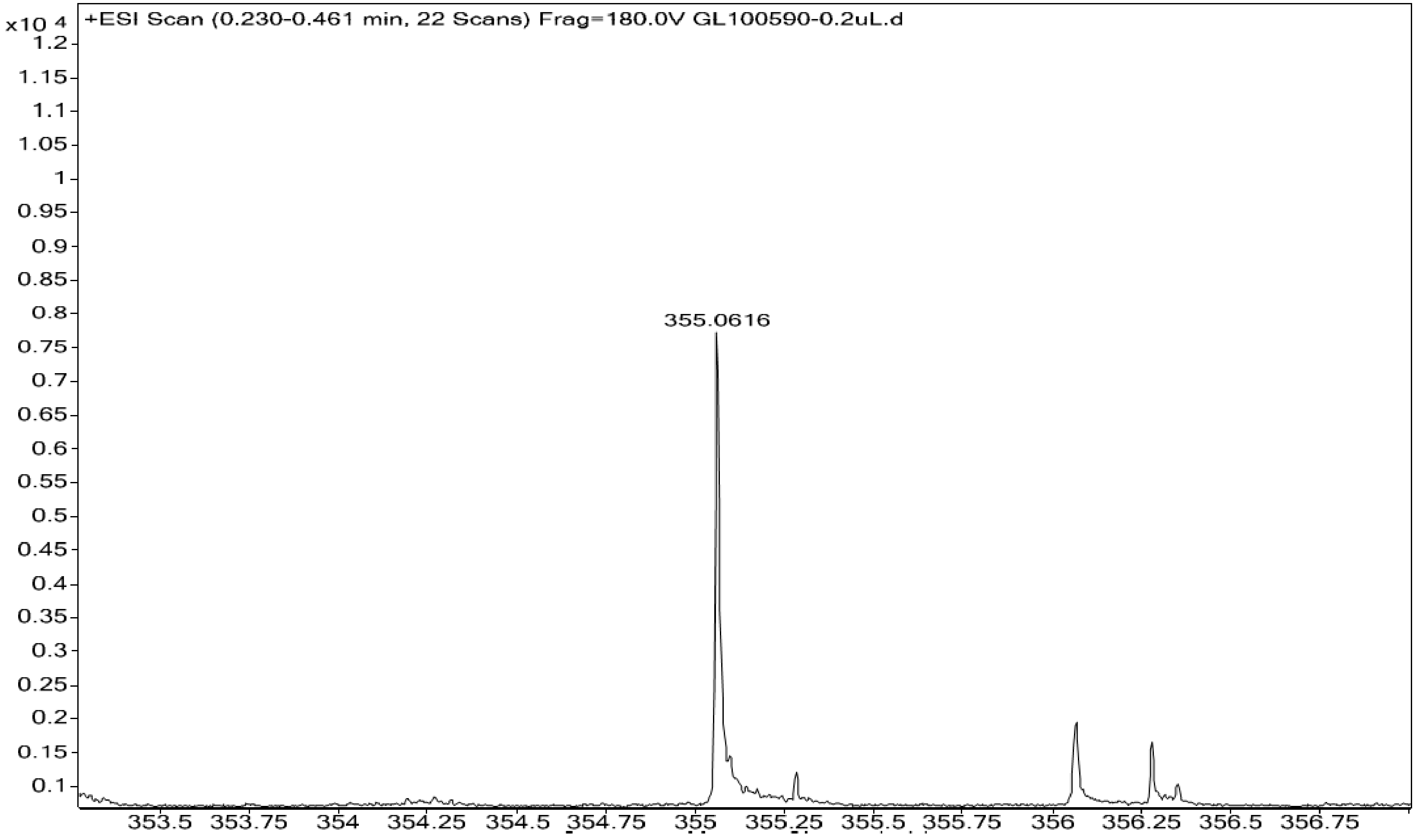

Product 18: ^{1}H NMR (400 MHz, DMSO-d_6)

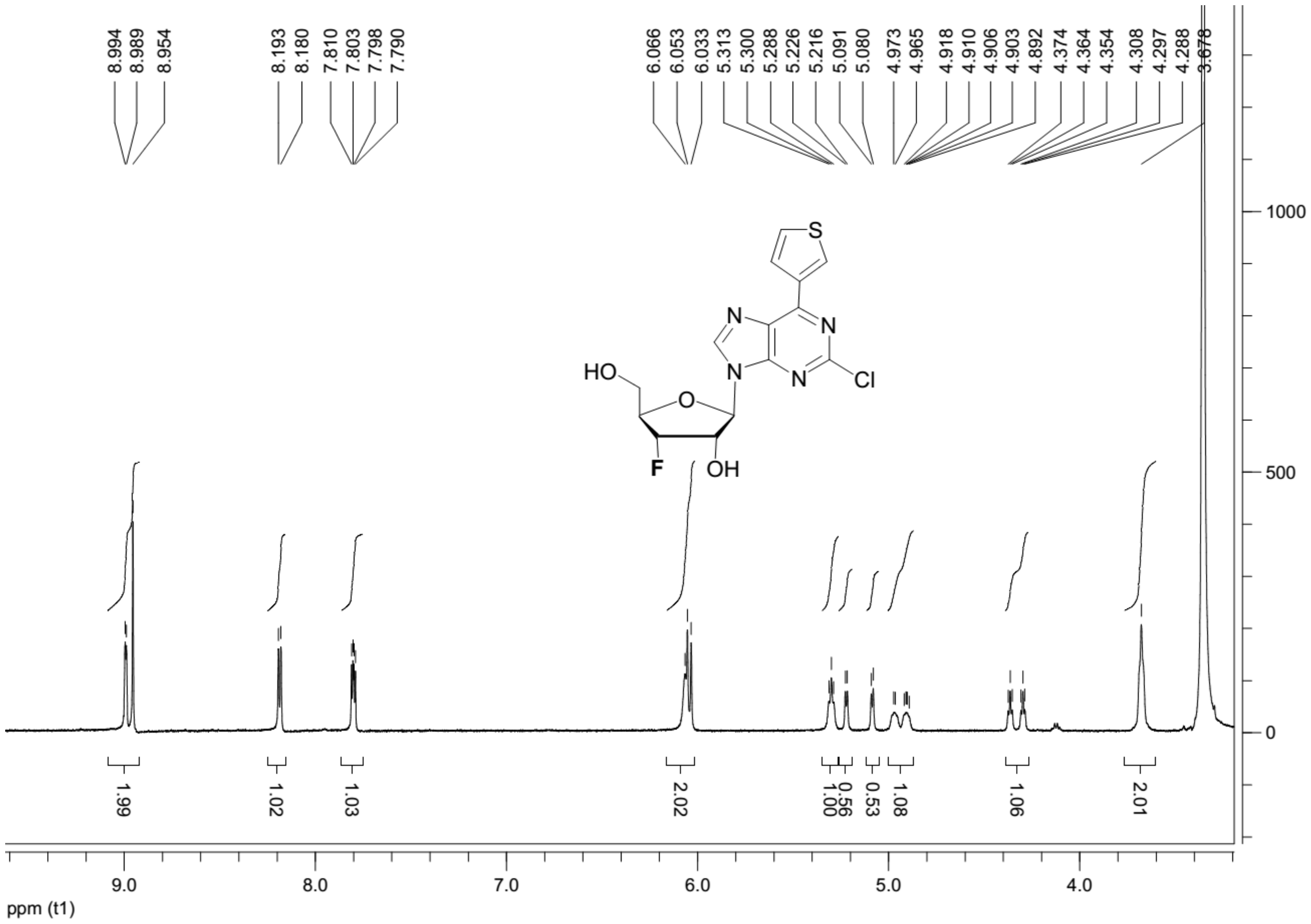

Product 18: HRMS

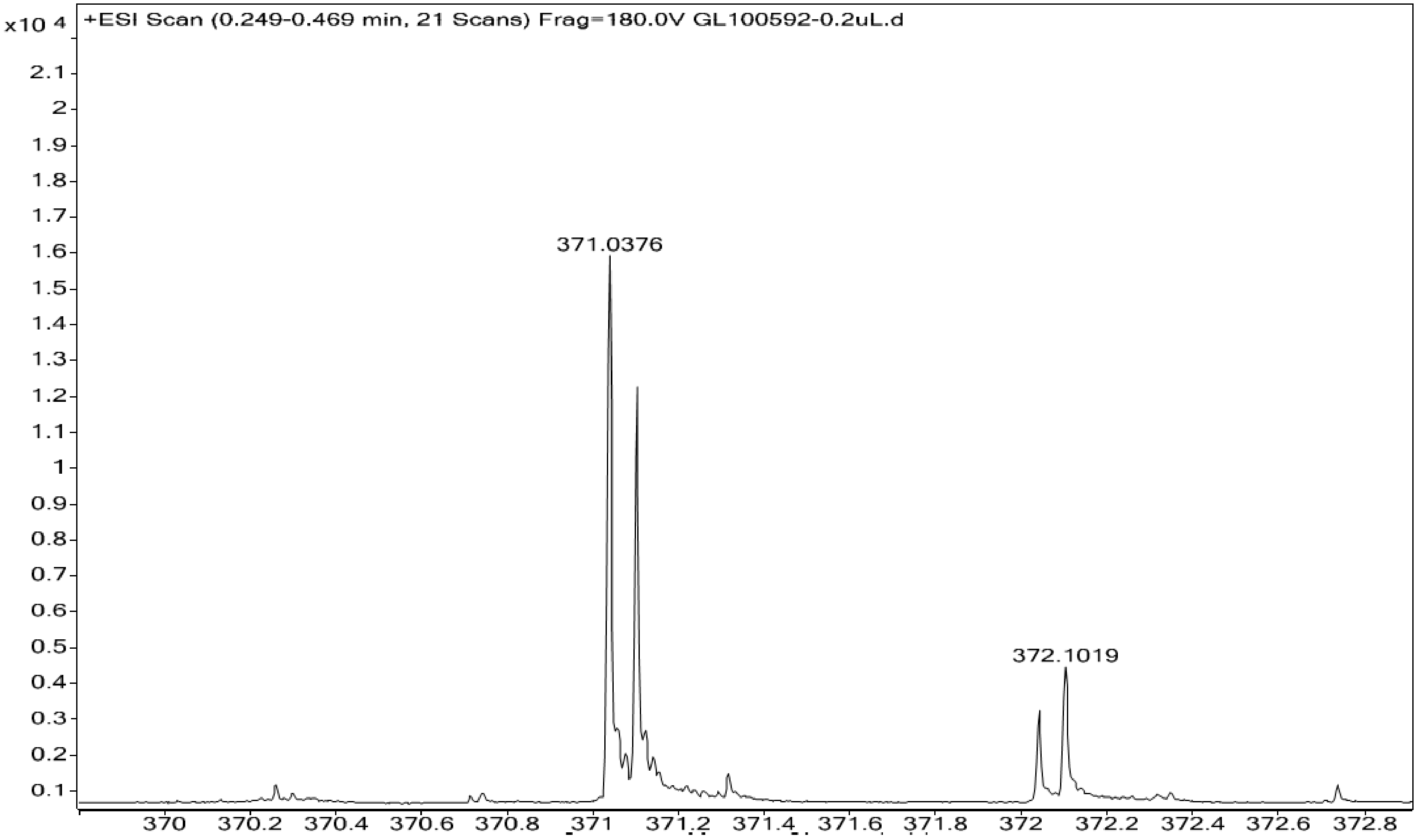

Product 19: HRMS

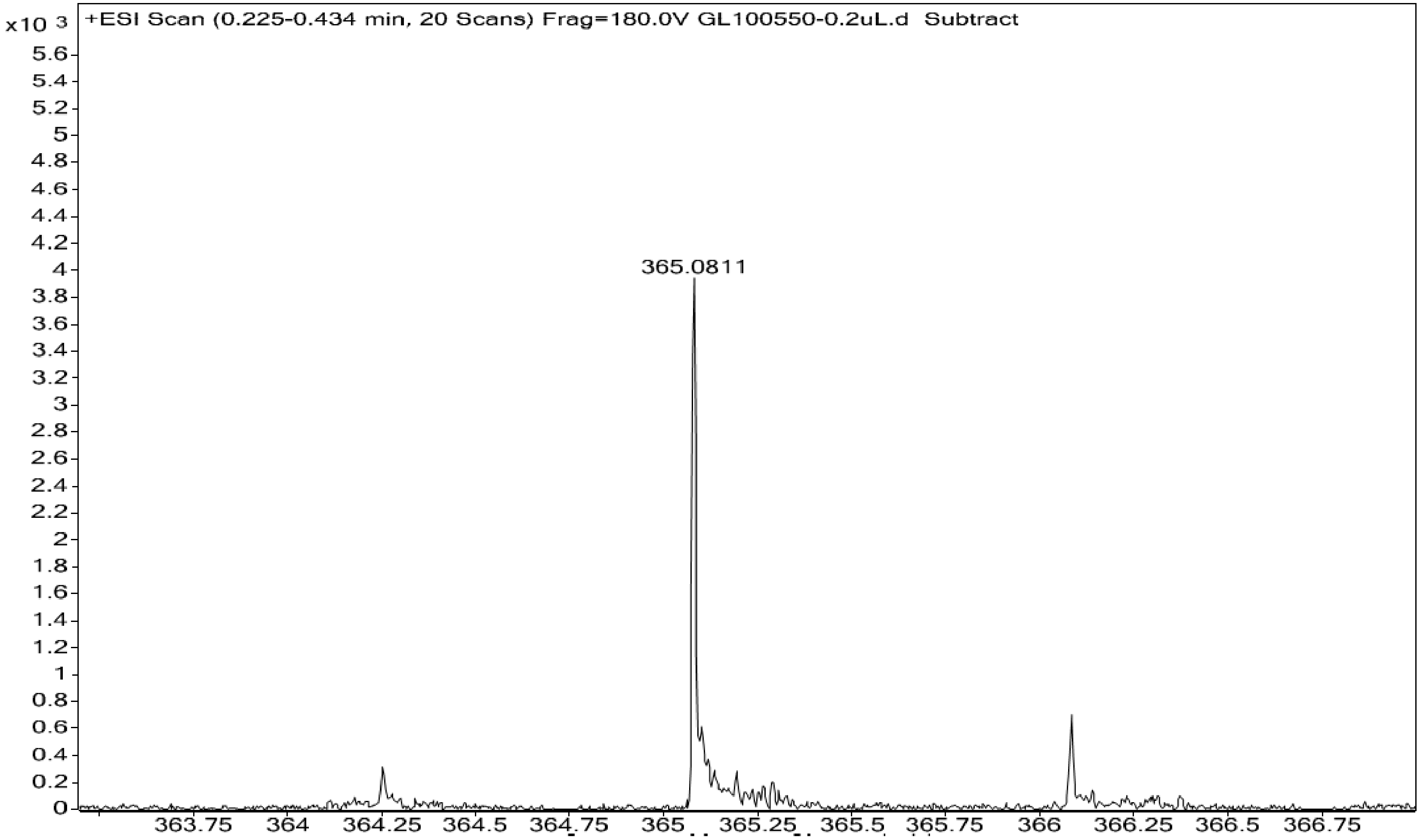

Product 20: HRMS

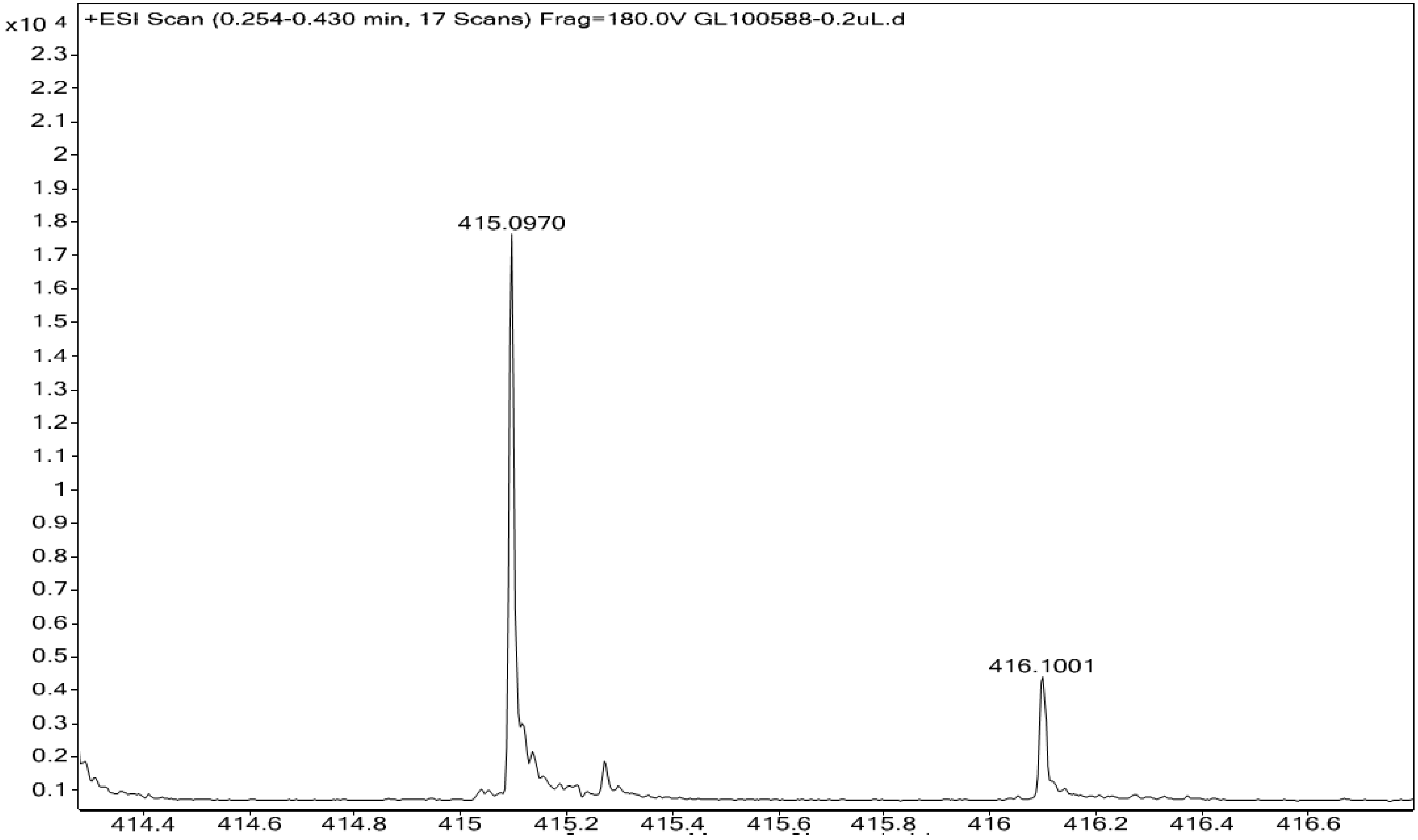

Product 21: ^{1}H NMR (400 MHz, DMSO-d_6)

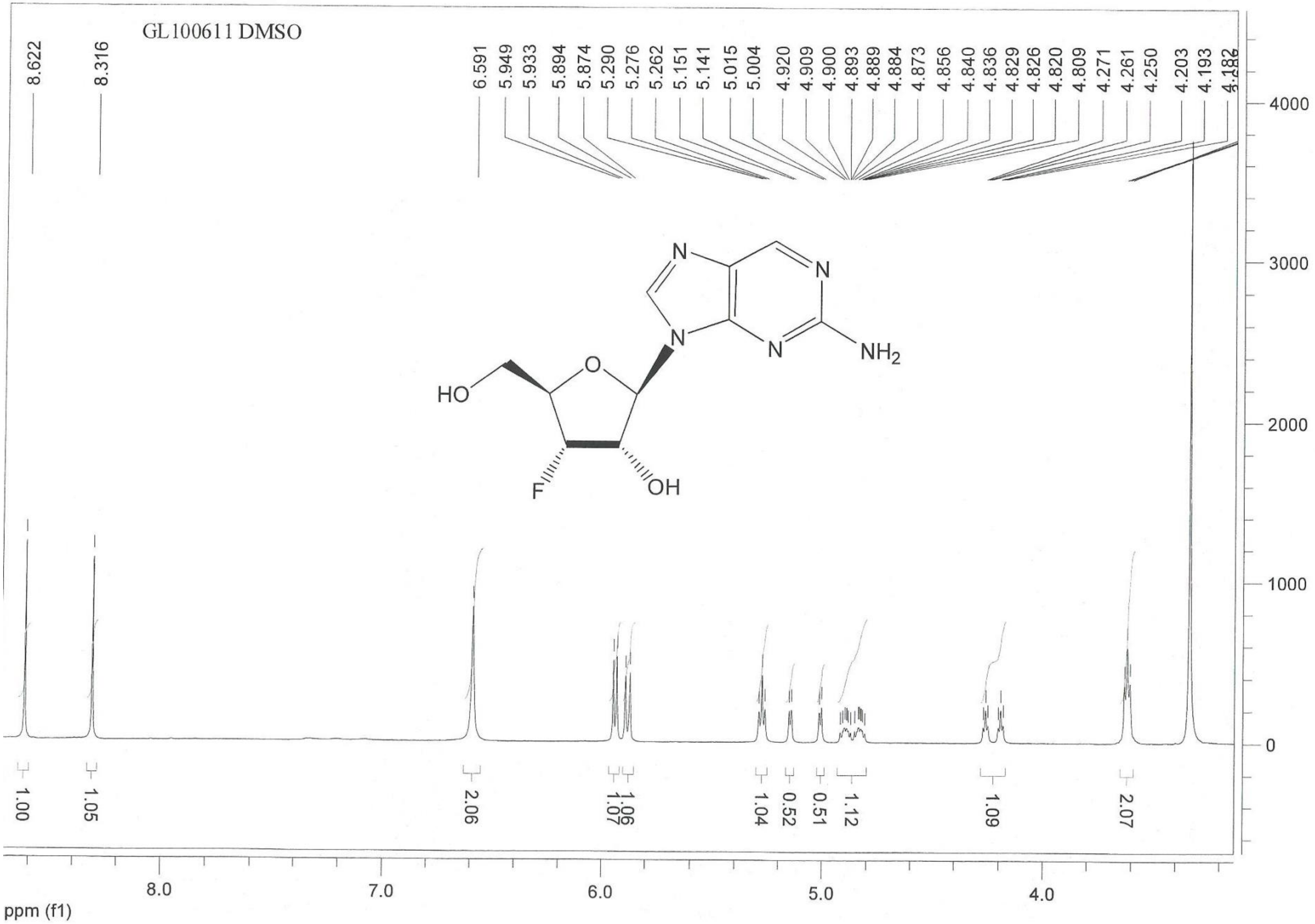

Product 21: HRMS

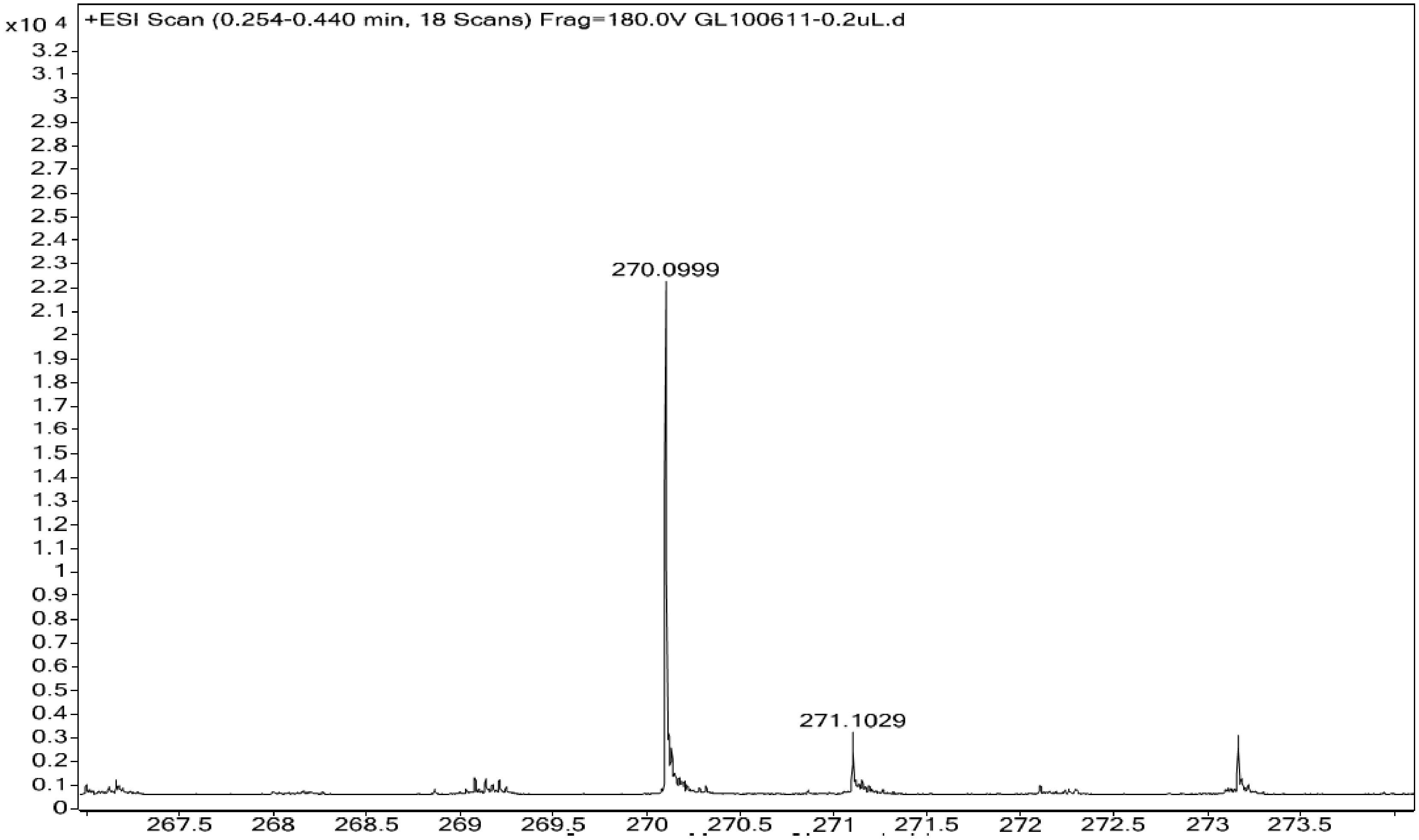

Product 22: ^{1}H NMR (400 MHz, DMSO-d_6)

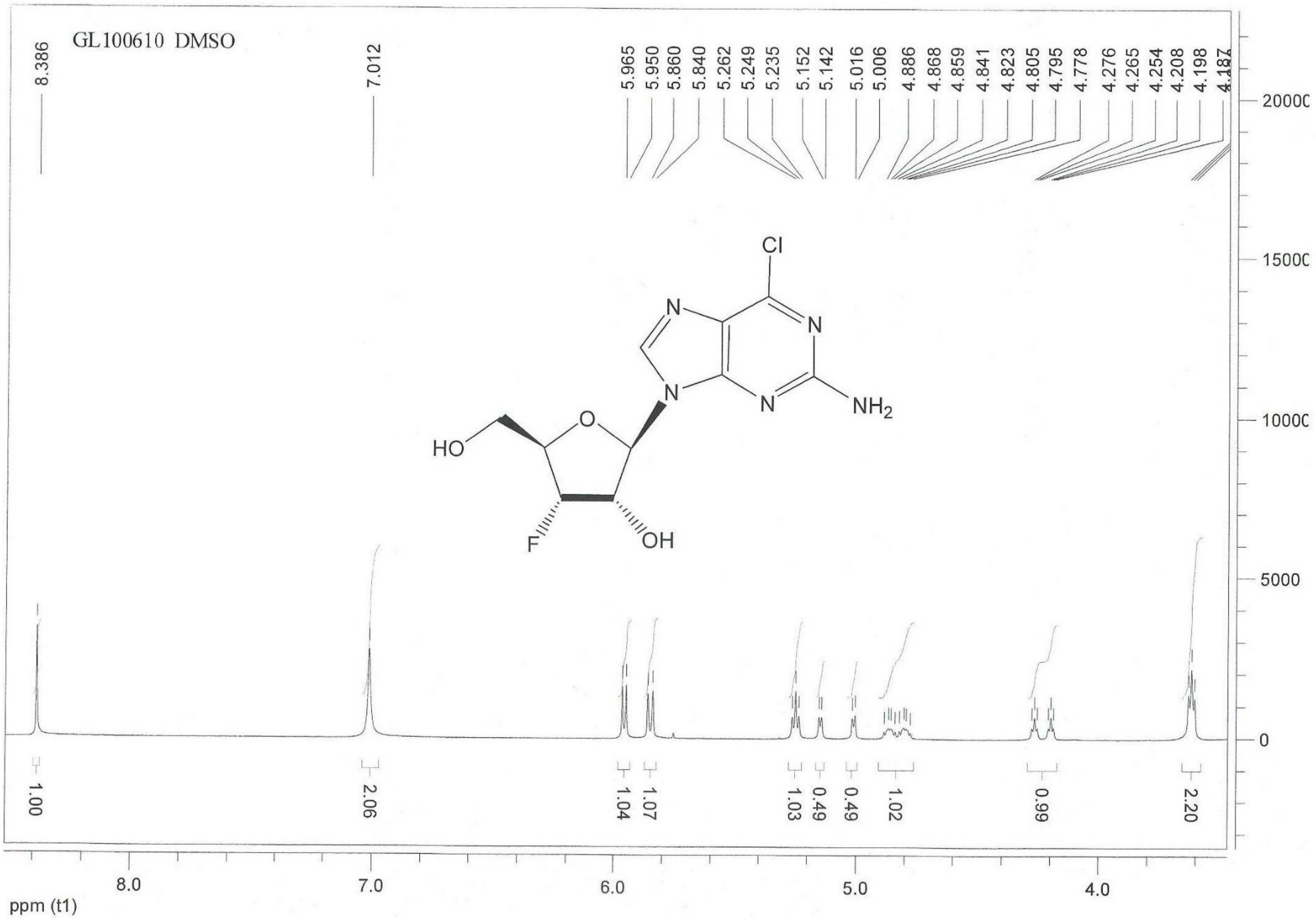

Product 22: HRMS

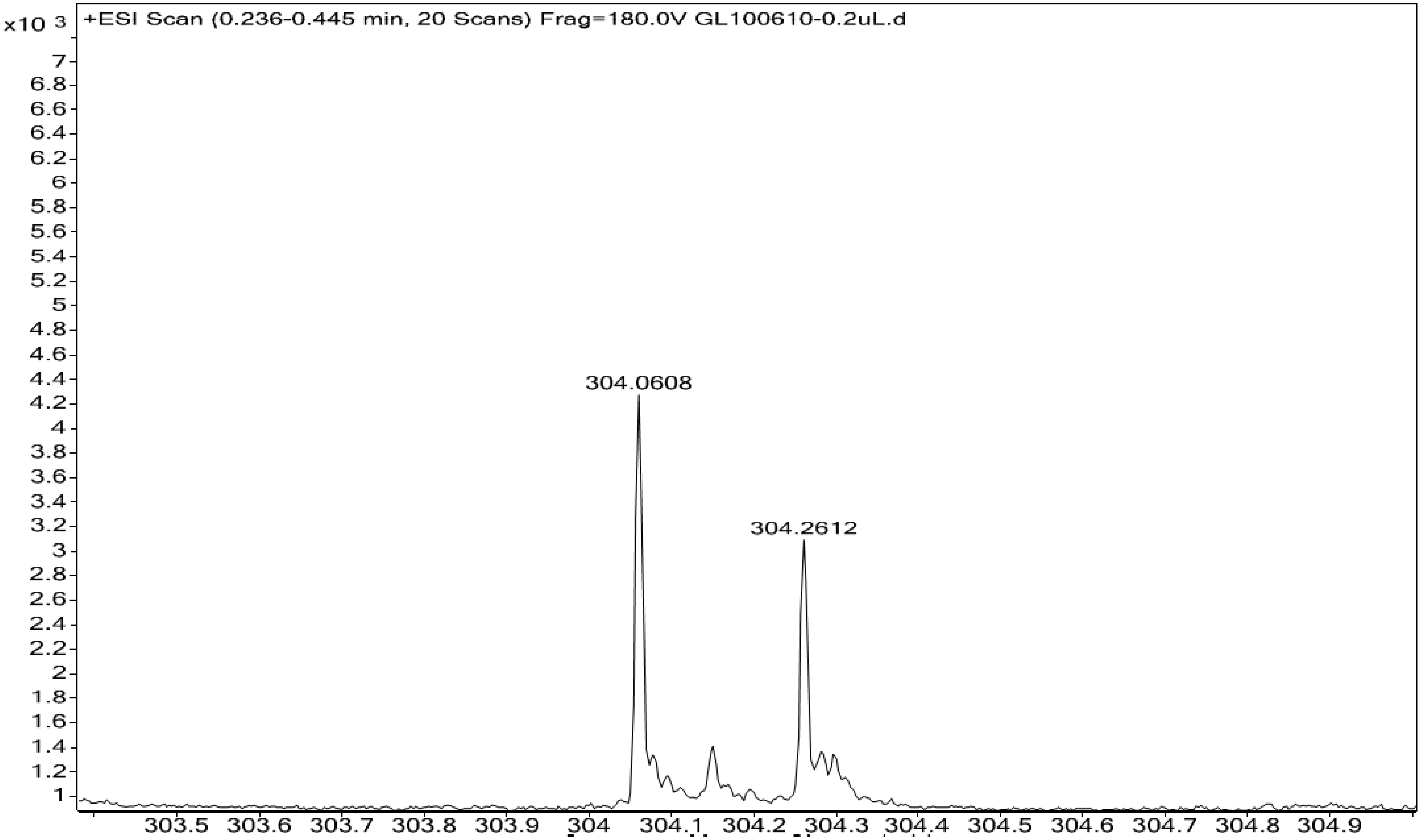

Product 23: ^{1}H NMR (400 MHz, DMSO-d_6)

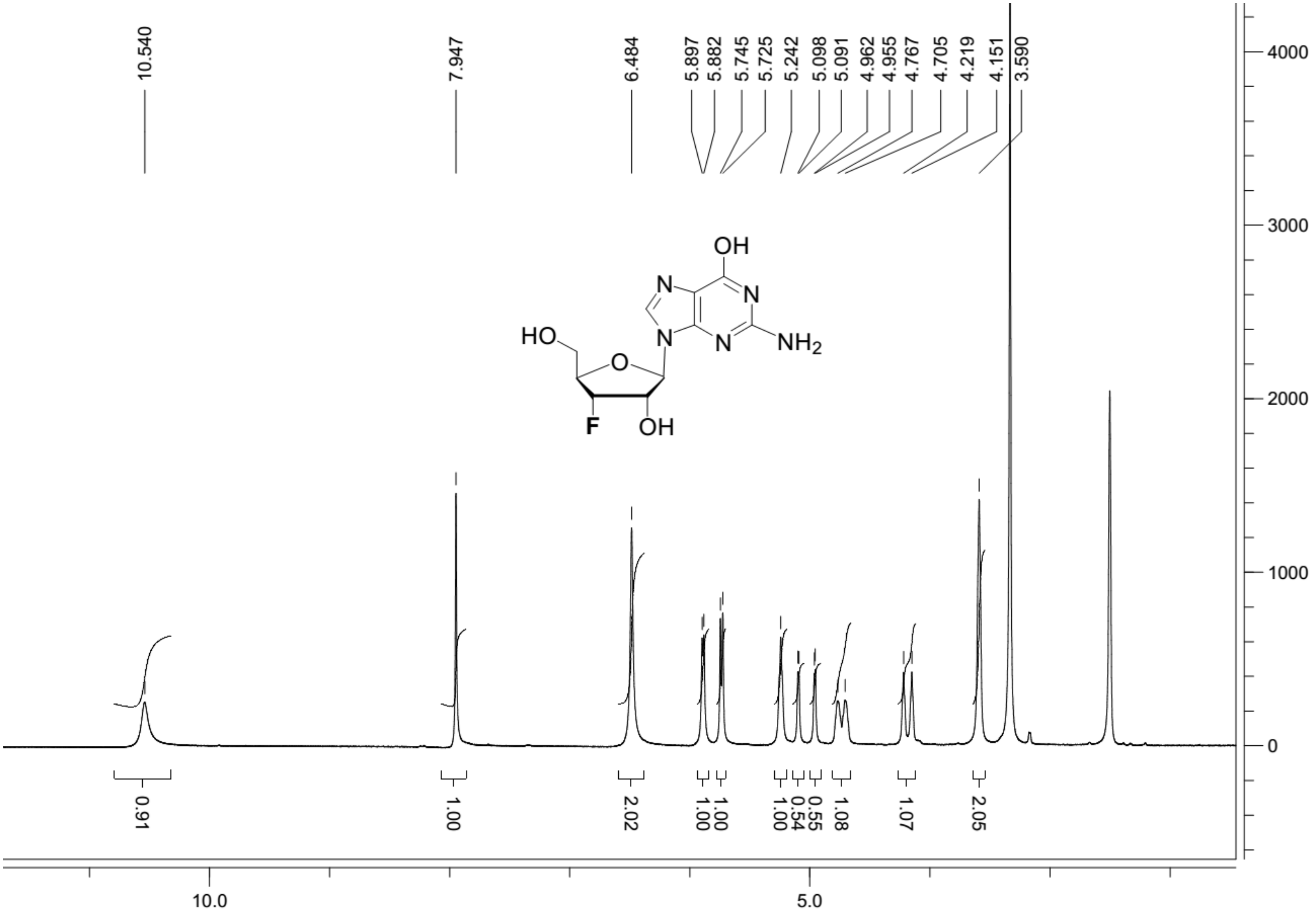

Product 23: HRMS

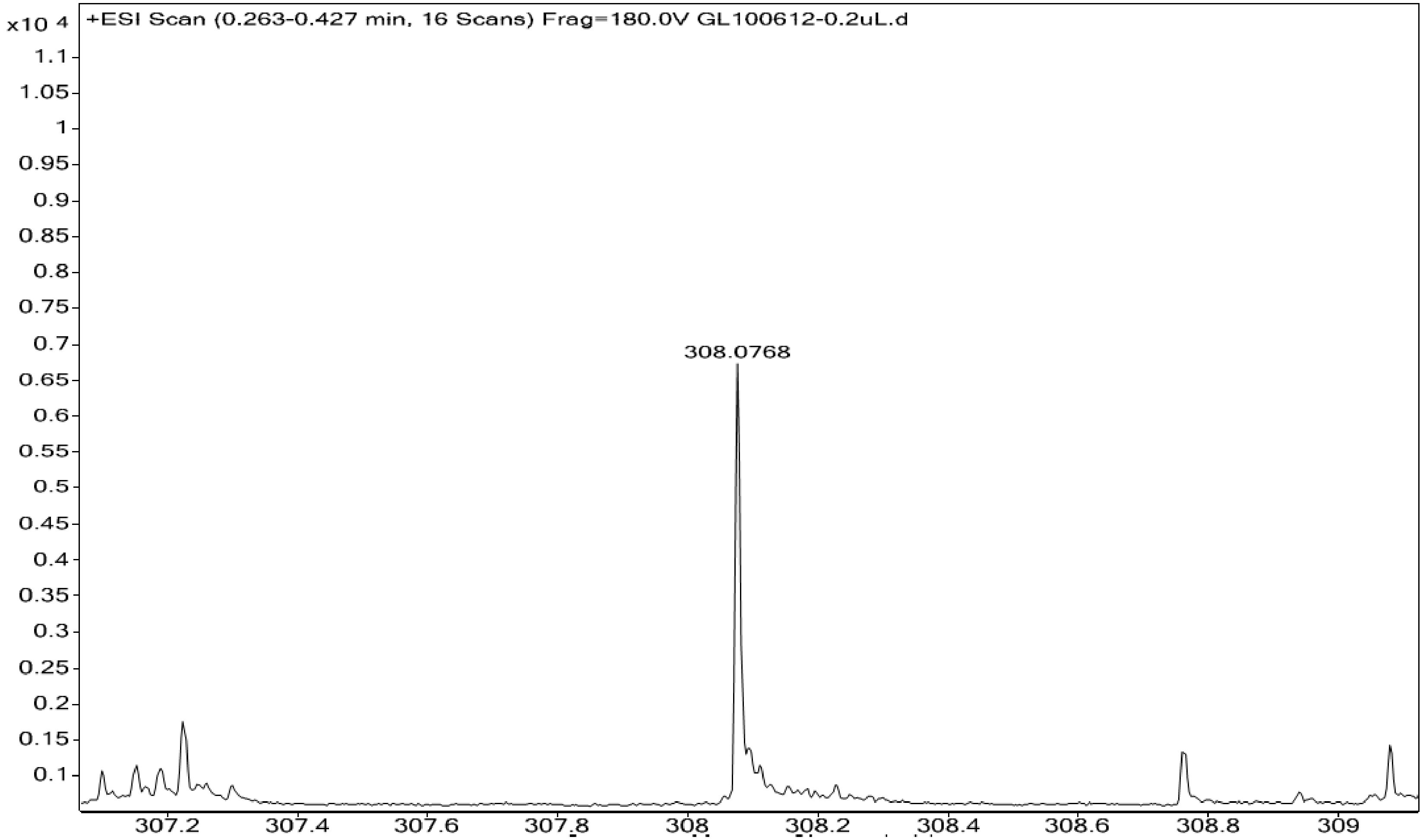

Product 26: ^{1}H NMR (400 MHz, DMSO-d_6)

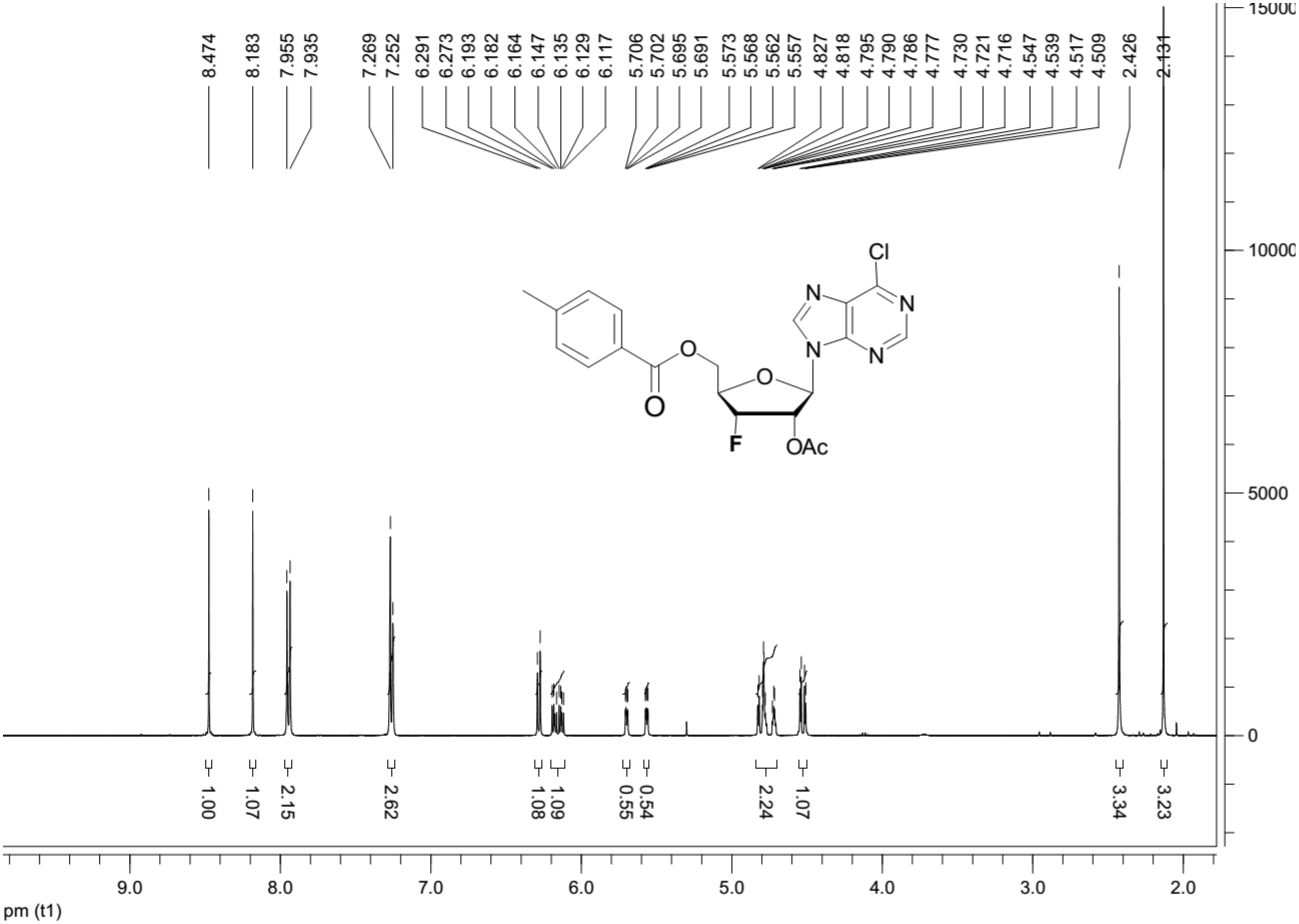

Supplement: File 1 — Experimental procedures, characterization data, and 1H NMR and mass spectral data for new compounds. [file Beilstein_J_Org_Chem-11-2509-s001.pdf]
